# Artifact management methodologies for arterial blood pressure signals: A systematic scoping review of human and animal literature

**DOI:** 10.14814/phy2.70533

**Published:** 2025-10-02

**Authors:** Tobias Bergmann, Nuray Vakitbilir, Xue Nemoga‐Stout, Amanjyot Singh Sainbhi, Kevin Y. Stein, Noah Silvaggio, Rakibul Hasan, Jaewoong Moon, Mansoor Hayat, Logan Froese, Frederick A. Zeiler

**Affiliations:** ^1^ Department of Biomedical Engineering, Price Faculty of Engineering University of Manitoba Winnipeg Manitoba Canada; ^2^ Undergraduate Engineering, Price Faculty of Engineering University of Manitoba Winnipeg Manitoba Canada; ^3^ Undergraduate Medicine, Rady Faculty of Health Sciences University of Manitoba Winnipeg Manitoba Canada; ^4^ Department of Human Anatomy and Cell Science, Rady Faculty of Health Sciences University of Manitoba Winnipeg Manitoba Canada; ^5^ Section of Neurosurgery, Department of Surgery, Rady Faculty of Health Sciences University of Manitoba Winnipeg Manitoba Canada; ^6^ Department of Clinical Neuroscience, Karolinska Institutet Stockholm Sweden; ^7^ Centre on Aging University of Manitoba Winnipeg Manitoba Canada; ^8^ Division of Anaesthesia, Department of Medicine, Addenbrooke's Hospital University of Cambridge Cambridge UK; ^9^ Pan Am Clinic Foundation Winnipeg Manitoba Canada

**Keywords:** arterial blood pressure, artifact management, bio‐signal analysis, hemodynamic monitoring

## Abstract

Arterial blood pressure (ABP) is a broadly measured vital signal used to monitor cardiovascular health through raw signal and derived metrics. Artifacts are a pervasive issue, resulting in a declined utility of the signal. The aim of this review was to examine the existing literature pertinent to artifact management strategies in ABP signals. A search of five databases was conducted based on the Preferred Reporting Items for Systematic Reviews and Meta‐Analysis guidelines. The search question examined existing algorithms for artifact management in ABP signals. The initial search yielded 15,564 articles (update included 1967 additional). The review included 73 articles. Categories included: (1) artifact management in low‐frequency signals, (2) artifact management in full‐waveform signals, (3) identification of valid or fiducial points. There were several algorithms that achieved strong effectiveness results. However, there was poor algorithm generalizability and inter‐comparability due to insufficient population size, diversity in recording methodologies, and external validation. This made it difficult to quantitatively elucidate a leading method. Real‐time utility was also not mentioned, critical for clinical use. This work provides novel insights into the literature regarding artifact management in ABP, identifying shortcomings and ways in which a more generalizable solution can be identified.

## 1 INTRODUCTION

Arterial blood pressure (ABP) is among the most broadly measured vital signals, impacting prognoses and diagnoses in acute and long‐term healthcare settings on a daily basis (McGhee & Bridges, 2002; Nguyen & Bora, 2024; Rehman & Hashmi, 2025). Invasive blood pressure (IBP), often described as the “gold‐standard” technique, involves the direct measurement of pressure in different peripheral arteries (Goodman & Kitchen, 2023; Kaki et al., 2018; Nguyen & Bora, 2024; Noh et al., 2024; Nuttall et al., 2016). IBP monitoring, via an arterial line, is used in a variety of circumstances including critical illness monitoring, anesthesia titration, surgical procedures, and regular blood draws (Frezza & Mezghebe, 1998; Gardner, 1981; Kaki et al., 2018; Nguyen & Bora, 2024; Scheer et al., 2002). The measurement of the ABP signal is required in an intensive care unit (ICU) as a component of basic hemodynamic monitoring. It provides early detection and enables intervention in instances of patient risk of inadequate perfusion or oxygen delivery; detection is often in the form of alarms in online monitoring (Borowski et al., 2011; Kuhn & Werdan, 2001). Despite its utility, there are several limitations of IBP, including the level of training involved to insert the device into the artery, as well as potential pathological complications due to the invasive nature of this method (Goodman & Kitchen, 2023).

To broaden the utility of blood pressure (BP) beyond the ICU, several non‐invasive blood pressure (NIBP) methods have been developed based on initial work by Korotkoff in 1905 (Korotkoff, 1905; Lewis on behalf of the British and Irish Hypertension Society's Blood Pressure Measurement Working Party, 2019), which paved the way for discrete non‐invasive measurement of systolic blood pressure (SBP) and diastolic blood pressure (DBP) (Lewis on behalf of the British and Irish Hypertension Society's Blood Pressure Measurement Working Party, 2019). However, the utility of BP is not only in the magnitude of the signal. The ABP waveform itself provides additional insights into the functionality and health of the cardiovascular system, including properties of the blood vessel walls (Miyakawa et al., 1984; O'Rourke et al., 1992; Zong et al., 2003). In a typical ABP waveform, there are several points of interest, each corresponding to anatomical processes described using morphology and temporal location (Abushouk et al., 2023; Pal et al., 2024; Singh & Sunkaria, 2017). Thus, a methodology for capturing non‐invasive full waveform ABP measurement is of great clinical value. This process is enabled using technologies developed using the Peñáz methodology; this relies on an external pressure provided by a cuff with a built‐in light source capable of measuring arterial volumetric changes (Boehmer, 1987; Peňáz, 1973; Wesseling, 1990). Full‐wave ABP signals have been broadly used across cardiovascular critical care to derive additional metrics, including estimated cardiac output (Sun et al., 2005), arterial compliance, and arterial resistance (Sun et al., 2006). Developments within NIBP technology have allowed for these full‐waveform metrics to be applied non‐invasively.

Continuous ABP signals are also critical in guiding neural injury treatment and analyzing cerebral vascular health. ABP signals are leveraged in conjunction with intracranial pressure (ICP) to derive cerebral perfusion pressure (CPP) as well as other more complex continuous metrics, including pressure reactivity index (PRx) (Czosnyka et al., 1997) and optimal CPP (CPPopt) (Steiner et al., 2002). Derived metrics help improve the understanding of the interrelationships between cerebral bio‐signals, helping elucidate how a change in one physiological process may affect the other. Additionally, metrics such as CPPopt help provide patient‐specific targets to improve outcomes through individualized optimal care (Beqiri et al., 2023; Liu et al., 2017; Steiner et al., 2002). However, a critical limitation of ABP signals and their derived measures is the signal artifacts that occur during recording.

Artifacts in ABP signals can result from a variety of sources that occur during invasive and non‐invasive measurement. During invasive measurement, artifact sources include over‐ or under‐dampening in catheter‐based monitors, catheter failures, and extrinsic patient movement (Li et al., 2009; McGhee & Bridges, 2002). For non‐invasive measurement, common artifact sources include motion artifacts (Deegan et al., 2007; Fortin et al., 2021), intermittent changes in cuff inflation (Keselbrener & Akselrod, 1995), and vibrations in the measurement environment (Alpert et al., 2020; Germanó et al., 1991). Each of these artifacts can be widespread during monitoring and take different morphological forms (Li et al., 2009), necessitating unique and specialized methods of artifact detection.

Kim et al. (2018) identified that incidences of bradycardia and tachycardia were significantly reduced with the reduction of artifacts using machine learning. While it has not been widely studied, these results suggest that the presence of artifacts has significant implications on patient outcomes. The presence of artifacts also exacerbates pervasive “alarm fatigue” faced by nurses, in which they are overwhelmed by the number of alarms regularly produced, particularly those that are nonactionable (Nyarko et al., 2024). These artifact‐driven false alarms contribute to “burnout” and desensitization faced by nurses, which leads to a detachment from patient care, thus resulting in a decline in quality (Lewandowska et al., 2020; Nyarko et al., 2024). While many of these false alarms may be the result of alarm thresholds being improperly set by staff, ABP signal artifacts are also a contributor. The reduction of signal artifacts could greatly alleviate some of the aforementioned burden. The impact of artifacts on the accuracy of ABP‐derived metrics in critical care has not been thoroughly examined; however, work by Rozanek et al. (2022) indicates that the presence of artifacts can have significant implications on the accuracy of PRx and its utility as a clinical tool. Sun et al. (2005) also warn about the implications of artifacts on estimated cardiac output. As such, the ability to remove artifacts is paramount to enable improved accuracy in diagnoses and prognoses, a reduction in alarm fatigue, and improved accuracy in derived metrics. This can facilitate an improved understanding of cardiovascular physiology and have potential prognostic capabilities in the future.

The current best practice is to identify and remove artifacts manually, which is incredibly tedious and labor‐intensive (Rozanek et al., 2022). The time lag required for human annotation prohibits any kind of live‐time use of artifact‐free data during patient care. As a result, there has been a movement toward the automated or semi‐automated identification and removal of artifactual data segments or the identification of valid data segments using various algorithms. However, a “gold‐standard” method for semi‐ or fully automated artifact management of ABP signals has remained elusive (Rozanek et al., 2022). The question that this review sought to answer was: What artifact management techniques exist for ABP signals? The objective of this systematic scoping review is to identify pertinent literature presenting the algorithms fulfilling the aforementioned functions with quantitative evaluations of their efficacies. A secondary objective is the potential elucidation of a leading method for this application.

An ideal methodology for this application is required to be able to adequately identify artifacts with varied morphologies. As such, it should be tested on sufficiently large datasets composed of patients with differing physiology/injury patterns as well as using different recording technologies (invasive and non‐invasive). False positives must be limited such that a rich data stream can be maintained when artifacts are removed. The algorithm must also be adequately efficient such that clean data streams can be leveraged in real time. It is on the basis of these criteria that algorithms presented in the current literature are evaluated in this work. The presented review serves as a novel and crucial contribution to the field, as there currently exists no current and comprehensive literature source evaluating automated artifact management methods for a vital signal that is the basis of critical clinical decisions.

## 2 METHODOLOGY

This manuscript details a systematic scoping review that was conducted following the methodology outlined in the Cochrane Handbook for Systematic Reviews (Page et al., 2021). The results of the review were presented following the Preferred Reporting Items for Systematic Reviews and Meta‐Analysis guidelines with the PRISMA Extension for Scoping Review (Page et al., 2021; Tricco et al., 2018). The review objectives as well as the corresponding search strategy were developed collaboratively by TB and FAZ. TB, NV, LF, XNS, and KYS contributed to the article filtering process. The completed PRISMA checklist can be found in File S1.

### 2.1 Ethical considerations

The articles included and examined as part of this review were from previously published journals. As such, they are expected to have been vetted by these journals. This made it unnecessary to conduct any specific ethics approval review.

### 2.2 Search question and inclusion/exclusion criteria

The main question examined within this review was: “What artifact management techniques exist for ABP signals?” Additional secondary questions focused on the effectiveness of each of the techniques included in the manuscripts reviewed, the types of algorithms that were used to identify and remove the artifacts, and the context for which they were designed. The specific ABP recording hardware used to collect data to develop each of these methods was recorded, as well as whether the data were collected invasively or non‐invasively was also considered. The articles included in this review were required to detail some form of artifact identification or removal method for full waveform ABP signals collected from either human or animals. Articles that were excluded were non‐English, review articles, not peer‐reviewed, or did not include empirical evidence of artifact identification effectiveness. Additionally, articles which presented algorithms that had been trained/tested/validated solely on fully simulated ABP signals, real ABP signals with simulated artifacts, or simulated ABP signals with real artifacts introduced were not included as they do not represent true ABP signals.

Continuous monitoring of ABP signals occurs at varying sampling frequencies, largely dictated by whether full‐waveform data are needed. Pal et al. (2024) described five different fiducial points on the ABP pulse; these include the systolic phase onset, the systolic peak, the dicrotic notch, the diastolic peak, and the diastolic phase endpoint. However, it should be noted that these points are referred to under different names across literature. To properly encapsulate the waveforms of each of these points and properly reconstruct the ABP waveform, it is critical to select a sufficient sampling rate. There currently exists little standardization in sampling rate for full‐waveform ABP. According to the American Heart Association guidelines for validation of non‐invasive arterial pulse wave velocity measurement devices, for ABP signals, the sampling rate necessary to capture the 20th harmonic of the approximately 60 Hz heart rate signal is 120 Hz, adhering to the Nyquist criterion (Spronck et al., 2024; Vlachopoulos et al., 2011). This standard is consistent with Aboy et al. (2005), wherein the authors found that 125 Hz is sufficient for pressure pulse contour analysis in the absence of any cardiac arrhythmias. However, there is no evidence to support that sampling rates slightly below 120 Hz would not properly encapsulate the full waveform. As such, in this review, full‐waveform ABP signals are described as those with a sampling rate ≥100 Hz, and all signals sampled below 100 Hz are considered low frequency.

### 2.3 Search strategy

Searches were conducted across five databases, which included BIOSIS, SCOPUS, EMBASE, PubMed, and Cochrane Library. The initial search results covered the entire period from the conception of each database up to August 27th, 2024. Dedicated search strings were constructed and used to search these databases. The search string consisted of terms/synonyms for ABP as well as artifact identification and removal methods. The detailed search strategy is provided in the File S2. The results of the search from each database were compiled, and the results were deduplicated such that only unique results were included in the review. The search was then updated on May 14, 2025, using the same search string for the same five databases. BIOSIS and PubMed were searched for articles published after August 27, 2024, Cochrane Library was searched for articles published after August 2024, and EMBASE and SCOPUS were searched for articles published after 2024. The differences in the search dates were limitations of the capabilities of each database search function.

### 2.4 Study selections

A review of all unique articles from the initial search was manually conducted using a two‐stage, two‐reviewer approach. During the first stage, two reviewers (TB and NV) independently screened the title and abstract based on eligibility defined by inclusion/exclusion criteria. The included studies were then screened in full length by both reviewers based on the same inclusion and exclusion criteria. Any disagreements between the two reviewers were resolved by a third party (LF). The updated screening was conducted by two reviewers (TB and XNS); any discrepancies were resolved by KYS.

### 2.5 Data collection

Data collection was conducted by the primary author (TB). Characteristics about each study were recorded for analysis. These included the main purpose of the study and specific patient/subject demographic and data information. Patient/subject demographic information included the sample size, sex, age, and presence of underlying pathology. Data information included the sampling rate of the ABP signal, whether the signal was invasively or non‐invasively recorded, the anatomical placement of the device on the subject as well as the type of ABP hardware used, and any bio‐signals being simultaneously recorded and leveraged for artifact management. Information regarding the details of ABP signal artifact identification or removal methods described in each included manuscript were also extracted. Analysis of the described methods involved the evaluation of their effectiveness (or effectiveness relative to other presented methods) as well as the main study results, the data sources that were used to build the model, and the validation of the methods that were conducted. Additionally, the type of ABP signals (invasive or non‐invasive), and the pathophysiological health of the subjects from which the data was recorded were also considered. Limitations for each method were also extracted, including those identified by the authors and those prevalent during the critical analysis of each article. The data that was extracted from each of the articles is presented in Files S3–S5.

## 3 RESULTS

**FIGURE 1:**
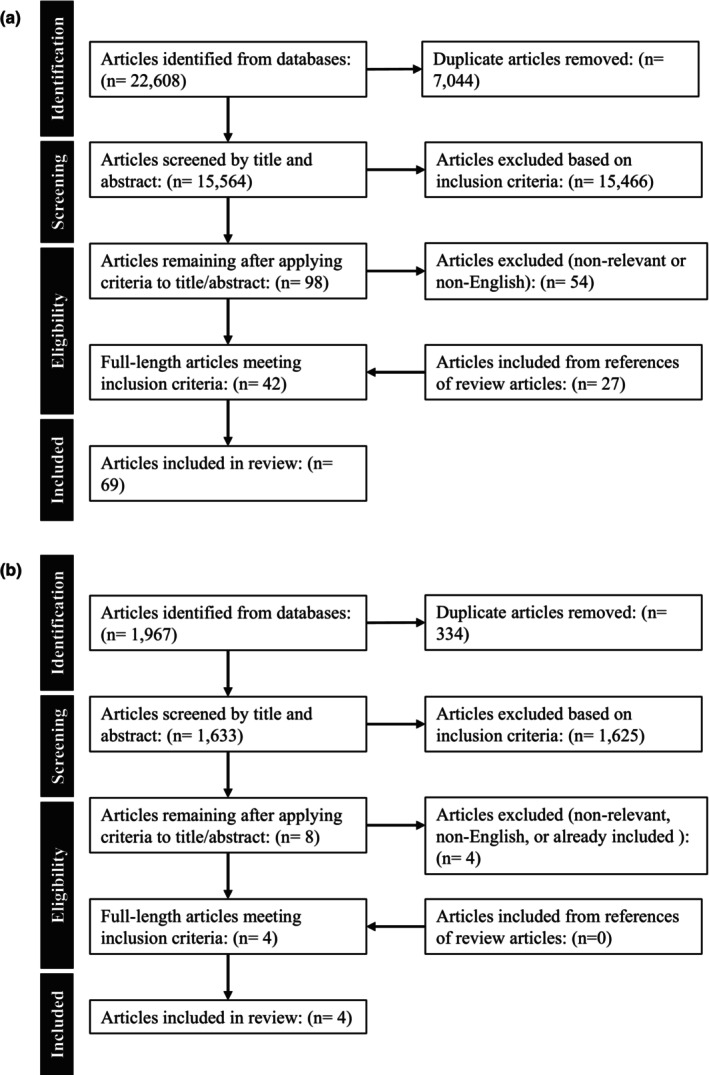
PRISMA flowchart for systematically conducted scoping review from searches on (a) August 27, 2024 and updated on (b) May 14, 2025.

The results of the search across BIOSIS, SCPOUS, EMBASE, PubMed, and Cochrane Library databases yielded 22,608 results. The deduplication process revealed that there were 15,564 unique articles to be included in this review. Following the first screening phase, 98 articles were deemed eligible based on inclusion/exclusion criteria. The number of articles that were deemed eligible based on full‐length screening was 42. An additional search through the reference sections of the included articles led to the inclusion of 27 more articles. The initial updated search yielded 1967 results. The deduplication process conducted revealed that there were 1633 unique articles included in this review. Following the first screening phase, there were eight articles deemed eligible based on inclusion/exclusion criteria. The number of articles that were deemed eligible based on full‐length screening was four. An additional search through the reference yielded no more articles. There were four more articles added in the updated screening. As such, there is a total of 73 published works that present methods that quantifiably address valid or artifactual ABP signals included in this review. These are depicted in a PRISMA flow diagram in Figure 1a,b.

**FIGURE 2:**
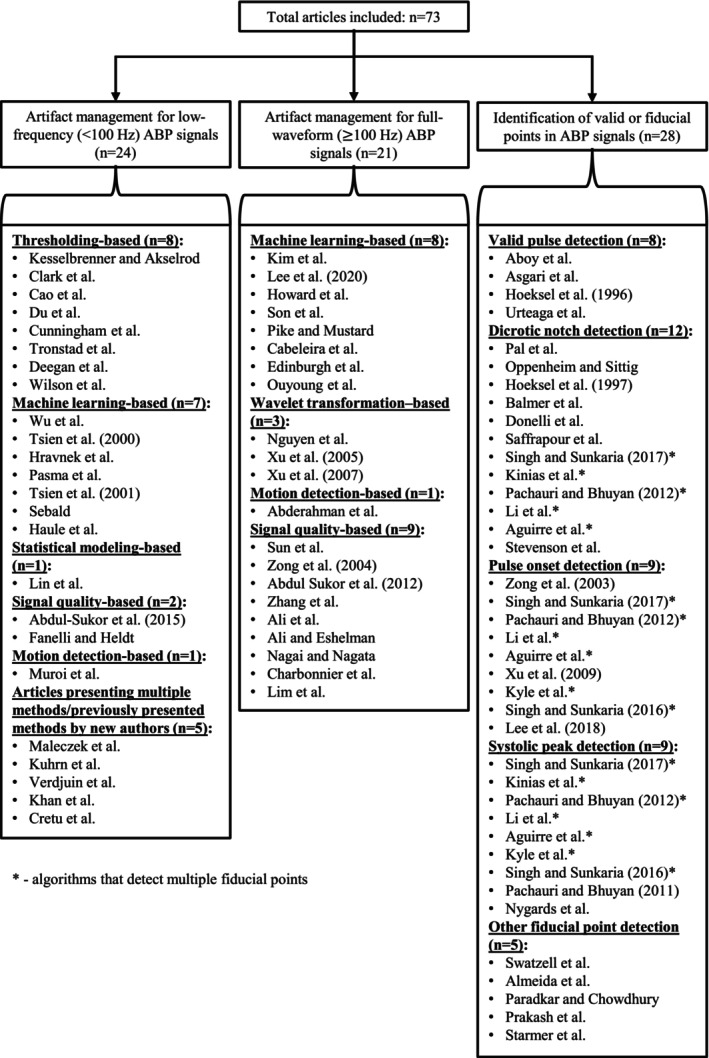
Sub‐categorized articles presenting artifact management methods or methodologies for valid/fiducial point detection.

There were 68 studies that were included that were human‐based, 4 animal‐based, and 1 with both human and animal subjects. In each of these articles, one or more algorithms have been developed for blood pressure signals to conduct: (1) artifact management in low‐frequency ABP signals, (2) artifact management in full‐waveform ABP signals, and (3) identification of valid or fiducial points in ABP signals. Figure 2 displays the sub‐categorical organization of the methods encapsulated in this review, organized by the methodologies employed for either artifact management or identification of valid or fiducial points in ABP signals.

### 3.1 Artifact management in low‐frequency ABP signals

There are several applications for ABP signal recordings that do not require full‐waveform ABP signals but nonetheless require artifact management. Twenty‐three articles presented algorithms capable of artifact detection in low‐frequency ABP signals, with 22 of them presenting novel methods and 2 implementing a previously developed algorithm on a new dataset. A summary of the function and methodology of each algorithm as well as detailed descriptions and additional results are included in File S3, Table SC.1–SC.6. Also included is pertinent information regarding the signal type, population size, sampling rate, and auxiliary signals recordings required for each method. The effectiveness of the developed algorithm on data collected on specific populations (with pathophysiology listed, if relevant) are also listed in Table 1. Imhoff et al. (1998) published a work that discussed two methods for artifact detection that used a phase space‐based and autoregression model‐based method. However, this article could not be included in the review as it presented its effectiveness of artifact identification as a combined metric from its performance on heart rate, MAP, and pulmonary artery pressure. Because the performance on blood pressure specifically could not be distinguished, it was not included.

**TABLE 1:** Summary of artifact management methods in low‐frequency ABP signals.

Method type	Signals used	Sampling rate	Median population size	Effectiveness on population/dataset	Validation methods used
Threshold‐based (*n* = 8)	IBP (*n* = 1) NIBP (*n* = 3) Non‐specific ABP (*n* = 3) Both NIBP/IBP (*n* = 1)	<1 Hz (*n* = 4) >1 Hz (*n* = 4)	12.5 (min = 2, max = 54)	Keselbrener & Akselrod (1995) indicated a decrease in spectral power in LF and HF bands of 146 to 140 and 74 to 35 in unspecified units of power, respectively, in a healthy adult, and decrease from 154 to 119 in LF band for a healthy child before and after the use of the algorithm Clark et al. (1987) indicated a 95.6% reduction in ABP readings that a clinician had to address in patients with mild hypertension compared to no use of the algorithm **Cao et al. (** 1999 **) indicated their artifact detection algorithm that used physiological thresholds, statistical deviation, and correlation deviation from a coincidently recorded HR signal had a sensitivity of 94.2% (SD = 5.3%) and specificity of 80.0% (SD = 12.4%) on preterm infants compared to annotations conducted by a “domain expert”** Du et al. (2019) indicated artifact detection sensitivity of 87.0% and specificity of 99.4% on patients undergoing surgery compared to manual review by an anesthesiologist Cunningham et al. (1994) reported a median and mean difference between manual and automatic removal of artifacts of 1.15% and 1.48%, respectively. Manual removal was conducted by three experts, likely with access to coincidently recorded tcpO_2_, HR, and RR signals Tronstad et al. (2015) analyzed performance of their artifact management method on IBP and NIBP achieving precision results of 79% and 68%, respectively, and recall results of 94% and 97%, respectively, for each signal recorded in pregnant women undergoing a cesarean delivery with spinal anesthesia compared to annotations conducted by an expert with 20 years of experience with access to a coincidently recorded CO signal. The presented method outperformed the method proposed by Deegan et al. (2007) and Tronstad et al. (2015) Deegan et al. indicated a success ae of 99.38% with a FP rate of 0.02% when its artifact removal method was applied to individuals with orthostatic hypertension compared to annotations by an experienced clinician (Deegan et al., 2007) Wilson et al. (2015) employed a filtering process such that recorded NIBP signal values would correspond to manual measurement using a brachial cuff, yielding NIBP measurement with a 0.95 ± 5.88 mmHg	Direct testing (*n* = 8)
Machine learning‐based (*n* = 7)	IBP (*n* = 5) NIBP (*n* = 1) Non‐specific ABP (*n* = 1) NIBP/IBP (*n* = 1)	<1 Hz (*n* = 5) >1 Hz (*n* = 1) Not specified (*n* = 1)	102 (min = 1, max = 634) Not specified (*n* = 1)	Wu et al. (2020) indicated that the XGBoost standard ML model had the best results compared to time‐series forecasting and other ML‐based methods achieving a precision and recall of 99.98% and 99.97% using a multivariate approach and a precision and recall of 99.74% and 99.33% using a univariate approach on data collected during overnight surgery. Model performance was compared to annotations conducted by two anesthesiologists that were able to consider coincidentally recorded HR, SpO_2_, and ECG signals **Tsien et al. (** 2000 **) developed two ML models using BP and auxiliary signals (ECG, HR, pO**_**2**_, **and pCO**_**2**_ **), the C4.5 decision tree model outperformed the linear regression model based on the testing dataset. It achieved a sensitivity of 57.7%, specificity of 99.9% and AUCROC of 89.4% in data recorded from ICU patients compared to annotations conducted by an experienced physician** **Hravnak et al. (** 2016 **) evaluated several ML methods in their application in artifact identification using multi‐signal pattern recognition leveraging concurrently recorded HR, RR, SpO**_**2**_ **as well as derived pulse pressure in SDU patients. The linear regression model achieved the highest AUCROC of 0.907 on training data, but the SVM (unspecified kernel type) was indicated to have the most consistent performance when applied to the validation set; however, exact quantification was not provided. Model performance was compared to manual annotations by two experienced clinicians** Pasma et al. (2021) indicated that a radial basis function SVM model had the best performance on patients undergoing surgical procedures achieving sensitivity, specificity, and PPV values of 0.521, 0.999, and 0.884, respectively. Using 1/5 Hz data, the results were similar achieving results for the same metrics of 0.518, 0.997, and 0.830. Model performance was compared to live and retrospective annotations conducted by experienced researchers.	Train/test/validation (*n* = 1) Train/parameter tuning/test (*n* = 2) Training with cross‐validation/test (*n* = 2) Train/test (*n* = 1) Train/test by patient (*n* = 1)
				**Tsien et al. (** 2001 **) evaluated the performance of a model constructed on one dataset being applied to another with a different sampling rate. Models were constructed using IBP data as well as HR, pCO**_**2**_ , **and pO**_**2**_ **sampled at 0.0167 Hz and 1 Hz and were to applied data of similar and different sampling rates. The AUCROC of the model constructed using 1 Hz applied to data sampled at 1 Hz and 0.0167 Hz from patients in the ICU was 100% compared to the manual annotation by a trained clinician** Sebald (1989) used a competitive learning topology, trained and tested using data from patients about to undergo cardiac surgery, achieving an error rate of 7% for the train set and 6% for the test set compared to manual characterization Haule et al. (2025) proposed a method combining β‐VAE and IF machine learning models, trained and tested using PICU data from the KidsBrainIT database (Lo et al., 2018) where it achieved a sensitivity of 81.0%, specificity of 88.4%, and AUCROC of 84.7% compared to the expert manual annotation likely with access to coincidently recorded ICP and HR signals	
Statistical modeling‐based (*n* = 1)	NIBP (*n* = 1)	Undisclosed (*n* = 1)	47	Lin et al. (2003) proposed a recursive weighted regression algorithm to smooth interference in NIBP recording when compared to manually recorded AuBP in healthy individuals and those with cardiovascular abnormalities. It achieved an R value of 0.98 and a standard error of estimation error of 4.7 mmHg	Directly tested (*n* = 1)
Signal quality‐based (*n* = 2)	NIBP (*n* = 1) NIBP/IBP (*n* = 1)	<1 Hz (*n* = 1) >1 Hz (*n* = 1)	28 (min = 4, max = 52)	Abdul Sukor et al. (2015) presented a signal quality analysis method that was able to correctly classify 75% of noisy signals. It was also able to identify the signals from which the systolic and diastolic points could be determined, achieving correct labeling in 97% and 91% of signals for systolic and diastolic BP, respectively. This performance was compared to labeling conducted using a coincidentally recorded ECG and AuBP signals Fanelli & Heldt (2014) presented a method capable of identifying noisy segments with accuracy of 99%, sensitivity of 95%, and specificity of 99% compared to expert annotations	Directly tested (*n* = 2)
Motion detection‐based (*n* = 1)	IBP (*n* = 1)	1 Hz (*n* = 1)	45	**Muroi et al. (** 2020 **) presented a motion sensor camera‐based method to label SBP and MAP alarms as valid or artifactual based on patient motion. SBP false alarms were detected with 38.4% sensitivity, 86.7% specificity, and 38.6% PPV. MAP false alarms at 35.6% sensitivity, 78.6% specificity, and 54.3% PPV. Signals were recorded in individuals suffering from various cerebral aneurysms and hemorrhages. This performance was compared to labeling conducted by a neurocritical care specialist without access to the motion sensor data, but may have had access to simultaneously recorded SpO**_**2**_, **ICP, and HR signals**	Directly tested (*n* = 1)
Articles presenting multiple/previously presented methods by new authors (*n* = 5)	NIBP/IBP (*n* = 2) IBP (*n* = 3)	<1 Hz (*n* = 4) Not specified (*n* = 1)	18 (min = 15, max = 106)	Maleczek et al. (2024) evaluate five artifact identification methods on IBP and NIBP recorded in OR and ICU. Leading results were varied across the five methods. For IBP, best sensitivity was from physiological cutoff method: 74.9%, best specificity from changepoint detection‐based Breunig et al. (2000) methodology: 100%, best PPV from physiological cutoff method: 99.7%, and best NPV from physiological cutoff method: 99.4%. For NIBP, best sensitivity was from IQR‐based method: 42.9%, best specificity from physiological cutoff method: 100%, best PPV from IQR‐based method: 9.4%, and best NPV from IQR‐based method: 99.9%. This performance was compared to labeling conducted by two experts with a third‐party to settle disagreements, they had access to simultaneously recorded HR, temperature, SpO_2_, and etCO_2_ signals Kuhrn et al. (2025) evaluated similar cut‐off‐based, Z‐score‐based, and IQR‐based methods. For IBP, the best sensitivity was from the Z‐score and IQR‐based methods: 41% and the best precision was that of the cut‐off: 100%. For NIBP, the best sensitivity was from the Z‐score method: 31%, all methods demonstrated poor precision: <10%. This was compared to manual review, the reviewer may have had access to simultaneously recorded oxygen saturation, etCO_2_, temperature, and pulse signals	Train/test for ML algorithm only (*n* = 1) Cross‐validation (*n* = 1) Directly tested (*n* = 3)
				Verduijn et al. (2008) evaluated a median filtering method, the ArtiDetect method proposed by Cao et al. (1999) and the multiple signal integration by tree induction method proposed by Tsien et al. (2000) applied to post‐operation cardiac surgery patients. Joint consensus validation between manual reviewers indicating the ArtiDetect method performed the best using a 10‐fold cross‐validation achieving an average sensitivity of 0.77 and PPV of 0.64 Khan et al. (2022) applied an optimized version of the Du et al. (2019) methodology on data recorded from critically ill patients. Using the optimal multiplication factor and optimized thresholds, sensitivity was 96.64% and specificity was 98.72% compared to annotations by a trained researcher Cretu et al. (2024) applied an asymmetric least squares method (Eilers & Boelens, 2005) and a discrete wavelet transformation‐based method (Hao et al., 2011) for the removal of baseline wander from IBP signals, with the wavelet method achieving a higher reduction in SNR of 27% compared to 6%. This study was conducted on post‐cardiac surgery patients with temporary pacemakers	

*Note*: *Descriptions of the validation methods used*: Directly tested = model was developed without training and was directly applied to a testing dataset; Train/test/validation = datasets were split into training, testing, and validation sets; Train/parameter tuning/test = models were trained, parameters tuned using the evaluation set, and tested; Training with cross‐validation/test = model was trained with concurrent k‐fold cross‐validation and tested; Train/test = datasets split into training and testing sets; Train/test by patient = datasets split into training and testing sets on a patient basis; Train/test for ML algorithm only = the only method that was not “directly tested” was the machine learning‐based algorithm which was trained and tested; Bolded text indicates that the method used a concurrently recorded auxiliary signal for artifact management.

Abbreviations: ABP, arterial blood pressure; AuBP, auscultatory blood pressure; AUCROC, area under the receiver operating characteristic curve; BP, blood pressure; DBP, diastolic blood pressure; ECG, electrocardiogram; etCO_2_, end‐tidal carbon dioxide; FP, false positive; HF, high frequency; IBP, invasive blood pressure; ICU, intensive care unit; IF, isolation forest; IQR, interquartile range; LF, low frequency; MAP, mean arterial pressure; ML, machine learning; NIBP, non‐invasive blood pressure; NPV, negative predictive value; OR, operating room; pCO_2_, partial pressure of carbon dioxide; PICU, pediatric intensive care unit; pO_2_, partial pressure of oxygen; PPV, positive predictive value; R, Pearson correlation coefficient; RR, reparatory rate; SBP, systolic blood pressure; SD, standard deviation; SDU, step‐down unit; SNR, signal‐to‐noise ratio; SpO_2_, pulse oximetry; SVM, support vector machine; XGBoost, extreme gradient boosting; β‐VAE, variational autoencoder with β hyperparameter.

There were 17 algorithms that were developed/used to detect artifacts for signals below 1 Hz (Abdul Sukor et al., 2015; Cao et al., 1999; Cao et al., 2006; Clark et al., 1987; Cunningham et al., 1994; Du et al., 2019; Haule et al., 2025; Hravnak et al., 2016; Imhoff et al., 1998; Khan et al., 2022; Kuhrn et al., 2025; Maleczek et al., 2024; Pasma et al., 2021; Tsien et al., 2000; Tsien et al., 2001; Verduijn et al., 2008; Wu et al., 2020). Seven of the presented algorithms were developed for higher frequency BP signals (≥1 Hz) that did not encapsulate the full waveforms (Deegan et al., 2007; Fanelli & Heldt, 2014; Keselbrener & Akselrod, 1995; Muroi et al., 2020; Sebald, 1989; Tronstad et al., 2015; Wilson et al., 2015). Two algorithms did not directly present a sampling rate (Cretu et al., 2024; Lin et al., 2003). The algorithms developed for artifact detection of low‐frequency BP signals can be categorized as threshold‐based methods (Cao et al., 1999; Cao et al., 2006; Clark et al., 1987; Cunningham et al., 1994; Deegan et al., 2007; Du et al., 2019; Keselbrener & Akselrod, 1995; Tronstad et al., 2015; Wilson et al., 2015), machine learning‐based methods (Haule et al., 2025; Hravnak et al., 2016; Pasma et al., 2021; Sebald, 1989; Tsien et al., 2000; Tsien et al., 2001; Wu et al., 2020), statistical modeling‐based methods (Imhoff et al., 1998; Lin et al., 2003), signal morphology‐based methods (Abdul Sukor et al., 2015; Fanelli & Heldt, 2014), and a motion detection‐based method (Muroi et al., 2020).

#### 3.1.1 Thresholding‐based methods

Algorithms classified as threshold‐based relied on methodologies that detected artifacts using amplitude or statistical deviation. Of these methods, three of them were developed using data collected from a non‐invasive finger cuff‐based Finapres device (Deegan et al., 2007; Keselbrener & Akselrod, 1995; Wilson et al., 2015), two used an invasive arterial line (Cao et al., 2006; Du et al., 2019), and Tronstad et al. (2015) used both a LiDCO invasive and a NexFin non‐invasive methods. Three articles did not specify how the data were recorded (Cao et al., 1999; Clark et al., 1987; Cunningham et al., 1994). The Cao et al. (1999) method was the only one reliant on an auxiliary signal (heart rate [HR]). Deegan et al. appeared to present particularly promising results, achieving the highest sensitivity and a low false positive rate (FPR) (Deegan et al., 2007). However, this effectiveness was undermined by Tronstad et al. (2015), who indicated that their algorithm outperformed that of Deegan et al. despite presenting relatively modest results for both precision and recall for artifact identification in IBP and NIBP signals. Potential reasons for this discrepancy include the small dataset (*n* = 20 subjects) and the single recording methodology on which the Deegan et al. method was tested (Deegan et al., 2007). The Du et al. algorithm also contributed a reasonably high sensitivity with a high specificity (Du et al., 2019). These values were further improved through optimization by Khan et al. (2022). Cao et al. (1999) also indicated strong results. Four of the methods included in this sub‐category performed post‐processing proceeding artifact identification to interpolate artifactual points. In instances where sufficient data was available, the methodologies presented by Keselbrener and Akselrod (1995), Cao et al. Cao et al. (1999), Du et al. (2019), and Deegan et al. (2007) used adjacent data points/pulses as the basis for interpolation using simple methodologies such as linear interpolation. The other methods presented in this sub‐section only identified or outright rejected the identified artifactual data points (Cao et al., 2006; Clark et al., 1987; Cunningham et al., 1994; Tronstad et al., 2015; Wilson et al., 2015).

#### 3.1.2 Machine learning‐based methods

There was a breadth of different machine learning algorithms which made them difficult to directly compare. Additionally, no information was provided regarding recording methodologies used; only Sebald (1989) specified the use of a catheter‐based device. Only Wu et al. (2020) used both NIBP and IBP recording methodologies. Tsien et al. (2000); Tsien et al. (2001) were the only two methods that used auxiliary signals (ECG, HR and partial pressure of CO_2_ and O_2_). The algorithms identified as having the highest effectiveness included the Extreme Gradient Boosting model (XGBoost) (Chen & Guestrin, 2016; Wu et al., 2020), the C4.5 decision tree induction model (Quinlan, 1993; Tsien et al., 2000; Tsien et al., 2001), back propagation topology (Sebald, 1989), and support vector machine (SVM) (Hravnak et al., 2016; Pasma et al., 2021). It should be noted that the SVM model was identified as a leading model in two studies. SVM (with no specified kernel) outperformed k‐nearest neighbor (KNN) at varying k values, linear discriminant analysis (LDA), naïve Bayesian classifier (NB), logistic regression (LR), and random forest (RF) models in artifact identification in the work by Hravnak et al. (2016). SVM with a radial basis function outperformed lasso penalized logistic regression and single layer neural network models in the work by Pasma et al. (2021). Wu et al. reported particularly strong results with the application of the XGBoost algorithm to both univariate and multivariate datasets (Wu et al., 2020). Tsien et al. (2001) reported strong results for their developed algorithm across sampling rates of 0.017 Hz and 1 Hz. Haule et al. (Haule et al., 2025) proposed a variational autoencoder with an adjustable hyperparameter combined with an independent forest algorithm (β‐VAE/IF) that was fully unsupervised, which compared relatively well by metrics of sensitivity and area under the receiver operator characteristic curve (AUCROC) to methods leveraging a long short‐term memory (LSTM) method, autoregressive integrated moving average (ARIMA) modeling, and an XGBoost method (Haule et al., 2025). It did not perform as well as these models in terms of sensitivity; however, it should be noted that no method presented overwhelmingly strong results for sensitivity (Haule et al., 2025). Real‐time application was seldom mentioned with the exception of Sebald (1989) who indicated that real‐time application was likely not feasible. None of the methods included in this section performed any post‐processing following artifact identification to interpolate artifactual segments.

#### 3.1.3 Statistical modeling‐based methods

The only algorithm described as statistical modeling‐based methods models the BP signal using a recursive weighted regression algorithm driven by fuzzy logic as a means of detecting artifacts using a custom oscillometric system (Lin et al., 2003). This method was indicated to have been designed for real‐time use, but there was no quantification provided. The post‐processing in this method involves the use of a recursive weighted regression algorithm to smooth the irregularities from the signal.

#### 3.1.4 Signal quality‐based methods

Signal quality‐based methods use either waveform quality assessed through comparison to a series of previous beats (Fanelli & Heldt, 2014) or noise classification based on signal morphology (Abdul Sukor et al., 2015). For data collection, Abdul Sukor et al. (2015) leveraged a self‐administered cuff‐based NIBP device. Fanelli and Heldt (2014) utilized both an invasive radial artery catheter and a finger‐cuff Finapres device. Fanelli and Heldt (2014) indicated particularly high accuracy, sensitivity, and specificity across artifact identification methods presented for low‐frequency ABP data. There was no post‐processing provided to interpolate the missing segments in the Abdul Sukor et al. article (Abdul Sukor et al., 2015). Fanelli and Heldt (2014) use an interpolation method to reconstruct the signal using. Using a 60 second window, erroneous beats are replaced with a template generated based on clean beats and scaled based on the amplitude of the surrounding signal. Neither of these articles mention real‐time utility of the proposed methods.

#### 3.1.5 Motion detection‐based methods

The motion detection‐based algorithm presented by Muroi et al. (2020) leveraged motion sensor cameras and an optical flow algorithm to delineate false alarms from true alarms in invasively recorded BP signals due to motion. This method only achieved modest results for sensitivity and specificity. Again, there was no mention of the real‐time applicability of any of these methods. No further post‐processing was conducted on the identified false alarms.

#### 3.1.6 Articles presenting multiple/previously presented methods by new authors

Maleczek et al. compared five different methods: amplitude thresholding based on physiological values, thresholding based on deviations from a calculated z‐value, thresholding based on interquartile range (IQR), a detection algorithm proposed by Breunig et al. to detect rapid changes in the data structure, and an LSTM machine learning model (Maleczek et al., 2024). Artifact detection in both IBP and NIBP was conducted across 53 operating room (OR) patients and 53 ICU patients. There remained an inability to select a leading method, as different methods were more advantageous based on different metrics including sensitivity, specificity, positive predictive value (PPV), and negative predictive value (NPV). Within each of those metrics, there were differences between the successfulness of each method in cuff‐based NIBP and arterial line IBP signals as well (Maleczek et al., 2024). This was further validated by Kuhrn et al. (2025), which compared two statistical deviation thresholding methods (IQR‐based and Z‐score‐based) and a physiologic threshold‐based method. These methods performed generally poorly across metrics of sensitivity and precision. Khan et al. (2022); Verduijn et al. (2008) both apply methods that had already been developed to novel datasets. Cretu et al. (2024) presented two methods for noise reduction in ABP signals using signal reconstruction using asymmetric least squares smoothing and a discrete wavelet transformation‐based method. However, noise reduction was not the main focus of the article, and signal‐to‐noise ratio was the only metric for effectiveness, which lacked a gold standard for comparison (Cretu et al., 2024). There was no mention of real‐time utility in any of these articles. Khan et al. (2022) was the only article in this sub‐category that presented a methodology for post‐processing after artifact identification. This interpolation involved consideration of simultaneously recorded MAP, SBP, and DBP as well as signal recorded immediately before and after the artifact (Khan et al., 2022).

Generally, many of the results reported by each of these articles must be scrutinized before any general conclusions can be made. The presented algorithms have high effectiveness; however, the differences in the populations, sampling methodologies/technologies, sampling rates, and metrics for evaluating effectiveness made it impossible to draw conclusions on a meta‐analytical basis. As a result, in the absence of larger datasets or multiple methods being compared internally, it remains difficult to discern a leading method or method type for this data.

### 3.2 Artifact management in full waveform ABP signals

There were 21 articles identified that presented methods for artifact detection and/or removal in full‐waveform ABP signals, possessing data sampling rates ≥100 Hz. A summary of the function and methodology of each algorithm, as well as detailed descriptions and additional results are included in the File S4, Table SD.1–SD.4. Also included is the pertinent information regarding the signal type, population size, sampling rate, and auxiliary signals recordings required for each method. The effectiveness of the developed algorithm on data collected on specific populations (with pathophysiology listed, if relevant) are listed in Table 2.

**TABLE 2:** Summary of artifact management methods in full‐waveform ABP signals.

Method type	Signals used	Sampling rate	Median population size	Effectiveness on population/dataset	Validation methods used	Real‐time utility
Machine learning‐based (*n* = 8)	IBP (*n* = 7) NIBP (*n* = 1)	100 Hz (*n* = 2) >100 Hz (*n* = 1) 125 Hz (*n* = 1) 200 Hz (*n* = 1) Unspecified, but full‐ waveform based on article information (*n* = 2)	48 (min = 1, max = 309)	Kim et al. (2018) presented a sensitivity of 96.3% and a specificity of 95.4% for their developed 6‐layer DBN on the validation set of severe TBI patients compared to expert annotation Lee et al. (2020) presented a stacked convolutional autoencoder (SCAE) which was combined with a convolutional neural network (CNN) a net prediction rate of 97.0% for artifacts in data sampled from TBI patients compared to two experts' annotations **Howard et al. (** 2019 **) presented a CNN to be used to classify BP signals segmented using ECG. It reported a 99.4% accuracy against internal reviewers and 98.7% accuracy against external reviewers when asked to classify beats extracted from invasive coronary angiography patients compared to a testing set classified by an external lab** Son et al. (2018) reported that that their developed DBN with a training dataset that encapsulated 20% of the collected data had a sensitivity of 96.34% and specificity of 95.42% compared to classifications by two experts working independently Pike and Mustard (1992) presented an FPR of 0.0466 and FNR of 0.0415 on its testing dataset composed of ICU patients for their signal morphology‐based ML classification algorithm compared to manual labeling with access to patient information Cabeleira et al. (2021) presented their SAX string algorithm that used signal morphologic characteristics labeled using an SVM model with no specified kernel for classification. It was compared to two independent classifiers, yielding sensitivities of 97.2% and 95.4% and specificity of 87.7% and 87.7% compared to annotations by two independent researchers Edinburgh et al. (2019) reported an accuracy of 90.1%, sensitivity of 91.9%, specificity of 86.8%, and AUCROC of 97.3% on ICU patients with the classification conducted by their variational autoencoder‐based method compared to annotations by two experts	Train/test by patient (*n* = 2) Training with cross‐validation (*n* = 1) Train/validate model performance or parameters/test (*n* = 2) Train/test (*n* = 2) Multiple validation techniques used (*n* = 1)	No mention in article (*n* = 3) Possible (*n* = 4) Indicated to be possible but no quantification (*n* = 1)
				Ouyoung et al. (2022) presented eight machine learning methods. Through a three‐fold cross‐validation, the RF model achieved a mean accuracy of 85.36, sensitivity of 88%, specificity of 85%. However, there were similarities in terms of the performance between methods compared to manual annotations by two experts		
Wavelet transformation‐based (*n* = 3)	NIBP (*n* = 1) TCPD (*n* = 2)	100 Hz (*n* = 1) 250 Hz (*n* = 1) Not specified, but images suggest full waveform (*n* = 1)	104 Not specified (*n* = 2)	Nguyen et al. (2015) reported an improved accuracy of detecting DBP and SBP within 5 mmHg following the application of the wavelet‐based denoising algorithm going from 0% to 75% for SBP and from 50% to 100% in DBP with healthy individuals told to move their arm. The “gold‐standard” BP measurements were administered manually Xu et al. (2005) reported that their wavelet‐based baseline drift removal resulted in an increase in human identification by multiple human experts of pulses from 67% to 83% This same statistic was also reported in Xu et al. (2007), which was presented as a refinement of the previous algorithm	Directly tested (*n* = 3)	No mention in article (*n* = 3)
Motion detection‐based (*n* = 1)	NIBP (*n* = 1)	2000 Hz (*n* = 1)	5	**Abderahman et al. (** 2017 **) proposed a method that used an accelerometer and ECG signals which was able to reduce vibrational artifacts such that the MAE in SBP and DBP estimation went from 3.1 to 0.8 mmHg and 3.5 to 1.1 mmHg, respectively. Transient motion artifacts were suppressed such that MAE in SBP and DBP estimation went from 9.4 to 2.4 mmHg and 5.3 to 2.4 mmHg, respectively. These were compared to a conventional oscillometric recording method**	Directly tested (*n* = 1)	Not possible (*n* = 1)
Signal quality‐based (*n* = 9)	IBP (*n* = 4) NIBP (*n* = 3) NIBP/AuBP (*n* = 1) Non‐specific ABP (*n* = 1)	100 Hz (*n* = 2) 125 Hz (*n* = 4) 1000 Hz (*n* = 3)	Human: 36.5 (min = 2, max = 120) Animal: 9 Not specified (*n* = 2)	Sun et al. (2006) reported a PPV of 73%, NPV of 100%, sensitivity of 100%, and specificity of 91% in binary classification of artifactual segments in data recorded from ICU patients compared to human expert classification using simultaneously recorded CBFV **Zong et al. (** 2004 **) presented a method using an ECG signal that reported a false alarm rejection rate of 98.2% while only rejecting 0.2% of real alarms in data recorded from ICU patients compared to the classifications made by the authors using a simultaneously recorded ECG** Abdul Sukor et al. (2012) presented a method to classify noise and motion which reported average accuracy, sensitivity, and specificity values between all signals of 97.06% ± 4.04%, 75.92% ± 28.63%, and 98.18% ± 3.83%, respectively, compared to human annotations with access to a simultaneously recorded ECG Zhang et al. (2010) promoted a feature‐based artifact detection algorithm to achieve a sensibility of 93.95% and specificity of 94.47% when the Sun et al. (2006) SAI method was implemented with end‐diastole slope sum as an added feature for the algorithm to consider. These results were compared to annotations by two experts that had access to a CBFV signal **Ali et al. (** 2004 **) presented a method using a simultaneously recorded ECG that indicated a sensitivity of 90% and specificity of 99% in false alarm identification from ICU patients compared to physician annotation with access to a simultaneously recorded ECG signal** **AIi and Eshelman (** 2004 **) presented a method using a simultaneously recorded ECG signal which indicated that the developed algorithm was able to correctly classify 98% of true alarms but only 45% of false alarms in ICU patient monitoring compared to manual annotations by an external expert**	Directly tested (*n* = 6) Training with cross‐validation (*n* = 1) Train/test (*n* = 2)	No mention in article (*n* = 6) Indicated to be possible but no quantification (*n* = 3)
				Nagai and Nagata (1995) presented an algorithm capable of rejecting 100% of movement artifacts and 191 of 215 of arrythmia beats with those unrejected being due to bradycardia arrythmias. Details regarding the annotations against which this result was compared were not provided Charbonnier et al. (2000) reported that valid and artifactual pulses was validated on two healthy individuals, between the two patients average correct classification, sensibility, and specificity were 75%, 85.5%, and 69.5% compared to annotations by a cardiologist **Lim et al. (** 2015 **) indicated that the identification and replacement of erroneous BP measurements estimated using a multiple linear regression model and aided using a simultaneously recorded ECG signal resulted in a mean change in SBP error of − 0.3 ± 5.8 mmHg and using a support vector regression − 0.6 ± 5.4 mmHg, with little improvement in DBP error. However, many other models using different features were also evaluated. The error in the BP measurements were relative to manual measurement using AuBP**		

*Note*: *Descriptions of the validation methods used*: Train/test by patient = datasets divided into training and testing on a patient basis; Training with cross‐validation = model was trained with concurrent k‐fold cross‐validation; Train/validate model or parameters/test = models were trained, the relative performance of the different model designs or their parameters was assessed, and the final model was tested; Train/test = dataset was split into training and testing sets; Multiple validation techniques used = techniques included k‐fold cross‐validation, leave‐one‐subject‐out validation, and hold‐out validation; Directly tested = model was developed without training and was directly applied to a testing dataset. Bolded text indicates that the method used a concurrently recorded auxiliary signal for artifact management.

Abbreviations: ABP, arterial blood pressure; AuBP, auscultatory blood pressure; AUCROC, area under the receiver operating characteristic curve; BP, blood pressure; CBFV, cerebral blood flow velocity; CNN, convolutional neural network; DBN, deep belief network; DBP, diastolic blood pressure; ECG, electrocardiogram; FNR, false negative rate; FPR, false positive rate; HR, heart rate; Hz, Hertz; IBP, invasive blood pressure; ICU, intensive care unit; MAE, mean absolute error; ML, machine learning; mmHg, millimeters of mercury; NIBP, non‐invasive blood pressure; NPV, negative predictive value; PPV, positive predictive value; RF, random forest; SAI, signal abnormality index; SBP, systolic blood pressure; SCAE, stacked convolutional autoencoder; SpO_2_, pulse oximetry; SVM, support vector machine; TBI, traumatic brain injury; TCPD, traditional chinese pulse diagnosis; VAE, variational autoencoder.

Eight algorithms leveraged machine learning techniques for artifact detection (Cabeleira et al., 2021; Edinburgh et al., 2019; Howard et al., 2019; Kim et al., 2018; Lee et al., 2020; Ouyoung et al., 2022; Pike & Mustard, 1992; Son et al., 2018). Two of these algorithms indicated that the detected artifacts would be removed, but provided no insights as to how the missing values would be interpolated (Kim et al., 2018; Son et al., 2018). Four algorithms capable of identifying artifacts using a wavelet transformation methodology were identified (Nguyen et al., 2015; Xu et al., 2005; Xu et al., 2007). There was only a single method developed for artifact identification in full‐waveform BP signals that leveraged motion detection using an accelerometer (Abderahman et al., 2017). Nine artifact detection and/or removal algorithms used a developed signal quality index (Abdul Sukor et al., 2012; AIi & Eshelman, 2004; Ali et al., 2004; Charbonnier et al., 2000; Lim et al., 2015; Nagai & Nagata, 1995; Sun et al., 2006; Zhang et al., 2010; Zong et al., 2004). Again, a majority of models were designed solely to identify artifacts; however, some included artifact removal information. These methodologies included artifact suppression (Abderahman et al., 2017; Nguyen et al., 2015), reduction in baseline wander (Xu et al., 2005; Xu et al., 2007), and reconstruction using developed machine learning models (Edinburgh et al., 2019; Lim et al., 2015).

#### 3.2.1 Machine learning‐based methods

The machine learning models for this application were diverse but had recurring themes in some of the methodologies used. The method presented by Ouyoung et al. (2022) utilized an NIBP signal which involved holding a pressure transducer to the radial artery. The remainder of the machine learning models was developed using IBP signals. Howard et al. (2019) presented the only method that used an auxiliary signal (ECG). Both Lee et al. (2020) and Howard et al. (2019) presented convolutional neural network (CNN)‐based image classification algorithms for artifact labeling. The Howard et al. algorithm had an additional complexity as it was able to label beats as “normal,” “noisy non‐dampened,” “port open” (of catheter), and “damped,” among others, whereas the Lee et al. (2020) algorithm solely performed binary classification of “valid” and “invalid” pulses. Edinburgh et al. used a variational autoencoder (VAE), also constructed using CNNs, to estimate the marginal likelihood of the data by integrating over latent variables using variational Bayesian inference to classify ABP waveforms (Edinburgh et al., 2019). Both Son et al. (2018) and Kim et al. (2018) proposed the use of deep belief networks (DBN) for the classification of segmented ABP pulses, trained using human annotation. Pike and Mustard (1992) and Cabeleira et al. (2021) developed algorithms to classify signal morphologies for artifacts. Ouyoung et al. (2022) evaluated several machine learning methodologies for the classification of pulses as high or low quality based on several harmonic indices. It was ultimately concluded that the random forest (RF) algorithm was the most reliable of the eight machine learning models tested (SVM, multilayer perceptron (MLP), Gaussian Naïve Bayes (GNB), decision tree (DT), RF, logistic regression (LR), linear discriminant analysis (LDA), and KNN) (Ouyoung et al., 2022). There were some similarities in the population types used to test these machine learning‐based methods. There were four machine learning algorithms developed and validated using traumatic brain injury (TBI) data (Cabeleira et al., 2021; Kim et al., 2018; Lee et al., 2020; Son et al., 2018). However, despite each of these methods being validated using a train‐test split of datasets composed of 10, 30, 30, and 58 TBI patients for Lee et al. (2020), Son et al. (2018), Kim et al. (2018), and Cabeleira et al. (2021), respectively, they all achieved remarkably similar sensitivities of 97% ± 1%. This highlights the difficulty in disseminating a leading method even among models of similar types and populations. Howard et al. (2019) also presented tremendous accuracy, albeit on a different dataset. Multiple algorithms mentioned the real‐time applicability of these methods. Howard et al. (2019) indicated that a pulse could be processed in under 1 second, thus facilitating essentially real‐time use; however, Kim et al. (2018) indicated that there was essentially no latency in the processing for this method; a time lag of 10 seconds was sufficient for real‐time processing using the developed algorithm. Son et al. (2018) presented real‐time ratio data which indicated that the best‐performing DBN could easily function in real time. It was indicated by Howard et al. (2019) that their developed method could process a beat in under 1 second. Edinburgh et al. (2019) indicated that after training, the algorithm could process a waveform in the order of milliseconds. However, it should be noted that CPU requirements were not specified for these benchmarks. Cabeleira et al. (2021) indicated the potential for real‐time use but did not specify any time lag or testing conducted. It should also be acknowledged that none of these articles discussed the computational requirements to conduct artifact identification in real time. This is of significance as it may limit the ability of certain centers to achieve real‐time processing using these methods. In terms of post‐processing, several of the articles presented in this subsection simply reject artifactual data points (Cabeleira et al., 2021; Edinburgh et al., 2019; Kim et al., 2018; Lee et al., 2020; Pike & Mustard, 1992). Ouyoung et al. (2022) labeled segments as either high or low quality pulses. Howard et al. (2019) was the only one of these methods that differentiated between artifact types as mentioned earlier in this section.

#### 3.2.2 Wavelet transformation‐based methods

Of the wavelet‐based methods identified, only Rodriguez and Giraldo (2018) directly evaluated the effectiveness of classification of artifacts using non‐invasive finger‐cuff BP data; however, this article was not included in this review as the artifacts identified and removal were simulated. Nguyen et al. (2015) designed a method to smooth motion artifacts, which was quantified by ease of detection of fiducial points for data collected using a custom non‐invasive oscillometric BP monitor (Nguyen et al., 2015). Xu et al. (2005); Xu et al. (2007) presented methods for baseline drift removal which was quantified using signal quality for data collected using traditional Chinese pulse diagnosis‐based systems. The methods presented in this sub‐section did not conduct any post‐processing beyond artifact reduction.

#### 3.2.3 Motion detection‐based methods

The single motion detection‐based method also indicated improvements in error reduction (Abderahman et al., 2017). This method leveraged an accelerometer that was embedded inside an NIBP cuff (Abderahman et al., 2017). There was no mention of the real‐time application of this method. This method suppressed artifacts but did not conduct any additional post‐processing.

#### 3.2.4 Signal quality‐based methods

There were several signal quality‐based methods that had strong performances in artifact identification and classification. Sun et al. (2006) demonstrated high sensitivity and specificity for its signal abnormality index (SAI); however, Zhang et al. (2010) indicated that they had developed an improved version of the Sun et al. (2006) SAI algorithm which was able to achieve a sensibility of 93.95% and specificity of 84.47% by incorporating the end diastole slope sum as a feature. It outperformed the original which achieved sensibility and specificity values of 97.75% and 18.38%, respectively (Zhang et al., 2010). However, the method presented by Zong et al. (2004) was also validated on this dataset, achieving a sensibility of 97.45% and specificity of 82.77% (Zhang et al., 2010). As such, it seems as though there is a trade‐off between the Zhang et al. (2010) algorithm and the Zong et al. (2004) algorithm on the basis of sensibility and specificity. However, both methods demonstrate strong results. There was a wavelet‐based method for baseline noise artifact removal (Wang et al., 2003) and a short‐time Fourier transform‐based method for artifact identification that were identified; however, they were out of the scope of this review as they used simulated artifacts added to real ABP signals (Trukhan et al., 2024). Zhang et al. (2010) mention that the proposed algorithm can be used in real‐time, but provide no data to support this claim. An array of recording methodologies was used to collect the data used for the development of these methods. Four methods used catheter‐based invasive methods (AIi & Eshelman, 2004; Ali et al., 2004; Nagai & Nagata, 1995; Sun et al., 2005), one used a tonometry device (Zhang et al., 2010), two used custom‐built NIBP devices (Charbonnier et al., 2000; Lim et al., 2015), and one used a cuff‐based NIBP device (Abdul Sukor et al., 2012). Real‐time applicability was seldom mentioned; Nagai and Nagata indicated it was possible using their presented algorithm, but no evidence was provided (Nagai & Nagata, 1995). Those methods that used auxiliary signals included Zong et al. (2004), Ali et al. (2004), and AIi and Eshelman (2004) who leveraged ECG signals; Abdul Sukor et al. (2012) used two simultaneously recorded methods of NIBP recordings, and Zhang et al. (2010) used cerebral blood flow volume (CBFV) determined using transcranial Doppler (TCD). All of the articles presented in this sub‐section did not perform any additional post‐processing after artifact identification or simply rejected artifactual data points (Abdul Sukor et al., 2012; AIi & Eshelman, 2004; Ali et al., 2004; Charbonnier et al., 2000; Lim et al., 2015; Nagai & Nagata, 1995; Sun et al., 2006; Zhang et al., 2010; Zong et al., 2004).

Similar to the previous section, it remains difficult to draw conclusions regarding a single leading method due to the heterogeneity of the datasets used, both in size and in the presence of varying pathophysiology, as well as in the recording methodologies and data sampling rates.

### 3.3 Methodologies for the identification of valid or fiducial points in ABP signals

Critical to the calculation of derived metrics is the ability to assess the validity of ABP pulses. This can be accomplished by validating pulses directly or validating the temporal location of certain features or points in the ABP waveform. There were 29 articles identified that presented methodologies for the identification of valid pulses or the identification of fiducial features of the ABP waveform that could be used as a means for validating pulses. There were two broad categories of such algorithms: valid pulse identification algorithms and fiducial point algorithms. Neither of these algorithms directly detected artifacts within the BP signals. However, the ability to validate BP pulses or identify specific points present in waveforms that are not heavily contaminated with noise or artifacts can be logically extended to identify artifactual segments. It should be noted that it had appeared that the Kyle and Freis (1970) algorithm would be included in this review; however, it provided quantification of the effectiveness of the algorithm in both ECG and ABP‐based feature detection, and did not explicitly list the effectiveness for ABP alone.

Table 3 displays a summary of the articles describing algorithms designed for this purpose. These algorithms were organized by their respective function; however, also included was the patient population(s) used to develop the algorithm, the signal type used, the sampling rate(s) of the dataset, the population size, the validation technique used, and the applicability of this method in real time. Encapsulated within patient population information was the pathophysiology of the group (if available) as well as whether the data was publicly available. It should be noted that there are some algorithms that were designed to detect multiple fiducial points, allowing them to fall into multiple categories in Table 3. A summary of the function and methodology of each algorithm, as well as detailed descriptions and additional results, is included in the File S5, Tables SE.1–SE.5. Also included is the pertinent information regarding the signal type, population size, pathophysiology, sampling rate, and auxiliary signals recordings required for each method.

**TABLE 3:** Summary of identification of valid or fiducial points in ABP signals.

Method type	Signals used	Sampling rate	Median population size	Methodology and effectiveness on population/dataset	Validation methods used	Real‐time utility
Valid pulse detection (*n* = 4)	IBP (*n* = 3) ABP (*n* = 1)	>100 Hz (*n* = 3) Undisclosed (*n* = 1)	171 (min = 30, max = 210)	Aboy et al. (2005) presented a peak detection methodology using spectral analysis and rank‐based filtering for valid pulse detection using a nearest neighbor logic was able to achieve a sensitivity and PPV of 100% for 2179 pulses measured from patients with TBI, sepsis, and cardiac conditions from the PICU compared to manual annotations conducted by two experts with access to ICP and SpO_2_ signals **Asgari et al. (** 2010 **) presented a SVD‐based ABP pulse validation method that leveraged a simultaneously recorded ECG signal which was combined with the Sun et al. SAI method (Sun et al**., 2006 **) to achieve a PPV of 99.23% and NPV of 95.00% on pulses from the MIMIC‐II (Saeed et al**., 2002 **) dataset with a total of 1336 10‐second ABP segments** Hoeksel et al. (1996) presented a feature‐based method for validation of pulses which assigned 77% ± 11% (mean ± SD) of pulses as valid when there were 85% ± 13% truly valid pulses on data recorded from cardiac surgery patients identified by an experienced clinician Urteaga et al. (2024) presented a method that used the filtered first difference of the ABP signal to differentiate between pulsatile and non‐pulsatile data segments using physiological and adaptive thresholding on identified fiducial points. It was able to achieve results (mean ± SD) for sensitivity of 98.8% ± 6.9%, specificity of 91.6% ± 20.2%, PPV of 97.4 ± 9.7%, and NPV of 98.7 ± 6.1% compared to manual annotation conducted using a simultaneously recorded ECG signal. It outperformed two established algorithms in this application on 377 segments (Li et al., 2010; Zong et al., 2003)	Directly tested (*n* = 4)	No mention in article (*n* = 4)
Dicrotic notch detection (*n* = 12)	IBP (*n* = 6) Unspecified ABP (*n* = 2) NIBP (*n* = 1) Unspecified ABP/IBP (*n* = 1) Unspecified ABP/IBP/NIBP (*n* = 1)	≥100 Hz (*n* = 12)	Human populations: 25 (min = 3, max = 4901) Animal populations: 8.5 (min = 4, max = 14) Not specified (*n* = 2)	Pal et al. (2024) presented an iterative mean envelope‐based method to detect the dicrotic notch which was able to detect 100% of notches with an average detection error of 4.7 milliseconds compared to manual identification Oppenheim and Sittig (1995) developed an optimal detection method for the dicrotic notch using the bentpoint algorithm proposed by Kinias et al. (1981) and second derivative‐based methods (Nygårds et al., 1976; Starmer et al., 1973). It was able to achieve an accuracy of 96% with a mean error of 6.5 ± 8.8 milliseconds for all dicrotic notch and incisurae compared to manual identification Hoeksel et al. (1997) developed a model in which they modeled the blood pressure signal to arterial blood flow (initial model developed using canine data) and located the dicrotic notch using this modeled signal. They used a linear and a non‐linear model for modeling which were able to detect the DN with 98% and 96%, respectively, compared to manual identification by an experienced clinician Balmer et al. (2018) use an adaptive shear transformation to identify the DN, it had a systemic error in identification of 0.5 milliseconds, outperforming the Kamoi et al. (2017) algorithm which had error of 11.6 milliseconds. These were compared to human observer annotation Donelli et al. (2002) leveraged the Hoeksel et al. (1997) algorithm and extended it to datasets with varying flow in individuals undergoing cardiac surgery. The algorithm was able to detect 98.1% of DNs with no false identifications this was compared to human observers	Directly tested (*n* = 9) Train/test (*n* = 2) Train/validate/test/external validation (*n* = 1)	No mention in article (*n* = 9) Indicated to be possible but no quantification (*n* = 3)
				Saffarpour et al. (2023) present a DN identification model that applies rules‐based logic developed by Li et al. (2010) to an optimized cardiovascular model for detection. It was able to achieve an average detection error of 20 ± 28 milliseconds (mean ± SD) in data collected from sepsis and hemorrhage patients, with 82% of detections falling within an acceptable 30 milliseconds of error compared to the annotations of an experienced physician Singh and Sunkaria (2017) use a wavelet decomposition‐based method to identify the DN among other fiducial points. It was able to achieve a sensitivity of 98.98% and PPV of 98.81% compared to annotations conducted by a trained cardiologist, for some of the datasets, the individual would have had access to a simultaneously recorded ECG signal Kinias et al. (1981) used a bentpoint algorithm for identification of DNs. Effectiveness was measured with the time between ejection and DN, the mean (SD) error in detection for each of the coronary care patients were: 5.41% (14.10%), −6.66% (11.08%), −8.54% (16.05%) compared to human observer annotations **Pachauri and Bhuyan (** 2012 **) developed a wavelet‐based method for initial filtering and detection of DN. It was validated on MGH/MF (Goldberger et al**., 2000 **; Welch et al**., 1991 **), Fantasia (Goldberger et al**., 2000 **; Iyengar et al**., 1996 **), MIT‐BIH polysomnographic (Goldberger et al**., 2000 **; Ichimaru & Moody**, 1999 **), and CSL (Aboy et al**., 2005 **) datasets. The MGH/MF was the most generalizable as it had been used in other articles. The algorithm achieved an accuracy in DN detection of 98.54%, sensitivity of 99.35%, and PPV of 99.19% in the annotation of 1860 beats. The annotations were provided through the public datasets, two of the datasets used simultaneously recorded ECG signals for annotations**		
				Li et al. (2010) used derivative analysis to detect DNs in pulses from the CSL (Aboy et al., 2005), Fantasia (Goldberger et al., 2000), and SLP (Ichimaru & Moody, 1999) datasets. The SLP and Fantasia databases were annotated and presented effectiveness for different fiducial points, the DN detection using the algorithm achieved a sensitivity of 96.53% and PPV of 96.64% for 2564 pulses. The annotations were provided through the public datasets, two of the datasets used simultaneously recorded ECG signals for annotations Aguirre et al. (2021) implemented a U‐Net machine learning methodology and a U‐Net with an 8‐layer temporal convolutional network (U‐TCN). The MIMIC‐III dataset was used for training, testing, and validation. It was able to achieve sensitivities of 98.96% and 98.92%, respectively, and PPVs of 99.04% and 99.09% for DN detection in 64,155 pulses. The annotations were provided through the public datasets, two of the datasets used simultaneously recorded ECG signals for annotations Stevenson et al. (2012) proposed an algorithm that leveraged the shear transformation for feature extraction from blood pressure signals. Among other fiducial points, this algorithm was able to detect the dicrotic notch with within 1% accuracy for 87 of 88 waveforms measured from porcine subjects that had an induced pulmonary embolism (51 waveforms) in development set or septic shock (37 waveforms) in validation set. The annotations were provided through manual identification of features		
Pulse onset detection (*n* = 9)	NIBP (*n* = 1) NIBP/IBP (*n* = 1) IBP (*n* = 2) IBP/Non‐specific ABP (*n* = 1) IBP/NIBP/Non‐specific ABP (*n* = 2) Non‐specific ABP (*n* = 2)	>100 Hz (*n* = 8) Undisclosed (*n* = 1)	33 (min = 10, max =350) Not specified (*n* = 1)	Zong et al. (2003) proposed an adaptive filter method based on the slope sum function that had an average sensitivity and PPA of 99.26% and 99.77%, respectively, on IBP signals, and 99.71% and 99.72%, respectively, on NIBP signals compared to human expert annotations Singh and Sunkaria (2017) use a wavelet decomposition‐based method to identify the pulse onset among other fiducial points. It was able to achieve a sensitivity of 99.88% and PPV of 99.92% compared to human annotations, with some of the datasets possessing concurrently recorded ECG signals **Pachauri and Bhuyan (** 2012 **) developed a wavelet‐based method for initial filtering as well as detection of pulse onsets. It was validated on MGH/MF (Goldberger et al**., 2000 **; Welch et al**., 1991 **), Fantasia (Goldberger et al**., 2000 **; Iyengar et al**., 1996 **), MIT‐BIH polysomnographic (Goldberger et al.,** 2000 **; Ichimaru & Moody**, 1999 **), and CSL (Aboy et al**., 2005 **) datasets. The MGH/MF was the most generalizable as it had the greatest number of signals. The algorithm achieved an accuracy in pulse onset detection of 97.96%, sensitivity of 99.62%, and PPV of 98.36% in the annotation of 1868 beats. The annotations were provided through the public datasets, two of the datasets used simultaneously recorded ECG signals for annotations**	Directly tested (*n* = 8) Train/validate/test/external validation (*n* = 1)	No mention in article (*n* = 9)
				Li et al. (2010) used derivative analysis to detect pulse onset in pulses from the CSL (Aboy et al., 2005), Fantasia (Goldberger et al., 2000), and SLP (Ichimaru & Moody, 1999) datasets. The SLP and Fantasia were annotated and displayed effectiveness for fiducial point detection including pulse onsets. This algorithm achieved a sensitivity of 99.96% and PPV of 98.73% for 2564 pulses. The annotations were provided through the public datasets, two of the datasets used simultaneously recorded ECG signals for annotations Aguirre et al. (2021) implemented a U‐Net machine learning methodology and a U‐Net with an 8‐layer temporal convolutional network (U‐TCN). The MIMIC‐III dataset (Johnson et al., 2016) was used for training, testing, and validation. The U‐TCN and U‐Net models achieving specificities of 99.69% and 99.78%, respectively, and PPVs of 99.78% and 99.78%, respectively, in 64,133 pulses from ICU patients. The annotations were provided through the public datasets, two of the datasets used simultaneously recorded ECG signals for annotations Xu et al. (2009) proposed an algorithm to enhance signal quality and detect the pulse onset using three different methodologies. The diastolic point‐based method that used ECG‐based annotation and cubic spline fitting had the best results with the error in identification despite noise being added was 1.15 ± 6.02 milliseconds for data collected from patients with SAH and NPH Kyle et al. presented methodology for the detection of fiducial points including the pulse onset. Correlation between the manual observer and automated method was calculated for time between located pulse onsets, the algorithm achieved an R value of 0.99 for healthy patients and those with cardiovascular conditions compared to annotations by a trained physician (Kyle et al., 1968)		
				Singh and Sunkaria (2016) developed a wavelet‐based method for the detection of the pulse onset and systolic peaks. The pulse onset detection for data segments from the MIT‐BIH (Goldberger et al., 2000; Ichimaru & Moody, 1999), Fantasia (Goldberger et al., 2000; Iyengar et al., 1996), and MIMIC (Goldberger et al., 2000; Moody & Mark, 1996) databases achieved sensitivities of 99.92%, 100%, and 99.93% and PPVs of 99.92%, 99.97%, and 99.87%. The annotations used were provided by the public datasets Lee et al. (2018) developed a method that used peak detection based on windowing and the signal gradient. It was tested using the MIMIC database (Goldberger et al., 2000) with 22,544 pulses (approximately 10% artifacts). Average mean error of pulse onset detection was approximately 2.4 microseconds ±12.5 microseconds (Lee et al., 2018). The annotations used were provided by the public datasets		
Systolic peak detection (*n* = 9)	NIBP (*n* = 1) IBP (*n* = 2) IBP/Non‐specific ABP (*n* = 1) IBP/NIBP/Non‐specific ABP (*n* = 2) Non‐specific ABP (*n* = 3)	>100 Hz (*n* = 9)	38 (min = 3, max = 350) Not specified (*n* = 2)	Singh and Sunkaria (2017) use a wavelet decomposition‐based method to identify the systolic peak among other fiducial points. It was able to achieve a sensitivity of 99.95% and PPV of 99.97% compared to human annotations, with some of the datasets possessing concurrently recorded ECG signals Kinias et al. (1981) used a bentpoint algorithm for identification of the systolic peak. Effectiveness was measured using the time between systolic peaks, the mean (SD) error in detection for each of the coronary care patients were: 1.53% (3.34%), 1.80% (6.65%), 0.83% (3.72%) compared to human annotations	Directly tested (*n* = 8) Train/external validation (*n* = 1)	No mention in article (*n* = 7) Possible but no validation (*n* = 2)
				**Pachauri and Bhuyan (** 2012 **) developed a wavelet‐based method for initial filtering as well as detection of pulse onsets. It was validated on MGH/MF (Goldberger et al**., 2000 **; Welch et al**., 1991 **), Fantasia (Goldberger et al**., 2000 **; Iyengar et al**., 1996 **), MIT‐BIH Polysomnographic (Goldberger et al**., 2000 **; Ichimaru & Moody**, 1999 **), and CSL (Aboy et al**., 2005 **) datasets. The MGH/MF was the most generalizable as it had the greatest number of signals. The algorithm achieved an accuracy systolic peak detection of 97.27%, sensitivity of 99.52%, and PPV of 97.79% in the annotation of 1873 beats. The annotations were provided through the public datasets, two of the datasets used simultaneously recorded ECG signals for annotations** Li et al. (2010) used derivative analysis to detect systolic peaks in pulses from the CSL (Aboy et al., 2005), Fantasia (Goldberger et al., 2000), and SLP (Ichimaru & Moody, 1999) datasets. The SLP and Fantasia databases were manually annotated and displayed effectiveness for fiducial point detection, the detection of systolic peaks using the algorithm achieved a sensitivity of 99.88% and PPV of 98.69% for 2564 pulses. The annotations were provided through the public datasets, two of the datasets used simultaneously recorded ECG signals for annotations Aguirre et al. (2021) implemented a U‐Net machine learning methodology and a U‐Net with an 8‐layer temporal convolutional network (U‐TCN). The MIMIC‐III dataset was used for training, testing, and validation. It was able to achieve high sensitives of 99.48% and 99.50%, respectively, with similarly high PPV results of 99.72% and 99.82%, respectively. The annotations were provided through the public datasets, two of the datasets used simultaneously recorded ECG signals for annotations		
				Kyle et al. (1968) presented methodology for the detection of fiducial points including the systolic peak. Correlation between the manual observer and automated method was calculated for time between located pulse onsets and systolic peak, the algorithm achieved an R value of 0.97 for healthy patients and those with cardiovascular conditions Singh and Sunkaria (2016) developed a wavelet‐based method for the detection of the pulse onset and systolic peaks. The peak detection for segments from MIT‐BIH (Goldberger et al., 2000; Ichimaru & Moody, 1999), Fantasia (Goldberger et al., 2000; Iyengar et al., 1996), and MIMIC (Goldberger et al., 2000; Moody & Mark, 1996) databases achieved sensitivities of 99.96%, 99.97%, and 99.95% and PPVs of 99.88%, 99.97%, and 99.91%. The annotations used were provided by the public datasets Pachauri and Bhuyan (2011) developed a method for systolic pressure detection using signal energy. It was validated using the mgh001 dataset (Goldberger et al., 2000; Welch et al., 1991). The algorithm was able to achieve an accuracy of 99.53% across pulses. The annotations were provided by the public datasets with ECG used for annotations Nygårds et al. (1976) presented a method to detect several morphological features. It was able to detect the systolic blood pressure with average difference ± SD from manual observation of 1.3 ± 3.8 mmHg. The annotations were provided by a trained physician with access to ECG, pulmonary artery pressure, right atrial pressure, and left ventricular pressure		
Other fiducial point detection (*n* = 5)	Non‐specific ABP (*n* = 3) NIBP (*n* = 1) IBP (*n* = 1)	>100 Hz (*n* = 5)	Human: 110 (min = 8, max = 2000) Animal: 4	**Swatzell et al. (** 1973 **) designed an algorithm using an ECG signal capable of using components of the waveform morphology to detect the systolic time. It was able to achieve an identification accuracy of 95% compared to manual annotation** Almeida et al. (2011) designed an algorithm to detect the systolic peak, reflection peak, dicrotic peak, and dicrotic notch using derivative analysis and morphological features. For the detection of these points, the temporal average error, sensitivity, and PPV were 4.20%, 99.09%, and 96.77%, respectively. The amplitude average error, sensitivity, and PPV were 2.68%, 99.08%, and 98.22%, respectively, compared to a human expert engineer Paradkar and Chowdhury (2015) developed a method using SVD for artifact suppression and used a wavelet‐based method to detect the systolic peak, percussion peak, tidal peak, and diastolic peak. The MIMIC‐II database was used to train the algorithm (Saeed et al., 2011), it was validated using the CSL dataset (Aboy et al., 2005). For the validation set it achieved a sensitivity of 99.24% and a PPV of 98.48%. Annotations were provided through the public datasets Prakash et al. (2008) developed a wavelet‐based method for detection of the percussion wave, tidal wave, and dicrotic notch. It achieved an overall success rate of 97.89% on healthy individuals compared to human pulse identification where the human likely had access to a simultaneously recorded PPG signal	Directly tested (*n* = 4) Train/external validation (*n* = 1)	No mention in article (*n* = 5)
				**Starmer et al. (** 1973 **) used a simultaneously recorded ECG signal to segment the ABP signal such that derivative analysis could be used to detect the ejection onset and ejection end. Ejection onset and ejection end were able to be identified compared to manual annotation within be 9 ± 1 milliseconds and −7 ± 1 milliseconds, respectively**		

*Note*: *Descriptions of the validation methods used*: Train/validate model or parameters/test/external validation = models were trained, the relative performance of the different model designs or their parameters was assessed; the final model was tested and then externally validated using another dataset; Train/test = dataset was split into training and testing sets; Directly tested = model was developed without training and was directly applied to a testing dataset; Train/external validation = model was trained on one dataset, validated on another dataset. Bolded text indicates that the method used a concurrently recorded auxiliary signal for artifact management.

Abbreviations: ABP, arterial blood pressure; AUCROC, area under the curve of the receiver operating characteristic; BP, blood pressure; DBP, diastolic blood pressure; DN, dicrotic notch; ECG, electrocardiogram; IBP, invasive blood pressure; ICP, intracranial pressure; ICU, intensive care unit; NIBP, non‐invasive blood pressure; NPV, negative predictive value; PICU, pediatric intensive care unit; PPA, positive predictive accuracy; PPG, photoplethysmogram; PPV, positive predictive value; R, Pearson correlation coefficient; SAH, subarachnoid hemorrhage; SAI, signal abnormality index; SD, standard deviation; SpO_2_, pulse oximetry; SVD, singular value decomposition; TBI, traumatic brain injury; U‐TCN, U‐Net with temporal convolutional network.

#### 3.3.1 Valid pulse identification methods

There were four algorithms proposed that were used to segment and validate a series of blood pressure pulses (Aboy et al., 2005; Asgari et al., 2010; Hoeksel et al., 1996; Urteaga et al., 2024). Despite serving similar functions, there was considerable heterogeneity in the methods proposed to identify valid blood pressure pulses. Aboy et al. (2005), Asgari et al. (2010), and Urteaga et al. (2024) presented very strong results for the accuracy of their respective methods. However, limitations to the metrics presented by Aboy et al. (2005) and Asgari et al. (2010) should be acknowledged. Sensitivity and PPV, among other metrics, are calculated based on entire datasets in which few artifacts are actually present, thus potentially overinflating how these algorithms perform in the presence of abundant artifacts. In both of these articles, they do not disclose the percentage or number of pulses that are artifactual, highlighting a limitation in the literature where the portion of ABP recordings that are valid versus artifactual is poorly documented. Urteaga et al. (2024) only validated the methodology on a small dataset of 377 segments, thus limiting its generalizability. The algorithm presented by Hoeksel et al. (1996) demonstrated modest success in validating pulses when compared to manual annotation. There was minimal information provided regarding which technologies were used to collect data, with the exception of Hoeksel et al. (1996) who indicated that a 7F Swan‐Ganz pulmonary catheter was used. None of these articles mentioned the real‐time applicability of these algorithms.

#### 3.3.2 Dicrotic notch detection methods

The dicrotic notch has been broadly used to demarcate the end of ventricular ejection during systole (Oppenheim & Sittig, 1995) as the temporal location holds valuable information regarding the cardiac cycle (Pal et al., 2024) and is also useful in the derivation of blood pressure metrics such as pulse wave velocity to evaluate arterial stiffness (Hermeling et al., 2009), among others. There were 11 algorithms capable of detecting the dicrotic notch and quantified their respective efficacies (Aguirre et al., 2021; Balmer et al., 2018; Donelli et al., 2002; Hoeksel et al., 1997; Kinias et al., 1981; Li et al., 2010; Oppenheim & Sittig, 1995; Pachauri & Bhuyan, 2012; Pal et al., 2024; Saffarpour et al., 2023; Singh & Sunkaria, 2017). There were several methods outlined that were able to detect the dicrotic notch with high accuracy based on different metrics (sensitivity, specificity, precision, etc.) within a small threshold of error specifically by those developed by Pal et al. (2024) and Balmer et al. (2018). Several of the articles failed to present a threshold for acceptable error for detection. This coupled with differences in the datasets used made it difficult to quantitatively compare the methods. Again, minimal information was provided regarding recording technologies used, with most of these methods being developed on catheter‐based invasive blood pressure data. The exception was Singh and Sunkaria (2017) who used an undisclosed NIBP method. Most articles made no mention of real‐time applicability with the exception of Donelli et al. (2002) and Kinias et al. (1981); however, no data were provided to support their claims.

#### 3.3.3 Pulse onset detection methods

The most basic of these detections is the temporal location of the pulse onset, which was able to be detected by nine of the outlined algorithms (Aguirre et al., 2021; Kyle et al., 1968; Lee et al., 2018; Li et al., 2010; Pachauri & Bhuyan, 2012; Singh & Sunkaria, 2016; Singh & Sunkaria, 2017; Xu et al., 2009; Zong et al., 2003). The ability to detect and delineate a clear onset of BP pulses is a potential indicator for the overwhelming presence of noise or artifacts. There were again several methods that presented strong results for the identification of the pulse onset based on metrics of sensitivity and PPV, specifically those developed by Li et al. (2010), Singh and Sunkaria (2016), Singh and Sunkaria (2017), Aguirre et al. (2021), and Pachauri and Bhuyan (2012), with each of these methods being able to identify the pulse onset with reported sensitivities and specificities frequently exceeding 99%. However, several of these articles did not report the threshold of temporal error in the detection that was acceptable. Lee et al. (2018) did not report a sensitivity or specificity value but did indicate that their algorithm was able to identify the pulse onset within an extremely small threshold of error. The method presented by Xu et al. (2009) was the only method that was reliant on an auxiliary signal (ECG). Again, there was limited information provided regarding commercial or custom recording technologies used and no mention of real‐time applicability.

#### 3.3.4 Systolic peak detection methods

There were nine algorithms presented capable of detecting the systolic peak (Aguirre et al., 2021; Kinias et al., 1981; Kyle et al., 1968; Li et al., 2010; Nygårds et al., 1976; Pachauri & Bhuyan, 2011; Pachauri & Bhuyan, 2012; Singh & Sunkaria, 2016; Singh & Sunkaria, 2017) and provided quantification of the effectiveness of the presented algorithms. The systolic peak is similarly a fundamental aspect of the BP pulse, and the absence of the ability to detect this portion of the waveform could be used to describe artifacts. Similar to the pulse onset detection, there were several articles that presented high sensitivity and PPV values, with Li et al. (2010), Singh and Sunkaria (2016), Singh and Sunkaria (2017), Aguirre et al. (2021), and Pachauri and Bhuyan (2012) again demonstrating values consistently above 97%. The articles did not provide information on any particular custom or commercial recording technologies used. Only two methods mentioned real‐time utility (Kinias et al., 1981; Nygårds et al., 1976) without evidence.

#### 3.3.5 Other fiducial point detection methods

Additional points on the BP waveform that were detected using the described algorithms include the percussion and tidal peaks (Paradkar & Chowdhury, 2015), dicrotic peaks (Almeida et al., 2011; Pachauri & Bhuyan, 2012), end systole (Balmer et al., 2021), ejection onset (Kinias et al., 1981; Starmer et al., 1973), end diastole (Kinias et al., 1981), ejection end (Starmer et al., 1973), pre‐ejection time (Nygårds et al., 1976), left ventricular ejection time (Nygårds et al., 1976), and reflection point (Almeida et al., 2011). There were three algorithms that were not able to be categorized with others as they did not quantify the detection of the dicrotic notch, pulse onset, and systolic blood pressure. Swatzell et al. (1973) identified systolic time as a means for detecting waveforms. Almeida et al. provided a combined effectiveness metric for the accurate detection of the systolic peak, reflection peak, dicrotic peak, and dicrotic notch (Almeida et al., 2011). Paradkar and Chowdhury (2015) and Prakash et al. (2008) developed a method for detection of the systolic peak, percussion peak, tidal peak, and diastolic peak, providing a combined metric for effectiveness.

A majority of the algorithms designed for fiducial point detection had no mention of real‐time applications of these algorithms. There were five algorithms that were referred to as live‐time algorithms; however, this was not validated (Donelli et al., 2002; Hoeksel et al., 1997; Kinias et al., 1981; Nygårds et al., 1976; Swatzell et al., 1973). There was also limited validation conducted using additional external datasets. Only the Hoeksel et al. (1997) algorithm was trained using data recorded from dogs and tested on a human population. It was also extended to a new dataset by Donelli et al. (2002) for further external validation. The Paradkar and Chowdhury (2015) algorithm was the only method that was externally validated. However, it can be concluded that there is a substantial body of literature available for the detection of valid waveform and fiducial points that has the potential to be leveraged in artifact management of ABP signals. Almeida et al. (2011) used a specific non‐invasive piezometric probe for data recording; this had a baseline wander removal circuit. Swatzell et al. (1973) used a glycerin pellet connected to a catheter to record ABP data. Paradkar and Chowdhury (2015) did not specify a recording technology. None of these methods mention real‐time application.

## 4 DISCUSSION

ABP signals in cardiovascular and hemodynamic monitoring have widespread utility across healthcare. As a result of these signals being recorded in real time at the patient bedside, both IBP and NIBP sources are often rife with artifacts from a variety of different origins. Due to the implications on signal utility and the limitations of manual annotations, an automated approach to detect artifacts or validate signal pulses has been a long‐time subject of research. The primary objective of this review was to determine which methods have been developed and critically analyze and compare their effectiveness and utility. The extensive research in this domain is evidenced by the 73 articles that satisfied the inclusion criteria of this review. The algorithms presented in each of these articles were categorized as artifact management methods for low‐frequency (<100 Hz) ABP signals, artifact management methods for full waveform (≥100 Hz) ABP signals, and methods designed to identify fiducial points or valid waveforms.

The algorithms developed for artifact management in low frequency signals were able to perform well in identifying artifacts according to various metrics. Machine learning models were able to achieve strong results by metrics of sensitivity and specificity. However, in one of the only articles in which there is a quantitative comparison of diverse methods, presented by Maleczek et al. (2024), it is indicated that the LSTM machine learning model did not dramatically outperform thresholding or statistical deviation‐based methodologies, potentially calling into question the trade‐off between the computational complexity of a machine learning model compared to a more basic approach, specifically on low‐frequency data. While low‐frequency data provides instrumental information that guides clinical decisions, there has been a natural movement in clinical care towards continuous monitoring of ABP, necessitating full‐waveform artifact management. Full‐waveform artifact management methods are required to identify more minute and complex errors present in these signals, specifically, oscillatory or morphological artifacts that cannot be addressed using simple thresholding methods. It appears the literature has responded to this notion. Across the algorithms that had been designed to detect artifacts in full waveform ABP signals, there were strong performing methods by metrics of sensitivity, specificity, and PPV in machine learning, wavelet, and signal quality‐based algorithms. There were several algorithms that were designed to detect fiducial points, including the dicrotic notch, pulse onset, and systolic peak, with many of these algorithms achieving high sensitivity, specificity, and PPV values despite considerable differences in the types of methodologies that were applied. There were fewer articles that directly assessed the validity of ABP waveforms.

### 4.1 Limitations of literature

An objective of this systematic review was to elucidate a leading methodology for either artifact or valid pulse identification. There were several articles that presented algorithms with remarkable success in artifact identification/valid pulse detection for both low‐ and high‐frequency ABP signals. However, the limitations within the literature highlight sources of the potential presence of bias within these results which must be acknowledged and rectified prior to any widespread conclusions can be drawn from this effectiveness.

First, many of these studies demonstrated limited generalizability. There were insufficient sample sizes and population diversity in most of the articles in this review. Individuals differ considerably in cardiovascular physiology both within and between groups potentially impacting the morphology of the artifacts present in ABP signals. A lack of validation of artifact management methods on an adequately physiologically diverse population of sufficient size limits confidence that the developed method would have similar performance across ABP monitoring. It also limits proper comparison of artifact management methods between articles, as datasets with insufficient size and physiological variance are more likely to have differing artifact types. Furthermore, there are few articles that perform any analysis of the effectiveness of algorithms when applied to different patient population groups to better understand the implications of varying physiology.

Second, several of the articles did not evaluate effectiveness or investigate the implications of varying the recording methodologies. There are several differences between different NIBP or IBP technologies, specifically the sampling rates, location(s) of measurement, and internal algorithmic functionality within NIBP technology. This may have resulted in differences in the ABP signals measured and their artifacts. Information regarding the recording technologies, specifically for NIBP methods, was often lacking across the literature. This limits the confidence that a method developed using one recording technology with a particular sampling rate would extend to another without a decrease in functionality. Furthermore, this made it difficult to compare the effectiveness between methods presented in different articles.

Third, there was insufficient reporting of the effectiveness of presented methods on specific artifact types. Several articles mentioned and provided examples of different artifact types specific to motion, cuff‐inflation errors, signal noise, and flushing; however, there was seldom data presented on the effectiveness for specific types. This is of particular significance as, due to the plethora of artifact sources and their varied morphologies, it may be necessary to employ multiple methods to adequately identify them.

Fourth, there were several opportunities where bias could have been introduced in manual annotation that could have contributed to the consistently high accuracy reported by many of the articles included in this review. Across several of the articles included in this review, annotations were often only conducted by one to three individuals serving as the ground truth for annotations. Additionally, there was a consistent absence of adequate descriptions of what was deemed “valid” compared to “artifactual.” The use of external validation is imperative to circumvent this potential bias to ensure that the presented results are not overly inflated by the ground truth being tailored to the strengths of the proposed algorithm.

Fifth, there was inadequate consideration of the real‐time utility of these algorithms or reporting of lag time required. The utility of automated retrospective artifact identification alleviates a significant burden on personnel conducting this manually. However, a critical application of the algorithms is to reduce false alarms in critical care settings, as well as to improve the accuracies of derived metrics to measure cardiovascular health to potentially guide treatment. As a result, live‐time utility is paramount. This was seldom mentioned across the articles in this review. This is important to consider in the development of future methods, as despite the benefits in accuracy to artifact identification that can be fostered using a big‐data machine learning approach or a signal quality analysis method that evaluates a large number of morphological features, the processing lag required for real‐time use needs to be a parameter considered in the design of such algorithms. Much of the existing body of literature not only fails to adequately describe the associated time lag in live artifact identification but also does not describe the computational processing requirements. This is an additional potential limitation of more cumbersome models as it may impact the utility of these models in ICUs and other clinical settings that are without adequate computational capacity.

Sixth, it was difficult to compare the performance of methods between articles due to the lack of consistency in the compositions of the datasets. Among artifact identification articles, metrics including sensitivity, specificity, PPV, NPV, and AUCROC were consistently used throughout. These metrics are different combinations of the relationships between true positives, true negatives, false positives, and false negatives. However, within these similar metrics, there is considerable variability. This variability included differences in artifact identification being calculated on a point‐by‐point basis or a pulse‐by‐pulse basis. Additionally, the issue of class imbalance was not addressed. Several of these articles stipulated the proportion of their recording that was artifactual; however, there was vast heterogeneity in terms of the differences in proportion. In the instances of a “true positive” being classified as a correctly identified artifact and a “true negative” being classified as a valid point or pulse, a disproportionately large number of valid pulses can result in strong performances by metrics of specificity, NPV, and accuracy, despite poor performance on artifacts. There were seldom any data balancing techniques that were conducted to minimize class imbalance.

There are also potential concerns with model interpretability of many of the methods presented for artifact management. Thresholding‐based methods are relatively simple to understand; as such, in a clinical setting, it is easy for clinicians to understand which ABP measurements are being removed from the signal. However, difficulty in model interpretability in more complex models, specifically those that involve the use of machine learning techniques, could result in clinicians being cautious of using these artifact management methods with fears of the algorithms performing inadequately. An example of this may be the removal of certain unusual cardiac events due to their unusual morphology resulting in a potential missed or inaccurate diagnosis. There were very few articles included in this review that provided any specific categorization of the artifact types that were identified or removed. Howard et al. (2019) provided a distribution of detected artifacts in an aortic BP signal that included the catheter “port‐open,” “other artifact,” and “noisy.” Such subcategorization could be instrumental in the interpretability of the model outputs and clinical applicability. For more widespread applicability of these models, it may be necessary to integrate machine learning classification into a self‐supervised model that allows for clinicians to interact with and validate the labels generated by the algorithm to boost confidence in the results of the artifact management. This could take the form of a contrastive learning self‐supervised model that would allow for clinicians to review batches of labeled artifacts quickly.

While the fiducial point identification algorithms also performed well, identifying these points within a small tolerance, it should be noted that an algorithm of this nature would not be sufficient in isolation to identify all artifact types. These methods identify certain fiducial points; however, these points could potentially be detected in artifactual pulses. As a result, there are unique pulse‐like or oscillatory artifacts that may go undetected. A more robust study is required on the ability to use the presence/absence fiducial points to directly validate pulses, as these studies were primarily focused on accurate identification of these points. Understanding the implications of artifacts on these points is, therefore, required in the future. Limitations of Review.

There were some limitations associated with the scope of this review. Exclusion criteria for the articles in this review included the requirement that the article be published in English, introducing a potential language bias. There was an additional limitation that the search was initially conducted on August 27, 2024, and updated on May 14, 2025, with studies after this date not being included in the review. Articles that presented valid pulse or valid point identification algorithms were included in the review; however, the search string was not specifically tailored to include them. Additionally, while the search included BIOSIS, SCOPUS, EMBASE, PubMed, and Cochrane Library were intended to encapsulate all indexed articles in this domain, it is possible that articles could have been missed. The lack of a homogeneous study type, with variations in sample size, differences in tolerance for labeling, and sampling methodologies, may have resulted in inadequate or insufficient conclusions made due to a lack of information sufficient to facilitate a proper meta‐analysis of the literature.

### 4.2 Recommendations

There are several promising methods outlined in this review that enable highly accurate artifact management. As a result, it is recommended to build on these outlined methodologies while reducing the presence of potential bias. By limiting this bias, the result is a more generalizable end product. Additionally, a method with less potential bias is also able to be compared to others with more accuracy, strengthening the conclusions. There are two ways in which this bias can be limited. The first involves federated data sharing of datasets composed of raw ABP signals as well as a manually annotated ground truth, enabling an easier comparison between methods. However, it is acknowledged that, due to privacy concerns with human data sharing, this may not be possible. An alternative approach is that algorithms developed be developed using more robust datasets. This would involve training and evaluating a method on a physiologically heterogeneous patient population with variation in the recording methodology and sampling rates used. An additional recommendation is an improved consideration of the real‐time utility of these algorithms through the presentation of data regarding computational efficiency and computational requirements for the model. Adequate documentation in future study enables a better comparison between methods and also helps establish requirements for the environment in which the algorithm could potentially be used.

## 5 FUTURE DIRECTIONS

The literature suggests that several methods have been able to attain high sensitivity and specificity results consistently above 95%; however, an autonomous method that is able to reach 100% by these metrics is much more difficult. A more nuanced approach will be required, adequately addressing the morphological variability of the artifacts exhibited in different ABP signals. Future work by our lab will aim to rectify this. A layered artifact pipeline has been proposed, which will involve the development and application of combinations of the following artifact identification techniques:

1. Thresholding‐based methods—as has been discussed in this review, this method will involve establishing amplitude and statistical deviation thresholds to identify artifacts.
2. Time‐series autoregression‐based method—these statistical analysis‐based methods will involve modeling the ABP signal and detecting significant deviations from the statistical structure as artifactual, as has been identified in previous work from our lab group in other pressure‐flow physiological signals. An ARIMA model can capture the dynamic structure of time‐series signals and be used as a means of artifact identification (Islam et al., 2025).
3. Wavelet or Fourier transformation‐based methods—this will involve the transformation of time‐series ABP signals into the time‐frequency and frequency domains, respectively, to facilitate the detection of erroneous oscillations in the signals as artifacts. Wavelet‐based methodologies were employed in articles included in this review; several of them focused more on the identification of baseline drift (Xu et al., 2005; Xu et al., 2007) or filtering (Nguyen et al., 2015). It appears that the use of the wavelet transformation to identify artifacts within the time‐frequency domain using known physiological oscillations as a baseline has not been thoroughly vetted.
4. Waveform morphology detection‐based methods—a catalog will be developed using archived ABP data that will contain various artifactual segments of data that can be used in the categorization of incoming pulses, following methodologies like those presenting waveform quality assessment of segmented beats (Zong et al., 2004).

The techniques listed will be able to be applied using an RNN and CNN, merging them into a single autonomous artifact management pipeline. The integration of an RNN into this pipeline will help augment the accuracy of the thresholding and time‐series autoregressive‐based methods. The training of an integrated RNN model enables these methods to become dynamic in the selection of thresholding parameters as well as improving the precision with which artifacts are detected using autoregressive modeling. The training and implementation of a CNN will enable more accurate detection and sub‐categorization of using both wavelet/Fourier transformation‐based as well as waveform morphology‐based methods. This pipeline will allow for a litany of artifact types witnessed in ABP signals to be identified with the different artifact types identified, similar to articles included in this review (Howard et al., 2019; Imhoff et al., 1998; Pike & Mustard, 1992; Zhang et al., 2010). Additionally, there will be active consideration of the ability of this algorithm to function in real time and what benefits may arise from a more complex pipeline compared to the efficiency of use of a simpler model. We will evaluate the use of methods (1)–(4) each in isolation as well as different combinations of them to elucidate the method that most successfully balances effectiveness (sensitivity, PPV, and AUROC) with efficiency in computational time and requirements. The metrics selected are not prone to class imbalance as they directly measure the performance on artifacts and are not inflated by an abundance of valid pulses. These models will also be constructed using multiple cohorts of varying pathophysiology to examine the difference in performance. These datasets have been manually cleaned by trained personnel; as a result, the algorithms developed will be tested against this standard. This will result in a developed automated method to closely mimic the accuracy of manual annotation. An assessment of the accuracy, sensitivity, and specificity of the proposed methods will be conducted across a documented list of artifact types, specifying the recording methodology and the sampling rate of the dataset used. It will also be developed and validated among the largest datasets applied in this domain to ensure the robustness of the effectiveness. As was highlighted among the limitations of the literature, a more complex method must be interpretable for clinical use. As such, self‐supervised methodologies will be evaluated to potentially enable clinician engagement with the algorithm. Using methods such as contrastive learning, clinicians would be able to quickly label categories of identified artifact types, both providing important insight for improvement of the algorithm but also boosting the confidence of the clinician in the output of artifact management.

These considerations in future work will follow the prescribed recommendations for a more generalizable and useful end product for a semi‐ or fully automated artifact identification that is currently lacking in the current work.

As a basis for future work, Table 4 displays a proposed checklist for the reporting of future artifact detection algorithms.

**TABLE 4:** Proposed checklist for reporting an artifact identification or removal method.

Category	Item no.	Checklist item	Section where it is located
Signal type	1	Identification of invasiveness of recording	
	2	Identification of the location of ABP recording	
Dataset information	3	Disclosure of if data/annotations are publicly available	
	4	Sources of datasets	
	5	Subject physiological information	
	6	Recording methodology information	
	7	Identification of pertinent training of individuals performing annotations	
	8	Availability of other simultaneously recorded signals for annotations	
	9	Size and split of data (training, validation, testing)	
	10	Use of external validation dataset	
	11	Proportion of data that is valid or artifactual	
	12	Strategies used to limit class imbalance	
Reporting of effectiveness	13	Metrics of effectiveness (and their formulae) used	
	14	Measurement of real‐time utility of model, including computational requirements	
Post‐processing	15	Description of any interpolation methodologies included within this method	
Availability of code	16	Disclosure of whether artifact management method is open source	

*Note*: The completion and inclusion of this brief table will allow for information regarding a novel artifact management method to be quickly disseminated and will ideally serve as a baseline checklist for a generalizable solution.

## 6 CONCLUSION

A search was conducted across five databases to investigate the current body of literature pertaining to artifact management in ABP signals. This systematic scoping review identified 73 articles capable of quantifiably identifying artifactual segments; these were categorized into three groups: (1) artifact management in low‐frequency (<100 Hz) ABP signals, (2) artifact management in full‐waveform (≥100 Hz) ABP signals, and (3) identification of valid or fiducial points in ABP signals. There were several methodologies in each of these categories that presented strong results for accurate artifact detection. However, there were several sources of limitations. Generalizability was a recurring limitation in many of these studies due to the small sample size, a lack of validation on individuals of varied pathophysiology, and an insufficient variance in the recording methodologies and sampling rates used. There was also a distinct lack of discussion regarding the real‐time utility of many of these developed methods. A functional and widely accepted method for artifact identification and removal will result in a reduction in nurse alarm fatigue and an increased clinical accuracy of time‐series ABP signals and derived metrics to provide streamlined accurate diagnoses and prognoses. Future work is required to rectify the limitations identified in this review before such a method can be distinguished.

## AUTHOR CONTRIBUTIONS

Conceptualization, T.B. and F.A.Z.; methodology, T.B. and F.A.Z.; validation, N.V., A.S.S., K.Y.S., X.N.S., N.S., J.M., M.H., L.F., and F.A.Z.; formal analysis, T.B.; investigation, T.B.; resources, F.A.Z.; data curation, T.B.; writing—original draft preparation, T.B.; writing—review and editing, N.V., A.S.S., K.Y.S., X.N.S., N.S., J.M., M.H., L.F., and F.A.Z.; visualization, F.A.Z. and T.B.; supervision, F.A.Z. and L.F.; project administration, F.A.Z.; funding acquisition, F.A.Z. All authors have read and agreed to the published version of the manuscript.

## FUNDING INFORMATION

This work was directly supported through the Endowed Manitoba Public Insurance (MPI) Chair in Neuroscience and the Natural Sciences and Engineering Research Council of Canada (NSERC; ALLRP‐578524‐22, Canadian Graduate Scholarship—Master's [CGS‐M], and Postgraduate Scholarship—Doctoral [PGS‐D‐600433‐2025]).

## CONFLICT OF INTEREST STATEMENT

FAZ currently has an NSERC Alliance Advantage grant (ALLRP‐597708‐24) support in partnership with Medtronic's Acute Care & Monitoring Division (ERP‐2024‐14025) for work that is unrelated to this manuscript. Funding from the partner organization is provided to match NSERC governmental funding only, in keeping with NSERC policies. Medtronic does not direct the research objectives, data collection, analysis, interpretation, or publication of the findings in any way. The other authors report no conflicting interests.

## Supporting information


Data S1.

## Data Availability

All relevant data are contained in the manuscript and the supporting information.
