# Supplementary material for: Artifact management methodologies for arterial blood pressure signals: A systematic scoping review of human and animal literature
Source: Physiol Rep. 2025 Oct 2;13(19):e70533. doi: 10.14814/phy2.70533 (PMC12491656; doi:10.14814/phy2.70533)
Supplement: Supplementary file 1 — Data S1. [file PHY2-13-e70533-s001.docx]

# Supplementary File 1

**Table A1. PRISMA ScR Checklist**

| **Section and Topic** | **Item #** | **Checklist item** | **Location where item is reported** |
| --- | --- | --- | --- |
| **Title** | | |  |
| Title | 1 | Identify the report as a systematic review. | Pg. 1 (Title) |
| **Abstract** | | |  |
| Abstract | 2 | See the PRISMA 2020 for Abstracts checklist. | Pg. 5 (Abstract) |
| **Introduction** | | |  |
| Rationale | 3 | Describe the rationale for the review in the context of existing knowledge. | Pg. 6-7 (Section 1) |
| Objectives | 4 | Provide an explicit statement of the objective(s) or question(s) the review addresses. | Pg. 7 (Section 1) |
| **Methods** | | |  |
| Eligibility criteria | 5 | Specify the inclusion and exclusion criteria for the review and how studies were grouped for the syntheses. | Pg. 8 (Section 2.2) |
| Information sources | 6 | Specify all databases, registers, websites, organisations, reference lists and other sources searched or consulted to identify studies. Specify the date when each source was last searched or consulted. | Pg. 9 (Section 2.3) |
| Search strategy | 7 | Present the full search strategies for all databases, registers and websites, including any filters and limits used. | Pg. 9 (Section 2.3) + Pg. 55 (Appendix B) |
| Selection process | 8 | Specify the methods used to decide whether a study met the inclusion criteria of the review, including how many reviewers screened each record and each report retrieved, whether they worked independently, and if applicable, details of automation tools used in the process. | Pg. 9(Section 2.4) |
| Data collection process | 9 | Specify the methods used to collect data from reports, including how many reviewers collected data from each report, whether they worked independently, any processes for obtaining or confirming data from study investigators, and if applicable, details of automation tools used in the process. | Pg. 9 (Section 2.5) |
| Data items | 10a | List and define all outcomes for which data were sought. Specify whether all results that were compatible with each outcome domain in each study were sought (e.g. for all measures, time points, analyses), and if not, the methods used to decide which results to collect. | Pg. 9 (Section 2.5) |
|  | 10b | List and define all other variables for which data were sought (e.g. participant and intervention characteristics, funding sources). Describe any assumptions made about any missing or unclear information. | N/A |
| Study risk of bias assessment | 11 | Specify the methods used to assess risk of bias in the included studies, including details of the tool(s) used, how many reviewers assessed each study and whether they worked independently, and if applicable, details of automation tools used in the process. | All articles published in academic journals, as such, biases were assumed to have been screened |
| Effect measures | 12 | Specify for each outcome the effect measure(s) (e.g. risk ratio, mean difference) used in the synthesis or presentation of results. | N/A |
| Synthesis methods | 13a | Describe the processes used to decide which studies were eligible for each synthesis (e.g. tabulating the study intervention characteristics and comparing against the planned groups for each synthesis (item #5)). | N/A |
|  | 13b | Describe any methods required to prepare the data for presentation or synthesis, such as handling of missing summary statistics, or data conversions. | N/A |
|  | 13c | Describe any methods used to tabulate or visually display results of individual studies and syntheses. | All data items for each method were tabulated and are included in Appendix C and D (Table C.1-C.6, D.1-D.4, E.1-E.4) |
|  | 13d | Describe any methods used to synthesize results and provide a rationale for the choice(s). If meta-analysis was performed, describe the model(s), method(s) to identify the presence and extent of statistical heterogeneity, and software package(s) used. | N/A |
|  | 13e | Describe any methods used to explore possible causes of heterogeneity among study results (e.g. subgroup analysis, meta-regression). | N/A |
|  | 13f | Describe any sensitivity analyses conducted to assess robustness of the synthesized results. | N/A |
| Reporting bias assessment | 14 | Describe any methods used to assess risk of bias due to missing results in a synthesis (arising from reporting biases). | Pg. 33-34 (Section 4.1) |
| Certainty assessment | 15 | Describe any methods used to assess certainty (or confidence) in the body of evidence for an outcome. | N/A |
| **Results** | | |  |
| Study selection | 16a | Describe the results of the search and selection process, from the number of records identified in the search to the number of studies included in the review, ideally using a flow diagram. | Pg. 9-10 (Section 3) |
|  | 16b | Cite studies that might appear to meet the inclusion criteria, but which were excluded, and explain why they were excluded. | Pg. 24 (Section 3.3) |
| Study characteristics | 17 | Cite each included study and present its characteristics. | Pg. 11-32 (Section 3.1 - 3.3) |
| Risk of bias in studies | 18 | Present assessments of risk of bias for each included study. | All articles published in academic journals, as such, biases were assumed to have been screened |
| Results of individual studies | 19 | For all outcomes, present, for each study: (a) summary statistics for each group (where appropriate) and (b) an effect estimate and its precision (e.g. confidence/credible interval), ideally using structured tables or plots. | Appendix C and D (Table C.1-C.6, D.1-D.4, E.1-E.4) |
| Results of syntheses | 20a | For each synthesis, briefly summarise the characteristics and risk of bias among contributing studies. | All articles published in academic journals, as such, biases were assumed to have been screened |
|  | 20b | Present results of all statistical syntheses conducted. If meta-analysis was done, present for each the summary estimate and its precision (e.g. confidence/credible interval) and measures of statistical heterogeneity. If comparing groups, describe the direction of the effect. | No statistical synthesis conducted |
|  | 20c | Present results of all investigations of possible causes of heterogeneity among study results. | Pg. 11-32 (Section 3.1 - 3.3) |
|  | 20d | Present results of all sensitivity analyses conducted to assess the robustness of the synthesized results. | Pg. 11-32 (Section 3.1 - 3.3) |
| Reporting biases | 21 | Present assessments of risk of bias due to missing results (arising from reporting biases) for each synthesis assessed. | Pg. 34 (Section 4.2) |
| Certainty of evidence | 22 | Present assessments of certainty (or confidence) in the body of evidence for each outcome assessed. | N/A |
| **DISCUSSION** | | |  |
| Discussion | 23a | Provide a general interpretation of the results in the context of other evidence. | Pg. 32-33 (Section 4) |
|  | 23b | Discuss any limitations of the evidence included in the review. | Pg. 33-34 (Section 4.1) |
|  | 23c | Discuss any limitations of the review processes used. | Pg. 34 (Section 4.2) |
|  | 23d | Discuss implications of the results for practice, policy, and future research. | Pg. 35-36 (Section 4.3 – 4.4) |
| **OTHER INFORMATION** | | |  |
| Registration and protocol | 24a | Provide registration information for the review, including register name and registration number, or state that the review was not registered. | Review was not registered |
|  | 24b | Indicate where the review protocol can be accessed, or state that a protocol was not prepared. | Protocol not prepared |
|  | 24c | Describe and explain any amendments to information provided at registration or in the protocol. | N/A |
| Support | 25 | Describe sources of financial or non-financial support for the review, and the role of the funders or sponsors in the review. | Pg. 3-4 (Funding) |
| Competing interests | 26 | Declare any competing interests of review authors. | Pg. 4 (Conflicts of Interest) |
| Availability of data, code and other materials | 27 | Report which of the following are publicly available and where they can be found: template data collection forms; data extracted from included studies; data used for all analyses; analytic code; any other materials used in the review. | N/A |

1. **Supplementary File 2**

The following search string was entered into five databases (BIOSIS, SCOPUS, EMBASE, PubMed, and Cochrane Library) searching keywords, abstracts, and titles indexed in these databases:

“Arterial blood pressure” OR “Arterial Pressures” OR “Pressures, Arterial” OR “Arterial Blood Pressure” OR “Arterial Blood Pressures” OR “Blood Pressures, Arterial” OR “Pressure, Arterial Blood” OR “Pressures, Arterial Blood” OR “Arterial Tension” OR “Arterial Tensions” OR “Tension, Arterial” OR “Tensions, Arterial” OR “Blood Pressure, Arterial” OR “Mean Arterial Pressure” OR “Arterial Pressure, Mean” OR “Arterial Pressures, Mean” OR “Mean Arterial Pressures” OR “Pressure, Mean Arterial” OR “Pressures, Mean Arterial” OR “Aortic Pressure” OR “Aortic Pressures” OR “Pressure, Aortic” OR “Pressures, Aortic” OR “Aortic Blood Pressure” OR “Aortic Blood Pressures” OR “Blood Pressures, Aortic” OR “Pressure, Aortic Blood” OR “Pressures, Aortic Blood” OR “Aortic Tension” OR “Aortic Tensions” OR “Tension, Aortic” OR “Tensions, Aortic” OR “Blood Pressure, Aortic” OR “Aortic Pulse Pressure” OR “Aortic Pulse Pressures” OR “Pressure, Aortic Pulse” OR “Pressures, Aortic Pulse” OR “Pulse Pressure, Aortic” OR “Pulse Pressures, Aortic” OR “Mean Aortic Pressure” OR “Aortic Pressure, Mean” OR “Aortic Pressures, Mean” OR “Mean Aortic Pressures” OR “Pressure, Mean Aortic” OR “Pressures, Mean Aortic” OR “ABP” OR “MAP” OR “Blood Pressure”

AND

“Artifact management techniques” OR “Artefact management techniques” OR “Artifact management” OR “Artefact management” OR “Artifact removal method” OR “Artefact removal method” OR “Artifact removal” OR “Artefact removal” OR “Artifact management system” OR “Artefact management system” OR “Artifact removal technique” OR “Artefact removal technique” OR “Error management” OR “Error removal” OR “Error management method” OR “Error removal method” OR “Error management technique” OR “Error removal technique” OR “Error management system” OR “Error removal system” OR “Artifact identification techniques” OR “Artefact identification techniques” OR “Error identification techniques” OR “Artifact identification” OR “Artefact identification” OR “Error identification” OR “Artifact identification method” OR “Artefact identification method” OR “Error identification method” OR “Artifact identification system ” OR “Artefact identification system” OR “Error identification system” OR “artifact” OR “artefact” OR “artifacts” OR “artefacts” OR “noise removal”

1. **Supplementary File 3**

**Table C.1. Low-frequency ABP artifact detection – thresholding-based methods**

| **Reference** | **Subject information** | | **Data type (sampling rate) – system type** | | **Artifact removal method(s) used** | **Methods compared** | **Effectiveness** | **Study results and conclusions** | **Limitations** |
| --- | --- | --- | --- | --- | --- | --- | --- | --- | --- |
| Keselbrener and Akselrod  [33] | **Subjects** | 2 | **Signal type(s) used** | NIBP (50 Hz with effective sampling of 25 Hz) | This algorithm was designed to identify artifacts in the NIBP signal when the Finapres device adjusts its cuff pressure. A threshold was established between expected maximum and minimums of ABP signal, in case of interruptions in signal, the difference shrinks as do those of neighboring points identifying an ‘interruption’. If a segment of signal falls below a defined threshold and its neighboring pulses fall below a second defined threshold, an interruption is identified.  **Validation:** Model was developed without training and was directly applied to a testing dataset  **Post-processing:** The last complete pulse before the interruption and the full beat immediately after the interruption are used to interpolate the artifactual segment. | Before and after correction, no further information provided regarding the reference standard | Spectral parameters were examined for both subjects before and after the correction. A reduction in these frequencies is intended to indicate a decrease in artifacts at identified frequencies  Distinct decrease in signal power in low frequencies (LF) (0-0.072 Hz) and high frequencies (HF) (0.22-0.35 Hz) for adult ABP signal.  LF: 146 to 140 (unspecified units of power)  HF: 74 to 35  (unspecified units of power)  No change in high frequency but significant change for low frequency in child’s ABP signal pre- and post-correction  LF: 154 to 119  (unspecified units of power)  Decrease in overall power of signal in both adult and child  Child: 316 to 277  (unspecified units of power)  Adult: 289 to 181  (unspecified units of power) | Interruptions corrected using method proposed  Power of entire spectrum of ABP signal for adult subject was reduced (in all frequencies), but specifically in high frequencies  Spectrum for ABP signal of child showed a decrease in the power in low frequency range after correction | Small cohort  Artifact identification and removal is limited to specific artifact type  Keeps signal close to ‘reality’ and only minimally impacts the ‘true HRF content’ |
|  | **Sex (% Male)** | 50% |  |  |  |  |  |  |  |
|  | **Age** | 24 (M) and 6 (F) | **Device type** | Finapres |  |  |  |  |  |
|  | **Pathophysiological health** | Healthy | **Location** | Finger |  |  |  |  |  |
| Clark et al. [46] | **Subjects** | 15 | **Signal type(s) used** | ABP (0.0011 Hz) | The basis of the method was to identify potentially artifactual data points to reduce the reliance on clinicians to look at them. Blood pressure is treated as bivariate data (x_1_ is DBP, x_2_ is SBP). The first method indicates that approximately 5% of all values should be farther than two standard deviations from the mean. The second method uses a transformation to remove any correlation between bivariate data points. Thresholds are then established based on mean and variance of SBP and DBP values. These thresholds are used to detect outliers The third method uses the distance formula and establishes a cutoff distance that indicates an error.  **Validation:** Model was developed without training and was directly applied to a testing dataset  **Post-processing:** Rejection/removal of artifactual points | No use of algorithm | Reduction of 95.6% of readings that clinicians had to look at (917 to 40) | Can reduce the amount of time clinicians spend at evaluating readings by automatically identifying outliers | Method must be applied retrospectively as the criterion must be developed first before being applied  Non-continuous blood pressure  BP measurements were taken while individuals were doing daily activities |
|  | **Sex (% Male)** | 60% |  |  |  |  |  |  |  |
|  | **Age** | 49.5 (20 to 66) | **Device type** | N/A |  |  |  |  |  |
|  | **Pathophysiological health** | Mild untreated hypertension | **Location** | N/A |  |  |  |  |  |
| Cao et al.  [47] | **Subjects** | 10 from 153 | **Signal type(s) used** | BP (0.017 Hz), HR | Various methods used for artifact identification, they are combined into the Detector:  1. Limit-based detectors - thresholds defined based on expected lower and upper limits defined using physiological values  2. Deviation-based detectors – involves first defining moving time window, thresholds are defined as distance from standard deviation in each moving window, if all previous points in window are non-artifactual and the segment is flagged, it is presumed that it is the last point responsible for the high variability (allows for the artifact to be pinpointed)  3. Correlation-based detector – for points that are not quite satisfying the previous two method thresholds but also correspond to a recent HR artifact, it is removed  Additionally, if artifacts are within a certain distance of each other, the entire segment is removed  Criteria optimized for each infant  **Validation:** Model was developed without training and was directly applied to a testing dataset  **Post-processing:** Removal is conducted if artifact is located using limit-based method that the mean of the windows previous *t-1* points are used. If it is the other methods, a ratio of previous and future points is used to interpolate | Domain expert | Detected sensitivity in BP of 94.2% (SD=5.3%)  and specificity of 80.0% (SD=12.4%) | Integrated method constructed to identify artifacts CVDetector | Single center small cohort  Developed for very slow sampling rate data |
|  | **Sex (% Male)** | N/A |  |  |  |  |  |  |  |
|  | **Age** | Infant | **Device type** | N/A |  |  |  |  |  |
|  | **Pathophysiological health** | Preterm | **Location** | N/A |  |  |  |  |  |
| Du et al.  [48] | **Subjects** | 54 of 78 without interruptions longer than 5 minutes | **Signal type(s) used** | IBP (MAP, SBP, DBP) (0.017 Hz) | Algorithm for error checking involves:  1. Removing missing values  2. Thresholds defined for each signal for acceptable difference between consecutive measurements. For MAP, SBP, and DBP these values are 6, 8, and 5 mmHg, respectively.  3. Iterate through data time when arterial line catheter has been stably placed and then removed based on established criteria, setting all previous and proceeding values to NA  4. Establish the temporal spacing for DBP, MAP, and SBP based on calculated ratio  5. Determine whether spacing between pulses is correct compared to neighbors, if not, interpolate  6. Use thresholds between consecutive measured values to determine whether it is an artifact for DBP, MAP, and SBP, with suppression to prevent false positives  7. Interpolate for all NA values  8. Use unsigned distances between DBP, MAP, SBP to determine artifacts  9. If within a certain length, interpolation conducted  **Validation:** Model was developed without training and was directly applied to a testing dataset  **Post-processing:** linear interpolation using neighboring points | Review by human expert (anaesthesiologist) | Algorithm had sensitivity of 87.0% and specificity of 99.4%  The results in the first 15 minutes of recordings were 91% and 98%, respectively.  Humans and algorithm had similar results in assigning beginning and end of recording, unsigned distance was 0.17 min (SD of 0.61) and 2.1 min (SD of 5.1) for start and end times, respectively. | Regions of disagreement largely due to high BP variability where they were unsure  Algorithm effectively captures artifacts compared to humans | Does not provide sampling rate  Unclear definition of artifacts |
|  | **Sex (% Male)** | N/A |  |  |  |  |  |  |  |
|  | **Age** | N/A | **Device type** | Arterial line |  |  |  |  |  |
|  | **Pathophysiological health** | Surgical procedures | **Location** | N/A |  |  |  |  |  |
| Cunningham et al. [49] | **Subjects** | 3 | **Signal type(s) used** | ABP (SBP), HR, respiratory rate, tcpO_2_ (sampled at 1 Hz, stored at 0.017 Hz) | This algorithm was designed to remove artifactual points in each of the recorded signals. This was accomplished using a simple filtering method. Any points that exceeded predetermined thresholds were excluded. Those for SBP were below 5 mmHg or above 250 mmHg.  **Validation:** Model was developed without training and was directly applied to a testing dataset  **Post-processing:** Rejection/removal of artifactual points | Three human observers manually removing artifacts from the monitored data based on their interpretation of an artifact, they likely had access to the recorded HR, respiratory rate, and tcpO_2_ | These were grouped into 6-hour blocks of data. The study was conducted across the first 7 days of life. The data was recorded in average values over a minute of recording. During each 6-hour window of data, a median and average value were calculated. The median and mean values were then recalculated for datasets that had been either manually or automatically scrubbed of artifacts.  The average median and mean difference were 1.15% and 1.48%, respectively. | There were significant differences between the raters as some removed only non-physiological artifacts and some included physiological changes due to medical intervention. The raters excluded 9.0%, 14.9%, and 7.9%, respectively.  Changes in SBP not clinically significant, but shows statistical impacts of screening data | Significant variations between observers  Quantification of results was inexact and did not provide much information as to the artifacts that were removed  Change in median or mean values is a poor method of description for effectiveness |
|  | **Sex (% Male)** | N/A |  |  |  |  |  |  |  |
|  | **Age** | Infants during first 7 days of life | **Device type** | N/A |  |  |  |  |  |
|  | **Pathophysiological health** | Low birth weight | **Location** | N/A |  |  |  |  |  |
| Tronstad et al. [50] | **Subjects** | 23 | **Signal type(s) used** | MAP (IBP and NIBP) (sampling rate equal to heart rate)  CO  Sampling rate equal to heart rate | The algorithm used for identifying and removing artifacts:  1. Artifacts identified based on threshold for deviation from median of the surrounding measurements  2. Artifacts identified as a value that deviates from the surrounding values of 75^th^ percentile and 25^th^ percentile  3. Conditional thresholding – if interbeat interval (IBI) is identified as artifactual based on previous two criteria, or being outside the expected range, the values at the same beat are also likely artifactual\  **Validation:** Model was developed without training and was directly applied to a testing dataset  **Post-processing:** Rejection/removal of artifactual points | Reference method proposed by Deegan et al [32]  Human evaluator, likely had access to the simultaneously recorded CO signal | Human evaluator treated as ‘gold-standard’  Using measurements from all recordings, artifact identification for CO using reference method:  LiDCO  Precision: 87%  Recall: 31%  F-Score: 46%  Nexfin  Precision: 76%  Recall: 28%  F-Score: 41%  Artifact identification for MAP using reference method:  LiDCO  Precision: 62%  Recall: 6%  F-Score: 10%  Nexfin  Precision: 58%  Recall: 6%  F-Score: 10%  Artifact identification for CO using proposed method:  LiDCO  Precision: 86%  Recall: 97%  F-Score: 91%  Nexfin  Precision: 79%  Recall: 89%  F-Score: 84%  Artifact identification for MAP using proposed method:  LiDCO  Precision: 79%  Recall: 94%  F-Score: 85%  Nexfin  Precision: 68%  Recall: 97%  F-Score: 80% | The proposed method has a stronger ability to remove true artifacts; however, it does erroneously remove true values inadvertently.  Proposed method has a higher F-score than the reference method (overall better method) | Proposed method removes portions of true signal  Small cohort, potentially single center |
|  | **Sex (% Male)** | 0% |  |  |  |  |  |  |  |
|  | **Age** | 34.3 ± 3.2 | **Device type** | LiDCO (invasive)  NexFin (non-invasive) |  |  |  |  |  |
|  | **Pathophysiological health** | Pregnant, caesarian delivery, spinal anaesthesia | **Location** | Forearm (LiDCO)  Finger (NexFin) |  |  |  |  |  |
| Deegan et al. [32] | **Subjects** | 20 | **Signal type(s) used** | NIBP (resampled at 10 Hz) | Artifacts were detected using the IQR of the 10 previous and future samples of a particular observation. Points labelled as artifacts were replaced using linear interpolation. The signal was then smoothed to 10 Hz using linear interpolation. A Butterworth filter based on work by Winter [134] was also applied with a cutoff frequency of 0.5 Hz.  **Validation:** Model was developed without training and was directly applied to a testing dataset  **Post-processing:** (identified above) artifactual points replaced using linear interpolation | Compared to clinician annotation  Low-pass filter with cutoff frequency of 0.2 Hz  7 point moving average filter | Success rate of the algorithm was 99.38% with 0.02% false positive rate  Low pass filter had success rate of 17.88% and the moving average filter removed 32.32% of artifacts. | Additional work was done to detect the displacement of patients when bed was tilted, not relevant to this review. | Sampling rate not listed, only resampling rate  Small, single center cohort with the same pathophysiology |
|  | **Sex (% Male)** | 45% |  |  |  |  |  |  |  |
|  | **Age** | 77.9 ± 11.65 | **Device type** | Finometer |  |  |  |  |  |
|  | **Pathophysiological health** | Orthostatic hypertensions | **Location** | Finger |  |  |  |  |  |
| Wilson et al. [51] | **Subjects** | 10 | **Signal type(s) used** | NIBP (indicated in excess of 16,000 samples in 4 hours ~ 1.11 Hz) | The basis of this algorithm was to adequately filter continuously measured NIBP signals such that SBP could be measured with similar accuracy to traditional NIBP methods. A median hybrid filter (MHF) was proposed which uses a central value resulting from several simultaneously operating linear sub-filters of varying window length. This particular MHF used 7 simple moving average linear sub-filters with progressively increasing window sizes, dictated by heart beats, that were derived using the Fibonacci series to ensure independence.  **Validation:** Model was developed without training and was directly applied to a testing dataset  **Post-processing:** Rejection/removal of artifactual points | Evaluated using cross-validation to NIBP collected using a brachial cuff collected before, and after dialysis by trained staff. | The MHF regression product from continuous SBP monitoring lines up well when overlaid compared to the discretely measured NIBP signal. There were 50 time-coincident NIBP measurements that had a mean difference of 0.95 mmHg ± 5.88 mmHg. This indicates strong results.  Evaluation was conducted Bland-Altman analysis.  The post-MHF signal integrity was validated using white noise regression periodogram analysis by metrics of Fisher’s kappa and Bartlett’s Kolmogorov–Smirnov method. They both indicate rejection of the null hypothesis of Gaussian noise exclusion testing | Bland-Altman analysis indicates that the proposed method was able to accurately model BP in dialysis patients.  Validated using Gaussian noise exclusion testing to confirm noise removal effectiveness.  This algorithm enables accurate continuous NIBP measurement, allowing for fluctuations in SBP to be better detected | Not broadly validated as it uses a small cohort  Not validated against ‘gold-standard’ invasive blood pressure monitoring device  Potential that filter could inadvertently remove drops in signal attributed to artifacts when it could be the result of another physiological process |
|  | **Sex (% Male)** | N/A |  |  |  |  |  |  |  |
|  | **Age** | N/A | **Device type** | Finometer MIDI (Finapres Medical Systems BV, Netherlands) |  |  |  |  |  |
|  | **Pathophysiological health** | Measured during intradialytic period | **Location** | Third finger of non-vascular access arm |  |  |  |  |  |

*ABP = Arterial Blood Pressure, BP = Blood Pressure, CO = Cardiac Output, DBP = Diastolic Blood Pressure, HF = High Frequency, HR = Heart Rate, IBI = Interbeat Interval, IBP = Invasive Blood Pressure, IQR = Interquartile Range, LF = Low Frequency, MAP = Mean Arterial Pressure, MHF = Median Hybrid Filter, mmHg = Millimeters of Mercury, N/A = Not Applicable, NIBP = Non-Invasive Blood Pressure, PPV = Positive Predictive Value, SBP = Systolic Blood Pressure, SD = Standard Deviation, tcpO₂ = Transcutaneous Oxygen Pressure*

**Table C.2. Low-frequency ABP artifact detection – machine learning-based methods**

| **Reference** | **Subject information** | | **Data type (sampling rate) – system type** | | **Artifact removal method(s) used** | **Methods compared** | **Effectiveness** | **Study results and conclusions** | **Limitations** |
| --- | --- | --- | --- | --- | --- | --- | --- | --- | --- |
| Wu et al. [52] | **Subjects** | 60 selected from 5,935 | **Signal type(s) used** | Systolic BP, MAP, and diastolic BP (IBP). Systolic BP, MAP, and diastolic BP (NIBP). HR measured using ECG and SpO2. SpO2 also measured. For all signals, every 15 seconds the median value for 8 readings was collected; 0.07 Hz) | Three types of algorithms applied:  1. Time series forecasting – applied state space and autoregressive moving average (ARIMA) models for forecasting. State space models involve exponential smoothing using a variety of different state space models. These include single exponential smoothing, double exponential smoothing (Holt Method) and triple exponential smoothing (Holt-Winters Method). The Holt-Winters method has an extension for seasonality (additive or multiplicative). An ARIMA-based forecasting framework was proposed by Box et al. which involves an iterative process involving model selection, parameter estimation, and model checking [135]  2. Standard Machine Learning Models – the Extreme Gradient Boosting model (XGBoost) developed by Chen and Guestrin was used [73]. The base model for this method is gradient boost trees, iteratively combining weak base learning models.  3. Anomaly Detection Algorithms – two algorithms selected included One Class Support Vector Machine (OCSVM) proposed by Scholkopf et al. [136] and Isolation Forest (IF) which was proposed by Liu et al [137]. The OSCVM algorithm is an extension of the Support Vector Machine (SVM) which is designed for imbalanced learning. The basis of the methodology is to find the hyperplane that separates a two-dimensional space (normal and anomalies). The IF algorithm attempts to isolate anomalies by randomly selecting a particular attribute and using this to partition the data.  Univariate strategies used only invasive systolic BP signal, multivariate strategies also used invasive diastolic and MAP. Univariate was conducted on the time series forecasting methods as well as the standard machine learning and anomaly detection algorithms. Multivariate was conducted on the standard machine learning and anomaly detection algorithms.  **Validation:** Data was split into 70% for training and testing, and 30% for validation. The bootstrapped residuals were used 100 times on the training and testing data. Algorithms were trained and optimized using different parameters.  **Post-processing:** None, only designed for artifact identification | Two anaesthesiologists manually annotated the dataset labelling the presence of artifacts. Each 15-second interval containing an artifactual point was labeled as an artifact. These reviewers were able to consider the NIBP, IBP, HR, SpO_2_, and ECG signals | Inter-rater agreement had an Krippendorff’s alpha coefficient across all cases was 0.966. When each case was considered in isolation, the average coefficient was 0.875.  The metrics that were used to evaluate the effectiveness of each algorithm included: precision (prec), recall (rec), specificity (spec), negative predictive rate (NPV), false detection rate (FDR) , geometric mean (G-Mean), and F1 score  The best performing method was the multivariate XGBoost method. Its scores were as follows:  F1 = 0.9997  G-Mean = 0.9998  Prec = 0.9998  Rec = 0.9997  Spec = 0.9999  NPV = 0.9999  FDR = 0.0002  XGBoost also had the best performance of the univariate strategies that were applied  F1 = 0.9953  G-Mean = 0.9964  Prec = 0.9974  Rec = 0.9933  Spec = 0.9996  NPV = 0.9990  FDR = 0.0026  ARIMA model generally performed the best of the time series methods during bootstrapping; however, there was less agreement across metrics during validation. | Standard machine learning method performed the best in artifact detection using both univariate and multivariate strategies | Potentially worse performance in the anomaly detection algorithms due to the number of ‘normal’ cases that were inputted.  Future work should involve extending multivariate strategies for more variables as well as leveraging resampling.  No mention of computational time performance  Not clear why other NIBP, SpO2, and ECG are mentioned as measured signals |
|  | **Sex (% Male)** | N/A |  |  |  |  |  |  |  |
|  | **Age** | Over 45 years | **Device type** | N/A |  |  |  |  |  |
|  | **Pathophysiological health** | Overnight surgery | **Location** | N/A |  |  |  |  |  |
| Tsien et al. (2000) [53] | **Subjects** | 123 | **Signal type(s) used** | IBP, HR via ECG, partial pressures of CO_2_ and O_2_ (0.0167 Hz) | This algorithm was developed to remove false instances of alarms in the measured bio-signals. Features of the time-series signal were extracted including the moving mean, median, standard deviation, linear regression line, the absolute value of the linear regression line, the maximum value, minimum value, and range. These were selected for window sizes of 3, 5, and 10 minutes. Each data point had been manually annotated as ‘artifactual’, ‘non-artifactual’, or ‘in-transition’, if the manual annotations indicated that more than half of the points in BP, CO_2_, or O_2_ windows were labelled as artifacts, the signal segment was labelled as an artifactual segment. Any artifacts in HR resulted in the entire segment being labelled as artifactual.  **Validation:** 70% of the data was used to train two models: C4.5 decision tree algorithm and logistic regression model optimized using threshold-based classification based on ROC curves. Each decision tree was trained for each vital signal respectively, resulting in each model having different numbers of nodes and features used to classify alarms. The remaining 30% of the data was then divided into evaluation for model optimization (9%) and testing (21%)  **Post-processing:** None, only designed for artifact identification | Signals manually annotated by physician with access to HR and partial pressure signals  Comparison between machine learning methods | Based on the test set data (21% of total data) with 130 HR, 78 BP, 183 CO_2_, and 64 O_2_ artifacts:  For decision tree model:  HR: Sens = 65.4%, Spec = 99.8%, AUCROC = 92.8%  BP: Sens = 57.7%, Spec = 99.9%, AUCROC = 89.4%  CO_2_: Sens = 82.5%, Spec = 99.2%, AUCROC = 93.3%  O_2_: Sens = 87.5%, Spec = 100%, AUCROC = 99.9%  For linear regression model:  Generally, performed much worse with AUCROC values for HR, BP, CO_2_, and O_2_ of 8.9%, 16.7%, 7.8%, and 26.8%, respectively. These were able to be slightly improved by dichotomizing variables like O_2_ into two. It increased the AUCROC to 86.0% for O_2_. | Decision tree model performed best at correctly classifying the false alarms  Multiple signal integration improved the performance for HR, BP, and CO_2_ alarms | Low sampling rate used  No optimization of window sizes  No mention as to how to deal with artifacts in the signal  Retrospective annotation of the data  Required 4 signals to be available |
|  | **Sex (% Male)** | N/A |  |  |  |  |  |  |  |
|  | **Age** | Neonatal | **Device type** | N/A |  |  |  |  |  |
|  | **Pathophysiological health** | Admitted to ICU | **Location** | N/A |  |  |  |  |  |
| Hravnek et al. [54] | **Subjects** | 634 (Block 1 = 308, Block 2 = 326) | **Signal type(s) used** | NIBP (SBP, DBP) (sampling rate not specified), HR, respiratory rate, SpO_2_ (0.05 Hz), as well as derived MAP and pulse pressure (PP) | The developed methodology implements machine learning techniques to differentiate true vital signal (VS) abnormalities or artifacts in individual categorized as at-risk for cardiorespiratory instability. The data was segmented into Block 1 (training and cross-validation) and Block 2 (testing) sets. Basic thresholds in VS amplitude and duration of time for each signal exceeding the amplitude were used to identify prospective cardiorespiratory instability events (812 in Block 1 and 1521 in Block 2). Block 1 data was used to determine specific rules to differentiate valid from artifactual signals. This was done using a two-part process:  1. Learning of features: three clinical investigators developed rules to differentiate the valid from artifactual signals. Such rules, specifically for BP signals, included an immediate drop in pulse pressure (difference between SBP and DBP), a rapid rise in SBP, MAP, and DBP, as well as repetitive readings. These rules and clinician judgement served as the basis of for the iterative classification of the VS segments. This iterative process resulted in 44 features being identified as the basis for artifact identification in the VS. These included statistical and spectral features of the signals.  2. Development of ML method: The proposed features were ranked using an AUC score generated using logistic regression between the features and labels. Features with AUC scores above certain thresholds (0.7 for BP) were used for classification. 5 features were used for blood pressure, 5 for RR, and 10 for SpO_2_. Several machine learning models were trained using these datasets including the K-nearest neighbor (KNN) at varying k values, linear discriminant analysis (LDA), naïve Bayesian classifier (NB), logistic regression (LR), support vector machine (SVM) with no specified kernel, and random forest (RF).  **Validation:** Training with 10-fold cross validation on Block 1, testing using Block 2  **Post-processing:** None, only designed for artifact identification | Two human experts classified the Block 1 and Block 2 datasets with access to the HR, respiratory rate, SpO_2_, as well as the derived MAP and PP | For Block 1,  RR had maximum AUC of 0.970 for RF and minimum AUC of 0.951 for KNN with k=5  BP had maximum AUC of 0.907 for LR and minimum AUC of 0.860 for NB  SpO_2_ had maximum AUC of 0.764 for NB and minimum AUC of 0.702 for KNN and SVM.  LR had most consistent performance across VSs with AUC scores for RR, BP and SpO2 of 0.956, 0.907, and 0.750, respectively.  For Block 2,  The model that performed the best between Block 1 and Block 2 was SVM | The RR artifacts were the easiest to identify and classify and the SpO_2_ artifacts were the most difficult  The method was generally successful in identifying artifacts in unseen offline data  This method has potential applicability in improving accuracy of VS alerts | Did not identify brief artifacts/outliers in the recorded signals, only when the artifacts persisted for several measurements.  Still remained some false positive and false negatives  Generalizability to higher sampling rate data  Single institution dataset |
|  | **Sex (% Male)** | Block 1 = 58.4%  Block 2 = 58.6% |  |  |  |  |  |  |  |
|  | **Age** | Block 1 = 57.42 ± 20.2  Block 2 = 57.92 ± 19.7 | **Device type** | N/A |  |  |  |  |  |
|  | **Pathophysiological health** | Admitted to SDU | **Location** | N/A |  |  |  |  |  |
| Pasma et al. [55] | **Subjects** | 88 for data at 0.017 Hz and 40 for data at 0.2 Hz | **Signal type(s) used** | IBP (0.2 Hz and 0.017 Hz) | Specific artifacts noted by human observers were in instances of: “flush, blood sampling, sensor issues or movement of the patient, simultaneous non-invasive blood pressure measurement and height of the pressure sensor.”  Artifacts were broken into four definitions:  1. Artifacts annotated by the observer in live time (used as reference)  2. Artifacts identified by the observer that lasted at minimum 30 seconds  3. Retrospectively identified artifacts  All of which were for IBP sampled at 0.017 Hz  4. For dataset at higher resolution (1/5 Hz) within a live observed dataset for data sampled at 0.2 Hz  Fed into three different machine learning algorithms: lasso penalized logistic regression, single layer neural network and a support vector machine method with radial basis  **Post-processing:** None, only designed for artifact identification | Live time observation of artifacts by human observer (with discussion with anaesthetist)  Retrospective annotation of artifacts by human observer | Parameters of machine learning algorithm were tuned using 4-fold cross validation (80%). Hyper parameters were chosen using Kappa statistic based on definitions. Tested using another set (20%).  Most robust set of artifacts was likely the retrospectively identified set. The top performing model was the SVM model with a Gaussian kernel with sensitivity, specificity, and PPV values of 0.521, 0.999, and 0.884, respectively. Using 1/5 Hz data, the results were similar achieving results for the same metrics of 0.518, 0.997, and 0.830. | Performance increased using retrospectively annotated artifacts, but overall performance was poor and deemed unfit to be used in place of human observers. | Insufficient data for construction of a machine learning based method |
|  | **Sex (% Male)** | 44% |  |  |  |  |  |  |  |
|  | **Age** | 66 (56-74) | **Device type** | N/A |  |  |  |  |  |
|  | **Pathophysiological health** | Surgical procedures | **Location** | N/A |  |  |  |  |  |
| Tsien et al. (2001) [56] | **Subjects** | 100+ (Group 1)  2 (Group 2) | **Signal type(s) used** | IBP, HR via ECG, partial pressure of CO_2_ and O_2_  Group 1 at 0.0167 Hz  Group 2 at 1 Hz | This algorithm was designed to identify artifacts in data streams recorded in the ICU. Features extracted from the signals include moving mean average, median value, maximum value, maximum value, minimum value, range, standard deviation, linear regression slope, and absolute value of the linear regression slope. In the analysis of this method, different window sizes were evaluated. There were two experiments conducted. The first calculates features based on 180, 360, and 600 values (corresponding to 1-minute, 3-minute, and 5-minute). This allows for the model developed using the 0.0167 Hz model to the 1 Hz model to be directly compared. The second experiment uses 3, 5, and 10 values. This model was then applied to both experiments data.  **Validation:** The first experiment data was split into a 70%-9%-21% training-evaluation-test split and was used as an input to the C4.5 decision tree induction model [74]  **Post-processing:** None, only designed for artifact identification | Manual annotations of artifacts by experienced clinician with access to the simultaneously recorded HR and partial pressure signals | AUROC curves were generated between the sensitivity and specificity of the models for BP, CO_2_, O_2_, and HR. The results for the BP signals are indicated below.  For 0.01617 Hz model used for 0.0167 Hz test set: AUCROC = 89.41%  For 1 Hz model used for 1 Hz test set: AUCROC = 100.00%  For 0.01617 Hz model used for 1 Hz test set from the first experiment: AUCROC = 100.00%  For 0.01617 Hz model used for 1 Hz test set from the second experiment: AUCROC = 100.00% | The 0.0167 Hz model still functions on the 1 Hz model, which requires less data | Small dataset of 1 Hz data  Single reviewer for artifacts indicates potential bias  Retrospective analysis without mention of real-time use  Limited number of window sizes tested used for each signal |
|  | **Sex (% Male)** | N/A |  |  |  |  |  |  |  |
|  | **Age** | Neonates | **Device type** | N/A |  |  |  |  |  |
|  | **Pathophysiological health** | ICU | **Location** | N/A |  |  |  |  |  |
| Sebald [57] | **Subjects** | 1 | **Signal type(s) used** | IBP (20 Hz) | Presents two different neural network-based methods  1. Back propagation topology – there was an input layer, hidden layer, and single output layer. Optimal size of the hidden layer was 21 nodes. The output was then converted to a binary classification using a simple threshold.  2. Competitive learning topology – uses a ‘winner take all approach’, for an input pattern, the largest output is the ‘winner’. Input weights are then adjusted. Optimal output node number was the largest tested (n=21).  **Validation:** Each model was trained on one group and tested on the other group  **Post-processing:** None, only designed for artifact identification | Manual characterization by experts as either artifacts or non-artifacts | For back propagation method, networks were trained using 1,000 epochs, processing all 66 patterns in Group II. The network performed perfectly on new data from Group I with threshold applied to output (0% error)  For the competitive learning method, the method was trained on Group I and tested on Group I (57 patterns) and Group II (66 patterns) without retraining, it achieved a 7% error for Group I and a 6% error for Group II. | The back propagation method outperformed the competitive learning method  The back propagation method was able to identify an artifact mislabelled by expert  Competitive learning method was faster and simpler | The back propagation method got stuck on local minima, slowing it slightly  Issues for competitive learning algorithm with accuracy  Competitive learning topology could not be tuned/optimized like the back propagation  Trained and tested on single patient data |
|  | **Sex (% Male)** | N/A |  |  |  |  |  |  |  |
|  | **Age** | N/A | **Device type** | Catheter |  |  |  |  |  |
|  | **Pathophysiological health** | About to undergo cardiac surgery | **Location** | Artery |  |  |  |  |  |
| Haule et al. [58] | **Subjects** | 64 of 125 from KidsBrain IT dataset [59], patient from MIMIC-IV [138] | **Signal type(s) used** | ABP (mean), ICP (mean), HR  Sampling rate only mentioned for MIMIV-IV set where it was downsampled from 256 Hz to 0.017 Hz | The aim of this algorithm was to develop a method for artifact identification in an ICU setting that was completely unsupervised. This algorithm makes use of the *β*-VAE and IF. This model involves the initial training of a VAE model, specifically the *β*-VAE [139] using both raw and cleaned datasets. In this architecture, there are stacked LSTM layers and a dense layer in both the encoder and decoder, with the encoder also having an attention layer. This model is optimized through training to tune hyperparameters using Bayesian optimization. All of the vital signals measured (ABP, ICP, and HR) were used in the training of the model. Features are extracted from the signals and used as the input for the IF algorithm [137] for classification as either ‘clean’ or ‘artifactual’.  Benchmark models were developed such that this novel model could be compared to common frameworks, these included an LSTM model with self-attenuation [140], an XGBoost model [73], and an ARIMA model [141]. These were developed for comparative artifact identification.  **Validation:** Training and testing were conducted using a split of 47 and 17 patients (on a patient-by-patient basis)  **Post-processing:** None, only designed for artifact identification | Manual annotation assigning binary ‘clean’ or ‘artifactual’ labels, likely had access to simultaneously recorded ICP and HR signals | For the VAE-IF method, it was able to achieve mean values on the KidsBrainIT dataset of:  Sens = 81.0%  Spec =88.4%  AUCROC = 84.7%  This method had the highest sensitivity. The ARIMA model outperformed by specificity, achieving a value of 99.3%. The LSTM model outperformed it in AUCROC achieving a value of 89.0%. However, its strong performance despite being unsupervised is notable | Strong performance despite being unsupervised.  Performed generally strongly across metrics  A plot was shown indicating that this algorithm could be used on a different dataset to identify artifacts; however, success was not quantified | External validation was limited  Lower specificity compared to other methods  Training involves auxiliary signals |
|  | **Sex (% Male)** | N/A |  |  |  |  |  |  |  |
|  | **Age** | Pediatric | **Device type** | N/A |  |  |  |  |  |
|  | **Pathophysiological health** | Admitted to PICU (multiple centres) | **Location** | N/A |  |  |  |  |  |

*ABP = Arterial Blood Pressure, AUC = Area Under the Curve, AUCROC = Area Under the Receiver Operating Characteristic Curve, ARIMA = Autoregressive Integrated Moving Average, AuBP = Auscultatory Blood Pressure, BP = Blood Pressure, CO = Cardiac Output, DBP = Diastolic Blood Pressure, ECG = Electrocardiogram, F1 = F1 Score, FDR = False Detection Rate, G-Mean = Geometric Mean, HR = Heart Rate, IBP = Invasive Blood Pressure, ICU = Intensive Care Unit, IF = Isolation Forest, KNN = K-Nearest Neighbour, LDA = Linear Discriminant Analysis, LR = Logistic Regression, LSTM = Long Short-Term Memory, MAP = Mean Arterial Pressure, NB = Naïve Bayes, NIBP = Non-Invasive Blood Pressure, NNET = Neural Network, NPV = Negative Predictive Value, OCSVM = One-Class Support Vector Machine, PP = Pulse Pressure, Prec = Precision, Rec = Recall, RF = Random Forest, ROC = Receiver Operating Characteristic, RR = Respiratory Rate, SD = Standard Deviation, Sens = Sensitivity, Spec = Specificity, SpO₂ = Pulse Oximetry, SVM = Support Vector Machine, β-VAE = Variational Autoencoder with β hyperparameter, VAE = Variational Autoencoder, VS = Vital Sign, XGBoost = Extreme Gradient Boosting*

**Table C.3. Low-frequency ABP artifact detection – statistical modeling-based methods**

| **Reference** | **Subject information** | | **Data type (sampling rate) – system type** | | **Artifact removal method(s) used** | **Methods compared** | **Effectiveness** | **Study results and conclusions** | **Limitations** |
| --- | --- | --- | --- | --- | --- | --- | --- | --- | --- |
| Lin et al. [60] | **Subjects** | 47 | **Signal type(s) used** | NIBP | The basis of the device and corresponding algorithm was to smooth the ABP signal and detect the systolic and diastolic pressures in the presence of signal disturbances in oscillatory amplitude (OA). The inputs to the algorithm are the pulse cuff pressure, the OAs, the angle between three consecutive OAs (θ_1_) and five consecutive OAs (θ_2_). These can be used to detect irregularities in the signal. A weight parameter from the fuzzy logic discriminator (FLD) is determined using the OAs, θ_1_, and θ_2_, assigning larger weights to OAs that likely exemplify true signals. The Recursive Weighted Regression Algorithm (RWRA) takes inputs of the cuff pressure, OAs, and the resulting weight from the FLD and uses these values to reconstruct the shape of change in OA over cuff pressure. The systolic and diastolic pressures can then be determined.  **Post-processing:** None, only designed to smooth artifacts  **Validation:** Model was developed without training and was directly applied to a testing dataset | Gold-standard was auscultatory blood pressure recorded manually  Compared to a traditional curve fitting algorithm (TCFA) | One patient had data rejected due to the number of disturbances.  For systolic measurement, there was a strong results comparing the results of the auscultatory technique and the oscillatory method with the RWRA method. It had a correlation coefficient (R) of 0.98, standard error of estimate (SEE) of 4.7 mmHg. For TCFA combined with the oscillatory method, the results were slightly worse with R = 0.97 and SEE = 4.9 mmHg. This was further indicated by t-test results. For diastolic measurement, the RWRA method was slightly less accurate than the TCFA by t-test results; however, in diastolic measurement, both algorithms achieved R = 0.99, and the accuracy was better in the RWRA method (SEE = 4.9 mmHg compared to 5.1 mmHg). | RWRA is robust in rejecting interference by artificial motion or arrhythmic disturbance  It had slightly superior performance to the TCFA  Effectively reduced the effects of disturbances | No external validation of algorithm  Conducted on subjects with only particular cardiovascular issues  Small sample size  System was calibrated |
|  | **Sex (% Male)** | 72.3% |  |  |  |  |  |  |  |
|  | **Age** | 61 ± 15 | **Device type** | Mercury column sphygmomanometer (method 1) and designed calibration oscillometric system (method 2) |  |  |  |  |  |
|  | **Pathophysiological health** | 38 with cardiovascular abnormalities, 9 healthy | **Location** | Cuff on upper arm, measured at brachial artery |  |  |  |  |  |

*ABP = Arterial Blood Pressure, ACF = Autocorrelation Function, AR = Autoregressive, AR(2) = Second-Order Autoregressive Model, CI = Confidence Interval, FLD = Fuzzy Logic Discriminator, NIBP = Non-Invasive Blood Pressure, OA = Oscillatory Amplitude, PS = Phase Space, R = Correlation Coefficient, RWRA = Recursive Weighted Regression Algorithm, SEE = Standard Error of Estimate, TCFA = Traditional Curve Fitting Algorithm*

**Table C.4. Low-frequency ABP artifact detection – signal quality-based methods**

| **Reference** | **Subject information** | | **Data type (sampling rate) – system type** | | **Artifact removal method(s) used** | **Methods compared** | **Effectiveness** | **Study results and conclusions** | **Limitations** |
| --- | --- | --- | --- | --- | --- | --- | --- | --- | --- |
| Abdul Sukor et al. (2015) [61] | **Subjects** | 4 | **Signal type(s) used** | Self-administered NIBP (measured 3 times per day at home), AuBP, ECG, assumed to follow the same recording methodology as previous study by authors [87] | Similar to the previous work [87], the signal quality was determined by the accurate estimation of the diastolic and systolic blood pressures from contaminated BP signals. The previous algorithm involves:  The proposed algorithm was designed to classify noise within the NIBP signal and estimate SBP and DBP. This involves:  1. Pre-processing – this involved cuff-pressure being down sampled to 100 Hz, applying sixth order Butterworth filter between 0.5 and 5 Hz (based on maximum heart rate). Signal was then segmented using a maximum filter and estimating the minimum within the peak-to-peak interval.  2. Noise classification – this was first conducted using morphology. Interruptions in the gradual decrease indicated a disruption in cuff airflow and an acoustic artifact in the NIBP signal using difference between cuff pressure and filtered cuff pressure. Second method conducted based on Korotkoff pulse width  3. Systolic and diastolic estimation feasibility – systolic point is identified as the time of the first two Korotkoff sounds within a time span less than twice the mean beat-to-beat interval. Diastolic point is identified as either (1) the time since the last Korotkoff pulse exceeds twice the mean beat-to-beat interval or (2) the diastolic point is identified as the trough in the oscillometric signal immediately followed by a Korotkoff pulse. These are only considered valid if a detected noise section is further than two mean beat-to-beat intervals from the event.  **Validation:** Model was developed without training and was directly applied to a testing dataset  **Post-processing:** No interpolation or reconstruction applied | Similar to previous study conducted [87], the reference standard was developed by two human raters that identified noise with access to simultaneously recorded AuBP and ECG signals | There were 120 pooled signals for BP and 75% of actual noisy signals were correctly classified, and 97% and 91% of signals from which systolic and diastolic points could be estimated.  These points were able to be identified with a mean error and standard deviation of 2.53 ± 4.20 mmHg and 1.46 ± 5.29 mmHg compared to the reference standard. | Algorithm was able to reliably detect noisy signals and well estimate when the systolic and diastolic pressures could be estimated.  Reasonably accurate detection of temporal locations of systolic and diastolic points  This is potentially applicable in telehealth applications | Small cohort from similar age  Trainer explained operation of protocol for measurement |
|  | **Sex (% Male)** | 50% |  |  |  |  |  |  |  |
|  | **Age** | 63.3 (59 to 67) | **Device type** | Cuff |  |  |  |  |  |
|  | **Pathophysiological health** | Healthy, over 50 | **Location** | Upper arm |  |  |  |  |  |
| Fanelli and Heldt [62] | **Subjects** | 11 (Group 1) and 37 (Group 2) | **Signal type(s) used** | IBP, ICP, NIBP (Group 1: 50 Hz, Group 2: 40 to 70 Hz) | Signal quality and waveform reconstruction is conducted through four steps:  1. Pre-processing – involves low-pass filtering (16 Hz), upsampling signal using linear interpolation to 120 Hz, and detection of ABP beat onsets using the Zong et al. method [15].  2. Beat onset correction – involves the correction of beats identified using the Zong et al. approach [15]. This assumes that the *i*-th detected onset is correct and examines the possible 10 detections provided by the Zong et al. algorithm to predict the most reasonable i+1th pulse onsets. This involves initially calculating the inter-beat interval of the previous 5 correct onsets. This value is used as a template and is compared to 10 future detected pulses. That which most closely aligns with the identified inter-beat interval is used as the ‘correct’ *i+1*th onset for comparison.  3. Quality computation – waveform quality is assessed on an individual pulse level. The mean difference between successive ABP pulse amplitudes. This value is then normalized by comparing it to the mean of the previous 20 values. A threshold was used to determine whether or not the *mean* value indicated a good or poor quality waveform.  4. Signal reconstruction – this involves first computing the average duration of all good quality beats within a 60 beat window, noisy beats are divided by the duration to determine number of noisy beats, average of all good quality wavelets in the same window are used to reconstruct the beats and wavelets are transformed to meet size of average duration, interpolation is used (piecewise cubic Hermite interpolation for diastolic and Fourier series interpolation for systolic). Reconstructed signals systolic and diastolic values match the envelope function.  **Validation:** Model was developed without training and was directly applied to a testing dataset  **Post-processing: (**identified above) reconstruction of the artifactual signal was completed using a complex interpolation method outlined above | Manual annotation by two experts who inspected 14 ABP waveform records were randomly selected from first dataset and 4 from second datasets. | Compared to manual annotation, the overall accuracy was 99%, sensitivity was 95%, and specificity was 99% in identifying noisy segments.  The inter-rater accuracy was 99%, sensitivity was 99%, and specificity was 99%.  For the abilities of the wavelet reconstruction algorithm, the good signal quality segments were reconstructed to compare to actually noisy segments that had been reconstructed.  Mean squared error was calculated between reconstructed ABP wavelets and original ones for 20 windows that ranged from 1 to 10 beats. For largest of these windows, the average mean square error was below 1 mmHg, indicating strong results. Absolute pulse pressure was below 5 mmHg for 10-beat reconstruction. | Strong performance in identifying noisy signal segments  Reconstructed ABP wavelets were very similar to the non-reconstructed ones.  ** wavelets in this context are the beat waveforms. | Assumes that all beats within a noisy segment are the same length  Assumes beat shapes are the same |
|  | **Sex (% Male)** | N/A |  |  |  |  |  |  |  |
|  | **Age** | N/A | **Device type** | Catheter and finger cuff |  |  |  |  |  |
|  | **Pathophysiological health** | ICU, based on ICP signal having been recorded | **Location** | Radial artery and Finapres |  |  |  |  |  |
|  | **Pathophysiological health** | Obese | **Location** | Radial (ABP) and forearm (NIBP) |  |  |  |  |  |

*ABP = Arterial Blood Pressure, AuBP = Auscultatory Blood Pressure, BP = Blood Pressure, DBP = Diastolic Blood Pressure, ECG = Electrocardiogram, IBP = Invasive Blood Pressure, ICP = Intracranial Pressure, NIBP = Non-Invasive Blood Pressure, SBP = Systolic Blood Pressure, SD = Standard Deviation*

**Table C.5. Low-frequency ABP artifact detection – motion-based methods**

| **Reference** | **Subject information** | | **Data type (sampling rate) – system type** | | **Artifact removal method(s) used** | **Methods compared** | **Effectiveness** | **Study results and conclusions** | **Limitations** |
| --- | --- | --- | --- | --- | --- | --- | --- | --- | --- |
| Muroi et al. [63] | **Subjects** | 45 | **Signal type(s) used** | IBP (SBP, MAP) (1 Hz), SpO_2_, ICP, and HR, cameras for motion | AXIS network cameras were installed over beds to monitor patient movement  An optical flow algorithm was used to determine the displacement or movement of the patient  Thresholds were established for alarms for both the values of the physiological signals but also in the calculated motion vectors  **Validation:** Model was developed without training and was directly applied to a testing dataset  **Post-processing:** None, only designed for artifact identification | Neurocritical care specialist conducted a retrospective analysis of vital signal alarms with no access to the video, artifact classification included: if signal curve was not visible, if curve had a non-physiological shape, or if the numerical value was unrealistic (labels them as green, orange or red), likely had access to the simultaneously recorded SpO_2_, ICP, and HR signals | For the systolic arterial blood pressure, alarms (n=1063) were detected at:  RED (true alarm): sensitivity = 87.1%, specificity=36.5%, PPV=81.5%  GREEN (false alarm): sensitivity = 38.4%, specificity=86.7%, PPV=38.6%  For the mean arterial blood pressure, alarms were detected at:  RED:  sensitivity = 76.2%, specificity=35.0%, PPV=53.7%  GREEN:  sensitivity =35.6%, specificity=78.6%, PPV=54.3%  Overall, this classification performed better for the systolic ABP than mean ABP  No data for the successful identification of ORANGE alarms (possible false alarms without enough data to verify) | In general, there was a poor performance in terms of sensitivity, specificity, and PPV, despite these values being higher for RED alarms compared to GREEN.  RED alarms are ultimately the most important | High false positive rate  Over all signals, a 13% false negative rate  Too many false alarms to be implemented in ICU, need more accurate classification  Current analysis does not include the risk of misclassification of alarms |
|  | **Sex (% Male)** | 40% |  |  |  |  |  |  |  |
|  | **Age** | 59.6 ± 14.9 | **Device type** | Arterial line |  |  |  |  |  |
|  | **Pathophysiological health** | Aneurysmal SAH, intracerebral hemorrhage, other | **Location** | N/A |  |  |  |  |  |

*ABP = Arterial Blood Pressure, HR = Heart Rate, IBP = Invasive Blood Pressure, ICP = Intracranial Pressure, MAP = Mean Arterial Pressure, PPV = Positive Predictive Value, SAH = Subarachnoid Hemorrhage, SBP = Systolic Blood Pressure, SpO₂ = Pulse Oximetry*

**Table C.6. Low-frequency ABP artifact detection – articles presenting multiple methods and applying previously developed methods**

| **Reference** | **Subject information** | | **Data type (sampling rate) – system type** | | **Artifact removal method(s) used** | **Methods compared** | **Effectiveness** | **Study results and conclusions** | **Limitations** |
| --- | --- | --- | --- | --- | --- | --- | --- | --- | --- |
| Maleczek et al. [64] | **Subjects** | 106 | **Signal type(s) used** | NIBP  IBP (0.067 Hz in OR, 0.0011 in ICU), HR, temperature, SpO_2_, and etCO_2_ | Five methods compared:  1. Cutoff - thresholds for artifacts defined using physiological values (for mean BP: 10 to 250 mmHg)  2. Z-value– z-value calculated, values outside three multiples of SD were marked as artifacts (all values outside of 99.73% of mean excluded)  3. Interquartile range (IQR) – values outside of three times the IQR marked as artifacts (all values outside of 99.73% of median excluded)  4. Local outlier factor – methodology described by Breunig et al. to detect dramatic changes in the data, local factors greater than 1.5 were labeled as artifacts [65].  5. LSTM neural network – algorithm was trained using human reference. Dataset randomly split between training (80%) and test (20%)  **Validation:** Models 1-4 were developed without training and was directly applied to a testing dataset, model 5 was trained using 80% of the data and tested on 20%.  **Post-processing:** None, only designed for artifact identification | Intercomparison of the methods was compared against manual review by two experts, they had access to simultaneously recorded HR, temperature, SpO_2_, and etCO_2_ signals | For invasive MAP:  **Sensitivity (95% CI):**  1. Cutoff - 74.9 (70.3–79.0)  2. LSTM - 54.4 (38.4–56.7)  3. IQR - 47.1 (42.2–52.1)  4. Z-value - 46.1 (41.1–51.1)  5. Local outlier factor - 14.1 (11.0–18.0)  **Specificity (95% CI):**  1. Local outlier factor - 100 (100.0–100.0)  2. Cutoff - 100 (100.0–100.0)  3. LSTM - 99.8 (99.7–99.9)  4. Z-value - 99.4 (99.3–99.5)  5. IQR - 98.9 (98.7–99.0)  **PPV (95% CI):**  1. Cutoff - 99.7 (98.1–99.9)  2. Local outlier value - 96.4 (87.9–99.0)  3. LSTM - 75.6 (69.8–88.7)  4. Z-value - 66.7 (60.8–72.1)  5. IQR - 51.7 (46.5–56.9)  **NPV (95% CI)**  1. Cutoff - 99.4 (99.2–99.5)  2. LSTM - 99.4 (99.1–99.4)  3. Z-value - 98.6 (98.4–98.8)  4. IQR - 98.7 (98.5–98.8)  5. Local outlier factor - 97.9 (97.6–98.1)  For non-invasive MAP:  **Sensitivity (95% CI):**  1. IQR - 42.9 (15.8–75.0)  2. Z-value - 42.9 (15.8–75.0)  3. Local outlier factor - 0 (0.0–35.4)  4. Cutoff - 0 (0.0–35.4)  5. LSTM – N/A  **Specificity (95% CI):**  1. Cutoff - 100 (99.9–100.0)  2. Local outlier factor - 99.9 (99.7–100.0)  3. Z-value - 99.2 (98.8–99.4)  4. IQR - 99 (98.6–99.3)  5. LSTM – N/A  **PPV (95% CI):**  1. IQR - 9.4 (3.2–24.2)  2. Z-value - 10.7 (3.7–27.2)  3. local outlier factor - 0 (0.0–56.1)  4. Cutoff – N/A  5. LSTM – N/A  **NPV (95% CI):**  1. IQR - 99.9 (99.7–99.9)  2. Z-value - 99.9 (99.7–99.9)  3. Local outlier factor - 99.8 (99.5–99.9)  4. Cutoff - 99.8 (99.5–99.9)  5. LSTM – N/A | Significant differences between the number of artifacts “annotated” by each method  Non-invasive was predominantly conducted on OR patients, invasive was predominantly conducted on ICU patients. There was differing success in the algorithms based on the datasets used. | Retrospective annotation of the datasets  Small rate of artifacts by human observers  Limitations to neural networks  Human error (minimized by using 2 observers + 1 to decide disagreements)  Single center dataset  No defined pathophysiology of subjects |
|  | **Sex (% Male)** | 62.3% for OR  60.4% for ICU |  |  |  |  |  |  |  |
|  | **Age (quartile)** | 59.0 (42.0, 68.0) for OR  60.0 (49.0, 67.0) for ICU | **Device type** | Cuff (NIBP)  Arterial line, (IBP) |  |  |  |  |  |
|  | **Pathophysiological health** | 53 admitted to ICU  53 admitted to OR | **Location** | Mainly radial artery (IBP) |  |  |  |  |  |
| Verduijn et al. [67] | **Subjects** | 18 of 367 | **Signal type(s) used** | MAP (0.017 Hz) (presumed to be IBP based on application), central venous pressure (CVP) and HR | Applied three methods depicted in literature. The first was a moving median filtering method. The second was the ArtiDetect method proposed by Cao et al. [47]. The third method was the multiple signal integration by tree induction method proposed by Tsien et al. [53]  These methods were trained and validated in several ways when compared to expert judgements. These included:  1. Individual internal validity – where a 10-fold cross-validation was conducted on artifacts labeled by one expert  2. Individual ‘external’ validity – the methods were trained using annotations from one expert and tested against the judgements of the other three.  3. Joint majority vote validity – the methods were developed using a 10-fold cross-validation of the joint decisions for artifact labeling. A ‘majority rules’ method was used when there were disagreements.  4. Joint consensus validity – the methods were validated using a 10-fold cross-validation where all disagreements were formally discussed.  In the effectiveness column, the joint consensus method was used as it is the most robust and had the best performance  **Validation:** Model was trained and cross-validated using a 10-fold cross-validation method  **Post-processing:** None, only designed for artifact identification | Expert judgements either individually, majority rules (3 of 4, or consensus, likely had access to HR and CVP signals | A 10-fold cross validation was conducted for MAP using joint consensus of artifact labeling  Median filtering:  Sensitivity = 0.67 (0.47 –0.83)  PPV = 0.91 (0.71–0.99)  ArtiDetect:  Sensitivity = 0.77 (0.58–0.90)  PPV = 0.64 (0.46–0.79)  Tree induction:  Sensitivity = 0.60 (0.41–0.77)  PPV = 0.69 (0.48–0.86)  Based on the type of validation that was used, different methods performed differently. For MAP specifically, the best methods for each validation type were:  For (1): ArtiDetect  For (2): ArtiDetect  For (3): Tree induction  For (4): ArtiDetect | There was a considerably worse performance when the methods were trained using individual judgements of artifacts, this indicates the implications of solely relying on a single source for artifact classification  Disagreement as to the best method based on the method of training/validation that was used | Single center, small cohort  Consensus process did not follow a formal technique |
|  | **Sex (% Male)** | N/A |  |  |  |  |  |  |  |
|  | **Age** | N/A | **Device type** | N/A |  |  |  |  |  |
|  | **Pathophysiological health** | Cardiac surgery (post-operation) | **Location** |  |  |  |  |  |  |
| Khan et al. [68] | **Subjects** | 15 | **Signal type(s) used** | IBP (SBP, DBP, and MAP were derived from signals at 240 Hz sampled at 0.5 Hz) | Implements the algorithm proposed by Du et al. [48]. The temporal distance between consecutive measurements (δ) illustrated in the aforementioned algorithm for MAP, SBP, and DBP were 6, 8, and 5 mmHg, respectively. Values outside of this indicate artifacts. These values were optimised in this article for the critically ill patients included. Additionally, there were absolute physiological limits for MAP, SBP, and DBP that were described using literature values (that values fell between 30 mmHg and 200 mmHg). Data gaps were not filled. This algorithm was compared to annotated signal by varying the δ values, eventually optimizing it, and including/excluding the absolute physiological values.  **Validation:** Model was developed without training and was directly applied to a testing dataset  **Post-processing:** The values detected as artifactual were interpolated based either on the other two blood pressure readings or using the processing non-artifactual points. If there was insufficient data for interpolation, the point was simply removed. | Manual analysis by a trained individual examining the MAP signal for segments of non-physiolo-gical origin, reviewed by a second individual | With accuracy optimized and no upper/lower thresholds used:  Optimal multiplication factor for δ was 0.4, sens = 94.96%, spec = 98.02%, MCC = 0.34  With MCC optimized and no upper/lower thresholds used:  Optimal multiplication factor for δ was 1.3, sens = 93.28%, spec = 98.68%, MCC = 0.40  With accuracy optimized and upper/lower thresholds used:  Optimal multiplication factor for δ was 1.3, sens = 97.48%, spec = 98.68%, MCC = 0.41  With MCC optimized and upper/lower thresholds used:  Optimal multiplication factor for δ was 2, sens = 96.64%, spec = 98.72%, MCC = 0.42.  The addition of thresholds seemed to reduce errors.  Using a δ value of 1.3 (optimized), 14% were removed outright and 86% were attempted to be interpolated using based on SBP and DBP data. Of those, 88% were false positives and 12% were true positives. Tends to be cautious. | High performance in identification of artifactual segments using optimized δ values  Algorithm attempts to repair erroneous data using SBP and DBP values  Not reliant on full waveform data  Trained and tested on critically ill patients from multiple centers | High number of false positives leading to a lower MCC  Seemed to be tested on the same dataset that it was trained on  No quantification for the interpolation of artifactual points |
|  | **Sex (% Male)** | N/A |  |  |  |  |  |  |  |
|  | **Age** | N/A | **Device type** | Arterial line (GE Solar Monitors) |  |  |  |  |  |
|  | **Pathophysiological health** | Critically ill from multiple centers | **Location** | N/A |  |  |  |  |  |
| Kuhrn et al. [66] | **Subjects** | 106 | **Signal type(s) used** | IBP/NIBP, oxygen saturation, etCO_2_, temperature, and pulse | There were four algorithms applied:   1. IQR-based: artifacts are identified as values that are 3 times the IQR based on first and third quartiles 2. Z-score-based: artifacts are those three standard deviations from the mean value 3. Cut-off: based on physiological values 4. Moving mean and median values: calculated moving mean and median values based on past 10 values   However, results were not displayed for 4  **Validation:** Model was developed without training and was directly applied to a testing dataset  **Post-processing:** None, only designed for artifact identification | Manual annotation by two reviewers, likely with access to simultaneously recorded oxygen saturation, etCO_2_, temperature, and pulse | Effectiveness was quantified using sensitivity and precision. It was calculated separately for IBP and NIBP, as well as calculated for the setting in which the signals were being measured (OR or ICU).  The method with the overarching best performance was the Z-score-based method which achieved a total sensitivity of 0.37 and a precision of 0.38. | Identified the Z-score method using a robust and sizeable dataset.  The article also included the percentage of the recording that were identified as artifact by each method | Poor performance according to both sensitivity and precision, even of the best performing method. |
|  | **Sex (% Male)** | N/A |  |  |  |  |  |  |  |
|  | **Age** | N/A | **Device type** | N/A |  |  |  |  |  |
|  | **Pathophysiological health** | 53 OR, 53 ICU | **Location** | N/A |  |  |  |  |  |
| Cretu et al. [69] | **Subjects** | 16 | **Signal type(s) used** | IBP (measuring SBP), CVP | This article presented two methods for baseline filtering and applied them to the ABP signal. These methods included:   1. Asymmetric Least Squares Smoothing (ALS) [70] – a smoothing method that initially models the signal, accurately constructing and smoothing the signal by minimizing the penalized least squares function by tuning parameters. Used to reconstruct the signal as a smoothed version. 2. Discrete Wavelet Transformation (DTW) [71] – which used a mean-median filter to obtain an estimation of baseline wander (low-frequency) followed by decomposition using the discrete wavelet transformation. The use of the t-test was used to reconstruct the signal to reduce the effect of baseline wander. This was initially proposed for ECG signals.   **Validation:** Model was developed without training and was directly applied to a testing dataset  **Post-processing:** None, only designed for baseline noise removal | Internal comparison of the SNR | The ALS algorithm was able to increase the SNR by 6% compared to the unfiltered signal  The DTW algorithm increased the SNR by 27%. | DTW algorithm provided better smoothing to reduce artifacts in ABP signal, improving negative correlation with CVP when filtered for noise correction | SNR was the only metric for effectiveness of the removal of noise  Lacking gold-standard  Heartbeat manipulated using the AV delay settings, not necessarily representative of BP signal of general population |
|  | **Sex (% Male)** | 75% |  |  |  |  |  |  |  |
|  | **Age** | 71 (41 to 80) | **Device type** | Edwards Lifesciences TruWave pressure transducer |  |  |  |  |  |
|  | **Pathophysiological health** | Post-cardiac surgery, changes in atrioventricular delay of varying increments via temporary pacemakers | **Location** | Radial artery |  |  |  |  |  |

*ABP = Arterial Blood Pressure, ALS = Asymmetric Least Squares Smoothing, CI = Confidence Interval, CVP = Central Venous Pressure, DBP = Diastolic Blood Pressure, DTW = Discrete Wavelet Transformation, etCO_2_ = End-Tidal Carbon Dioxide IBP = Invasive Blood Pressure, ICU = Intensive Care Unit, IQR = Interquartile Range, LSTM = Long Short-Term Memory, MAP = Mean Arterial Pressure, MCC = Matthews Correlation Coefficient, NIBP = Non-Invasive Blood Pressure, NPV = Negative Predictive Value, OR = Operating Room, PPV = Positive Predictive Value, SBP = Systolic Blood Pressure, SD = Standard Deviation*

1. **Supplementary File 4**

**Table D.1. High-frequency ABP artifact detection – machine learning-based methods**

| **Reference** | **Subject information** | | **Data type (sampling rate) – system type** | | **Artifact removal method(s) used** | **Methods compared** | **Effectiveness** | **Study results and conclusions** | **Limitations** |
| --- | --- | --- | --- | --- | --- | --- | --- | --- | --- |
| Kim et al. [36] | **Subjects** | 30 of 99 | **Signal type(s) used** | IBP | Preprocessing: manual experts detected systolic peaks, estimated pulse onsets, and identified pulses described in previous work [142]. Pulse length and SBP/DBP calculated from segmented pulses, which were then interpolated using cubic spline. These were then normalized for probabilistic representation to be inputted.  Deep belief network (DBN): 6 layers including: a visual layer with 1024 units, 4 hidden layers (128, 50, 10, and 10 units), and an output layer with 2 units were used to classify artifactual signal segments.  Artifacts were indicated to have been ‘eliminated’, which indicates that they were removed outright.  **Validation:** Model was trained using the data of 6 patients, tested using the data of 24 patients  **Post-processing:** Removal/rejection of artifactual points | Expert labeled waveforms | Using a train/test validation split of 6 and 24 patients, respectively, to reach a DBN prediction rate of 95%.  DBN achieved a sensitivity of 96.3% and a specificity of 95.4% for classifying normal and artifactual pulses | Deep belief network has good sensitivity and specificity  Can function in real-time with a 10-second delay  Resulted in an improvement in false alarms in the detection of hypotension and tachycardia | Manual segmentation required to train model, this would need to be reconducted prior to generalized application |
|  | **Sex (% Male)** | N/A |  |  |  |  |  |  |  |
|  | **Age** | N/A | **Device type** | Arterial line |  |  |  |  |  |
|  | **Pathophysiological health** | Severe TBI | **Location** | Radial artery |  |  |  |  |  |
| Lee et al. (2020) [75] | **Subjects** | 309 | **Signal type(s) used** | IBP | Stacked convolutional autoencoder (SCAE) and a convolutional neural network (CNN)  Only 10 patients worth of data used for testing and training  Steps in this method involve:  1. Onset detection  2. Normalization of pulse  3. Segmentation of pulse  4. Generation of representative images using SCAE  5. The representative images were labeled as either ‘valid’ or ‘artifactual’ using a CNN.  **Validation:** Performance was evaluated using leave-one-out 10-fold cross validation  **Post-processing:** Removal/rejection of artifactual points | Gold-standard was compared to two experts’ classifications  Three conventional machine learning methods including:  1. Linear support vector machine (LSVM)  2. radial basis function kernel support vector machine (KSVM)[143], [144]  3. Original CNN | Data from 10 patients (730,077 pulses) were used as training and testing data  Performance was measured using sensitivity, specificity, and accuracy, as well as the net prediction rate. Interrater agreement was assessed using F-score and G-score. The ‘gold standard’ for evaluation was two trained personnel that independently labeled the pulses.  Proposed model had sensitivity of 97.3% in ABP artifact classification and lowest rate of misclassification compared to other methods | ABP (and ICP) pulses were able to be correctly identified as valid or artifactual with a high degree of accuracy  Research conducted also indicated that the signal’s prognostic capacities increased with artifacts identified | Retrospective analysis with small cohort  Limited to TBI population  Potentially high false positive classification rate for proposed method  Data on other methods efficacies in identifying artifacts was not clearly presented. |
|  | **Sex (% Male)** | 75% |  |  |  |  |  |  |  |
|  | **Age** | 35 (IQR 23-47 years) | **Device type** | Arterial line |  |  |  |  |  |
|  | **Pathophysiological health** | TBI | **Location** | Radial artery |  |  |  |  |  |
| Howard et al. [76] | **Subjects** | 107 (train)  53 (test) | **Signal type(s) used** | IBP (200 Hz)  ECG | The presented method is a two-step convolutional neural network (CNN) which has inputted beats that were segmented using a simultaneously recorded ECG signal:  1. 1-D CNN for single beat classification  2. Long-term recurrent CNN trained using outputs from Step 1 to improve decisions based on consecutive beats  There were 3,855 beats randomly selected across dataset for testing and 1,854 beats for training. These beats were classified as ‘normal’, ‘noisy non-dampened’, ‘port open’ (of catheter), and ‘damped’ by algorithm. The ‘dampoid’ category was added for reviewers.  **Validation:** Trained and tested using a 3:1 ratio based on internal lab classifications, then externally validated using labels from an external lab  **Post-processing:** None, only designed for artifact identification and classification | Training set was reviewed by internal core lab and test set was reviewed by both internal and external core lab. | The proposed neural network was 99.4% accurate (95% CI: 98.8% to 99.6%) for 1,854 testing beats that were normal, depicting dampening, or artifactual.  For ‘damping’: sensitivity = 100%  For ‘dampoid’: sensitivity = 98.5%  For ‘normal’ or ‘noisy non-dampened’: sensitivity = 99.8%  Cohen’s kappa = 0.970  F1 score = 0.976  PPV for ‘damping’ = 98.1%  NPV for ‘damping’ = 99.5%  For external physician that did not influence training:  Inter-rater agreement was 98.7% (95% CI: 98.0% to 99.2%). Cohen’s kappa was 0.943 and F1 score of 0.963.  Network agreed more with core laboratories respectively (99.4% and 98.7%) compared to them between each other (98.2% of beats) (however, it was not statistically significant) | The neural network performed well at classifying artifacts.  Performed better compared to inter-lab raters than between raters.  Developed using individuals with various cardiovascular health  Time taken to process a beat was under 1 second | Strong performance but more work can be done to improve sensitivity |
|  | **Sex (% Male)** | 70.1% (train)  77.4% (test) |  |  |  |  |  |  |  |
|  | **Age** | 60.3 ± 9.7 (train)  61.3 ± 10.3 (test) | **Device type** | Catheter |  |  |  |  |  |
|  | **Pathophysiological health** | Invasive coronary angiography | **Location** | Aortic artery |  |  |  |  |  |
| Son et al. [77] | **Subjects** | 30 (6 for training, 24 for testing) | **Signal type(s) used** | IBP (100 Hz, 15 second intervals; 20 per patient), but cuff inflation also present | The proposed algorithm is a deep belief network (DBN) designed for the automatic removal of ABP artifacts. The ABP data was first converted into pulse-wise probabilistic input data which was the input to the visible layer. The steps for this included:  1. Segmenting the pulses based on the location of troughs relative to the systolic peak of the pulses  2. Labeling pulses by two experts  3. Interpolating the pulses using cubic spline interpolation such that all pulses were of the same size  4. Normalization of pulse size between 0 and 1  The DBN had 5 hidden layers with 128, 50, 10, 10, and 2 nodes, respectively. It had a learning rate of 0.1, momentum of 0.9 and a weight decay of 0.001.  The training data set was 465,635 and the number of training iterations was evaluated using the test dataset of 2,317,153. Performance had diminishing returns after 1,000 iterations using 20% of the dataset with a net prediction rate of 95.88%. Additionally, 1% and 10% of the data was also evaluated.  **Validation:** Trained using data from 6 patients, tested on data from 24 patients  **Post-processing:** Indicated to remove artifactual points, but seems as though they are filled in using linear interpolation | Gold-standard was compared to two experts who conducted independent classification  Waveform morphology dependant method proposed by Sun et al. [25] referred to as the signal abnormality index (SAI)  An extension of the Sun et al. method presented by Zhang et al. that incorporates slow ejection slope sum (SAI+ SESS) and end-diastole slope sum (SAI + EDSS) [88] | Training data was 465,635 pulse data instances and testing data was 2,317,153 instances. Training data sizes of 1% (DBN1), 10% (DBN10), and 20% (DBN20) were evaluated (which were randomly selected).  The net prediction of the optimal DBN classifier was higher than the SAI, SAI + SESS, and SAI + EDSS methods  DBN1 had highest positive likelihood ratio (LR+) and specificity with values of 54.94% and 98.33%, respectively. However, the DBN20 method had the best comprehensive score, net prediction, and sensitivity. It was overall the better result. DBN20 had results of:  Sens = 96.34%  Spec = 95.42%  LR+ = 21.05%  Negative likelihood ratio (LR-) = 0.04%  Acc = 95.49%  NPV = 95.88%  A summary receiver operating characteristic (SROC) curve was used to evaluate classification on a subject specific basis. SAI achieved a value of 0.918 and DBN20 achieved a value of 0.969. DBN20 also outperformed SAI in detection of specific artifact types (motion, biological, cuff inflation, and transducer flushing), with the exception of sensitivity in transducer flushing artifacts. | DBN20 had the best performance overall the testing dataset and outperformed all other methods in terms of balancing sensitivity and specificity amongst all subjects.  DBN training time was twice as long as SAI, SAI + EDSS, and SAI + SESS; however, it remained below a level that indicated any effect on real-time processing.  Real-time test ratio was below 0.2  Lower false positive rate than is typical  Can potentially be extended to other vital signals | DBN training time was longer than SAI  Small cohort, only those with TBI  Insufficient instances of patient movement  Further work needed to elucidate error cost of mis-classification |
|  | **Sex (% Male)** | N/A | **Device type** | Catheter |  |  |  |  |  |
|  | **Age** | N/A | **Location** | Radial artery |  |  |  |  |  |
| Pike and Mustard [78] | **Subjects** | 38 | **Signal type(s) used** | IBP (100 Hz, 15 second intervals; 20 per patient) | Manually labeled by authors, peaks were segmented, manually labeled as ‘over dampened’, ‘clean’ or ‘dirty’, authors had access to clinical information about patient. Two groups were formed of 19 patients. Two networks were trained and tested, each using one set for train and the other test using back propagation. Architecture had 70 input nodes, 20 hidden, and 3 element output layer (to label as one of the three categories) where the inputs are peaks (algorithm optimization explained in more detail in article) and a tuning parameter is used, and algorithm is optimised by passing through the datasets and then analyzing the ABP waveform pulses separately. The input neurons represent points in the pulse as well as data regarding the pulses surrounding it (shape and variability).  **Validation:** Trained and tested, exact split was not clearly disclosed  **Post-processing:** Removal/rejection of artifactual points | Manual annotation of pulses with patient information available | For Network 1:  Group 1(training): FP = 0.008816, FN = 0.031370  Group 2 (testing): FP = 0.012307, FN = 0.198583  For Network 2:  Group 1(training): FP = 0.032258, FN = 0.054401  Group 2(testing): FP = 0.004662; FN = 0.041488 | Running the learning procedure for longer reduced the false positive error rate but heightened the false negative rate  Stable representation for wide variety of peak shapes. Preserves information about width and height of peaks. Algorithm is stable. Easy to implement. | Lots of input values  Can lose information during rescaling |
|  | **Sex (% Male)** | N/A |  |  |  |  |  |  |  |
|  | **Age** | N/A | **Device type** | Catheter |  |  |  |  |  |
|  | **Pathophysiological health** | Various in ICU | **Location** | Radial artery |  |  |  |  |  |
| Cabeleira et al. [79] | **Subjects** | 58 from CENTER-TBI [145] | **Signal type(s) used** | ABP (>100 Hz), presumed to be IBP based on application | Based on SAX algorithm [146], which uses a pulse in the time domain into a SAX string (which is a set of letters). ‘Words’ are classified using a support vector machine (SVM) with no specified kernel. The SAX values were segmented using a method presented by Scholkmann et al. [147], they were then normalized and rescaled with each segment being represented by 6 letters. Applied to data of 50 patients to create a dictionary of SAX values and their corresponding mean pulses. The 700 most common SAX strings (which composed 99.75% of the training pulses) were manually classified. The training dataset resulted in a dictionary of SAX strings and their corresponding mean pulses with 3,330 entries.  The testing dataset composed of a balanced number of labeled valid and artifactual ABP pulses was selected from 8 patients.  **Validation:** Trained using pulses from 50 patients to develop the library, tested on data from 8 patients  **Post-processing:** Removal/rejection of artifactual points | Two independent manual classifiers | Compared to the independent classifiers:  Sensitivity = 97.2% and 95.4%, respectively  Specificity = 87.7% in both | High sensitivity, specificity could be higher.  Able to function on its own without any other inputs than ABP  Good at eliminating particular types of artifacts seen during bedside monitoring  Feasible for real-time use | Resolution of SAX strings resulted in a higher false negative rate  Cannot identify when signal appears normal but is outside of the normal range  Subject to bias from those constructing the algorithm  Article indicated needing to improve pulse cutting algorithm used  No indication of number of patients used to test, just specified that it was commonly seen artifacts. |
|  | **Sex (% Male)** | N/A |  |  |  |  |  |  |  |
|  | **Age** | N/A | **Device type** | N/A |  |  |  |  |  |
|  | **Pathophysiological health** | TBI | **Location** | N/A |  |  |  |  |  |
| Edinburgh et al. [80] | **Subjects** | 1 | **Signal type(s) used** | IBP (125 Hz) | Proposed method is referred to as “DeepClean” which is a Variational autoencoder (VAE)-based pipeline for classifying ABP waveforms. This model is a version of generative modelling which learns distribution to approximate a ‘true’ dataset. A subset of this is latent variable models which use unobserved variables to represent hidden structures in data and employ a deterministic function to map these variables into the variable space using a decoder network. The aim of the algorithm is to compute the marginal likelihood by integrating over all values of the unobserved variable. A methodology of doing this is using VAEs which resolve the integral using variational Bayesian inference. The DeepClean algorithm uses VAEs for the detection of ABP artifacts and imputes missing regions. VAEs were constructed using CNNs Trained, tested, and validated on 10 seconds segments of clean or artifactual data. Training data was cleaned of gross artifacts. A balanced test set was constructed to encompass different morphological circumstances.  **Validation:** Training and validation had a ratio of ~9:1  **Post-processing:** Removal/rejection of artifactual points | Manually annotated by two of the authors as artifactual or normal. This was done on the 10 second level as well as on a waveform level.  PCA was used as a comparison method for artifact identification | Measured using the MSE between the reconstructed signal and the sample  The number of latent dimensions used for each of these methodologies varied.  For mean latent dimensions, the metrics for VAE:  Acc = 0.901  Sens = 0.919  Spec = 0.868  AUCROC = 0.973  For PCA:  Acc = 0.613  Sens = 0.460  Spec = 0.896  AUCROC = 0.487 | Strong performance of VAE-based deep learning algorithm to correctly label valid or artifactual waveforms | Was based on single patient data |
|  | **Sex (% Male)** | N/A | **Device type** | Arterial line to pressure transducer (Baxter Healthcare Corp. CardioVascular Group, Irvine, CA) |  |  |  |  |  |
|  | **Age** | Adult | **Location** | Radial artery |  |  |  |  |  |
| Ouyoung et al. [81] | **Subjects** | 60 | **Signal type(s) used** | NIBP (1024 Hz) | Signal processing involves the frequency domain analysis. There are 4 indices (amplitude proportion, coefficient of variation, phase angle, and standard deviation of phase angle) for 10 harmonic indices. There are 40 blood pressure waveforms used as inputs.  Information processing for the binary classification of the data involved 8 different machine learning methods. These include the SVM, multilayer perceptron (MLP), Gaussian Naïve Bayes (GNB), decision tree (DT), random forest (RF), logistic regression (LR), linear discriminant analysis (LDA), and k-nearest neighbor (KNN). Classification involved labeling data as high-quality and low-quality pulses.  **Validation:** Three validation techniques were used: three-fold cross-validation and testing using a 2:1 split, leave-one-subject-out testing, and a hold-out test with an 8:2 split between three-fold cross-validation and testing  **Post-processing:** None, algorithm was designed only to identify and classify pulses | Manual annotation was conducted by two observers | Three-fold cross validation was conducted using 67% of the data for training and 33% for testing. All eight methods had similar AUCROC values of approximately 0.8 (but spanned from 0.74 to 0.85). The highest values belonged to the MLP and RF algorithms with AUCs of 0.83 and 0.85, respectively. For MLP, mean acc = 83.54%, sens = 0.84, and spec = 0.88. For RF, mean acc = 85.36%, sens = 0.88, and spec = 0.85.  Leave-one-subject-out was conducted indicated that all models had AUCROC of approximately 0.90 (ranging from 0.78 to 0.96). It was largest for RF and KNN with AUCROC values of 0.96 and 0.93, respectively. For RF, acc = 88.33%, sens = 0.80, and spec = 0.9667.  Hold-out test was conducted where three-fold cross validation was conducted during evaluation and training/validation was split into 80/20. For RF, mean acc = 84.25%, sens = 0.74, spec = 0.92, and AUCROC = 0.84 for threefold cross validation and were acc = 80.56%, sens = 0.99, spec = 0.68, and AUCROC = 0.84. For KNN, acc = 84.78%, sens = 0.92, spec = 0.80, AUCROC = 0.86 | Performs similarly to methods outlined for PPG signals  RF algorithm had best performance  Contact pressure affects the pulse-wave measurements | Small cohort  All healthy subjects |
|  | **Sex (% Male)** | 55% |  |  |  |  |  |  |  |
|  | **Age** | 23.6 ± 0.8 | **Device type** | Pressure transducer (KFG-2-120-D1-11, Kyowa, Hong Kong, China) held to skin |  |  |  |  |  |
|  | **Pathophysiological health** | Healthy, different levels of contact pressure was applied | **Location** | Radial artery |  |  |  |  |  |

*ABP = Arterial Blood Pressure, Acc = Accuracy, , CNN = Convolutional Neural Network, DBN = Deep Belief Network, DBP = Diastolic Blood Pressure, DT = Decision Tree, ECG = Electrocardiogram, F1 = F1 Score, GNB = Gaussian Naïve Bayes, HR = Heart Rate, IBP = Invasive Blood Pressure, ICU = Intensive Care Unit, KNN = K-Nearest Neighbour, KSVM = Kernel Support Vector Machine, LDA = Linear Discriminant Analysis, LR = Logistic Regression, LR+ = Positive Likelihood Ratio, LR- = Negative Likelihood Ratio, LSTM = Long Short-Term Memory, MAP = Mean Arterial Pressure, MLP = Multilayer Perceptron, MSE = Mean Squared Error, NIBP = Non-Invasive Blood Pressure, NP = Net Predictive Value, NPV = Negative Predictive Value, Prec = Precision, PPV = Positive Predictive Value, Rec = Recall, SAI = Signal Abnormality Index, SBP = Systolic Blood Pressure, SCAE = Stacked Convolutional Autoencoder, Sens = Sensitivity, SROC = Summary Receiver Operating Characteristic, Spec = Specificity, SVM = Support Vector Machine, TBI = Traumatic Brain Injury, VAE = Variational Autoencoder*

**Table D.2. High-frequency ABP artifact detection – wavelet transformation-based methods**

| **Reference** | **Subject information** | | **Data type (sampling rate) – system type** | | **Artifact removal method(s) used** | **Methods compared** | **Effectiveness** | **Study results and conclusions** | **Limitations** |
| --- | --- | --- | --- | --- | --- | --- | --- | --- | --- |
| Nguyen et al. [82] | **Subjects** | 104 | **Signal type(s) used** | NIBP (100 Hz) | Band pass filter based on empirical wavelet transform designed to detect first harmonic of ‘carrier’ and two side bands of oscillometric wave  **Validation:** Model was developed without training and was directly applied to a testing dataset  **Post-processing:** Removal/rejection of artifactual points/noise | Measurements of nurses for systolic and diastolic blood pressure which were compared to the algorithmic estimation of these parameters using maximum amplitude (MA) and maximum slope (MS) | Four subjects, there was significant improvement in the accurate detection of DBP and SBP after filtering the signal, within 5 mmHg error: without filtering was 0% in SBP and 50% in DBP, with filtering was 75% in SBP and 100% in DBP across 4 patients with arm motion.  There were also improvements seen when the estimation of blood pressure using the proposed device and algorithm was generalized to 100 patients when compared to nurse readings | Filtering using the proposed algorithms made it such that there were fewer errors when using an algorithm to detect SBP and DBP when measuring using an oscillometric BP prototype | Potential bias with proposed system  Old method for BPF, with newer better methods developed |
|  | **Sex (% Male)** | N/A |  |  |  |  |  |  |  |
|  | **Age** | N/A | **Device type** | Oscillometric BP prototype |  |  |  |  |  |
|  | **Pathophysiological health** | Healthy, instructed to relax and then move | **Location** | Upper arm |  |  |  |  |  |
| Xu et al. (2007) [84] | **Subjects** | Not specified, 500 pulses selected of 5395 clinically measured pulses in segments of 1 min to 10 min | **Signal type(s) used** | TCPD (100 Hz) | This article is an refinement of the method presented in Xu et al. (2005) [83].  1. Signal is decomposed using the Meyer wavelet, in this method into seven levels.  2. The ER is calculated this time using the first level and seventh level decompositions of the signal. The wavelet filter can inadvertently add some distortion to the signal if the baseline wander is not significantly large. If the ER exceeds a certain level, it bypasses the first filter directly to the second filter.  3. The stage 1 filter is a Meyer wavelet filter which removes levels of the signal that correspond to the baseline wander  4. The stage 2 filter is the cubic spline filter that removes any remaining baseline wander not removed using the stage 1 filter. The onsets of pulses are detected; these are then used as “knots” for the cubic spline interpolation to estimate the baseline wander.  **Validation:** Model was developed without training and was directly applied to a testing dataset  **Post-processing:** None, only designed for baseline noise removal | Baseline was three human observers who manually annotated 500 pulses  The CAF method was compared to the traditional linear-phase least-squares error FIR filter (FIRLS), morphological filter, and the cubic spline method in isolation | Several methods of evaluation and comparison were used:  1. Filtered experimental signals were exposed to added baseline wander which had variance in amount. The PDR and BCR results indicated that the CAF method outperformed the other methods with added periodic, non-periodic, and combined baseline wander. However, the cubic spline method was able to function well in instances of ER being over 50 dB.  2. CAF was tested on 5 common pulse types including ‘taut’, ‘smooth’, ‘normal’, ‘slow’, and ‘rapid’. It showed the lowest PDR values for the normal and taut pulses (below 0.003 for range of ER from 10 to -20 dB) and highest values for slow pulses (below 0.014 for range of ER from 10 to -20 dB), which indicates more distortion  3. Experimental results were calculated for 500 recorded pulses. Human identification rose from 67% to 83% after being filtered using the CAF method | The CAF filter has optimum performance when the ER is below 50 dB  The developed method was functional for the TCPD signal but was also able to be used with ECG signals  Developed method maintains the underlying signal well | ER threshold must be manually selected  Does not appear to address any high frequency noise or wander  Less functional on slow waves  Insufficient cohort size or descriptions regarding their health |
|  | **Sex (% Male)** | N/A |  |  |  |  |  |  |  |
|  | **Age** | N/A | **Device type** | Custom pulse acquisition device comprised of pulse sensor, amplifier, and computer |  |  |  |  |  |
|  | **Pathophysiological health** | N/A | **Location** | Radial artery |  |  |  |  |  |
| Xu et al. (2005) [83] | **Subjects** | Not specified, 500 of 5395 clinically measured pulses used | **Signal type(s) used** | Traditional Chinese pulse diagnosis (TCPD) although plots provided indicate pulse features consistent with blood pressure | This algorithm was designed to remove the baseline drift from the TCPD signals. This methodology involves the following steps:  1. The signal is decomposed into 6 levels using the Meyer wavelet. The energy ratio (ER) is then used to determine if the baseline drift is great enough to be removed using the wavelet-based method. The energy ratio is calculated using the following equation:  $ER=20{log}_{10}\frac{\left\Vert A1-mean(A1) \right\Vert}{\left\Vert A6-mean(A6) \right\Vert}$  Where A1 and A6 are the first and sixth level decompositions, respectively, where A1 is intended to be the pulse signal and A6 is the baseline drift. An ER threshold is used to determine whether the baseline drift is great enough to be processed using wavelet filtering.  2. For signals with an ER of greater than 50 dB, a discrete Meyer wavelet filter was applied; however, the structure of the filter was not clear. If the ER is less than 50 dB (which was 8% of the clinically recorded pulses), the algorithm immediately moves on to the next step.  3. Cubic spline estimation is then applied, the ‘knots’ of the estimation are assumed to be the pulse onsets, and this cubic spline estimation is indicated to be filtering the pulses.  **Validation:** Model was developed without training and was directly applied to a testing dataset  **Post-processing:** None, only designed for baseline noise removal | Three human experts performing diagnostics with and without the filtering | Most validation was conducted on pulses with simulated noise, which was out of the scope of this review.  CAF filtering was indicated to improve the diagnostic accuracy of these pulses from 67% to 83%. | It is indicated that the CAF method provides the best results for maintaining diagnostic information while removing baseline drift compared to other methods compared. | Small, poorly outlined sample size with methodological information poorly outlined.  Filters not well described. |
|  | **Sex (% Male)** | N/A |  |  |  |  |  |  |  |
|  | **Age** | N/A | **Device type** | N/A |  |  |  |  |  |
|  | **Pathophysiological health** | N/A | **Location** | N/A |  |  |  |  |  |

*ABP = Arterial Blood Pressure, BCR = Baseline Correction Ratio, BP = Blood Pressure, BPF = Band-Pass Filter, CAF = Cascaded Adaptive Filter, DBP = Diastolic Blood Pressure, ER = Energy Ratio, FIR = Finite Impulse Response, FIRLS = Linear-Phase Least Squares Error Finite Impulse Response Filter, MA = Maximum Amplitude, MS = Maximum Slope, NIBP = Non-Invasive Blood Pressure, PDR = Pulse Distortion Ratio, SBP = Systolic Blood Pressure, SNR = Signal-to-Noise Ratio, TCPD = Traditional Chinese Pulse Diagnosis*

**Table D.3. High-frequency ABP artifact detection – motion-based methods**

| **Reference** | **Subject information** | | **Data type (sampling rate) – system type** | | **Artifact removal method(s) used** | **Methods compared** | **Effectiveness** | **Study results and conclusions** | **Limitations** |
| --- | --- | --- | --- | --- | --- | --- | --- | --- | --- |
| Abderahman et al. [85] | **Subjects** | 5 | **Signal type(s) used** | Oscillometric NIBP with systolic, diastolic pressures estimated using MAP, and 3-axis accelerometer, ECG (2,000 Hz), ECG is used to help identify individual peaks | Two algorithms proposed:  1. Intrinsic mode functions selective analyzer (IMFSA) – oscillometric waveforms and the accelerometer signals are broken down into intrinsic mode functions (IMFs) which were differentiated between as motion artifacts, noise, and signal. Values removed from artifact regions are compensated by magnifying signal-related IMFs. The outputs of the functions that remove the noise and magnify the IMFs of the signal are merged using a combination of minimum and averaging operations to reconstruct a clean signal.  2. Intrinsic mode functions collector (IMFC) – detected vibrations in the signal are processed using EMD, the signal is broken down into IMFs and removes those that correspond to vibrational noise.  **Validation:** Model was developed without training and was directly applied to a testing dataset  **Post-processing:** None, only designed for artifact suppression | Reference device Om- ron HEM-790IT monitor (conventional oscillometric recording method) | The estimations of systolic and diastolic blood pressures pre- and post-artifact suppression. Metrics were mean error (ME) [mmHg], MAE [mmHg], and standard deviation of error (SDE) [mmHg].  Using IMFC for vibration artifacts  ME in SBP: 1.7 to -0.6  ME in DBP: 1.9 to -0.4  MAE in SBP: 3.1 to 0.8  MAE in DBP: 3.5 to 1.1  SDE in SBP: 3.8 to 1.0  SDE in DBP: 5.5 to 1.5  Using IMFSA for transient motion artifacts  ME in SBP: -0.9 to 0.3  ME in DBP: 3.2 to 0.0  MAE in SBP: 9.4 to 2.4  MAE in DBP: 5.3 to 2.4  SDE in SBP: 11.6 to 3.1  SDE in DBP: 8.7 to 2.9 | The IMFSA algorithm performed well compared to a median filter proposed in a previous work [148]  Reduced the impacts of vibrations and motion artifacts of recorded oscillometric signal | Motion artifacts were simulated  There was a small sample size that were all of the same demographic  Validated against a measured signal, no mention of inspection  Long processing time |
|  | **Sex (% Male)** | 100% |  |  |  |  |  |  |  |
|  | **Age** | 22 to 30 years | **Device type** | Accelerometer embedded in BP cuff |  |  |  |  |  |
|  | **Pathophysiological health** | Healthy | **Location** | Arm |  |  |  |  |  |

*BP = Blood Pressure, DBP = Diastolic Blood Pressure, ECG = Electrocardiogram, EMD = Empirical Mode Decomposition, IMF = Intrinsic Mode Function, IMFC = Intrinsic Mode Functions Collector, IMFSA = Intrinsic Mode Functions Selective Analyzer, MAE = Mean Absolute Error, MAP = Mean Arterial Pressure, ME = Mean Error, NIBP = Non-Invasive Blood Pressure, SBP = Systolic Blood Pressure, SDE = Standard Deviation of Error*

**Table D.4. High-frequency ABP artifact detection – signal quality-based methods**

| **Reference** | **Subject information** | | **Data type (sampling rate) – system type** | | **Artifact removal method(s) used** | **Methods compared** | **Effectiveness** | **Study results and conclusions** | **Limitations** |
| --- | --- | --- | --- | --- | --- | --- | --- | --- | --- |
| Sun et al. [25] | **Subjects** | 120 from MIMIC-II [99] | **Signal type(s) used** | ABP (125 Hz) | The proposed algorithm assesses pulses within the ABP signal using a signal abnormality index (SAI). This algorithm involves the following steps:  1. Beat detection using the algorithm developed by Zong et al. (2003) [15]  2. The detected onsets of the beats were used as a basis to extract features from the pulses; features included: HR, SBP, DBP, PP, MAP, beat period, and ‘noise’ which depicted the mean of negative slopes.  3. The features extracted were evaluated based on ‘abnormality criteria’ which were developed to analyze the pulses compared to physiological values, amount of noise, and amount of beat-to-beat variance (sudden changes present in beat that is absent from neighboring beats) for each pulse  4. The beats were classified using binary classification where all abnormality criteria were consolidated. Classification functioned using the logical OR operator.  5. A developed cumulative SAI (cSAI) was used to determine the fraction of flagged (artifactual) beats there were within a segment.  **Validation:** Model was developed without training and was directly applied to a testing dataset  **Post-processing:** None, only designed for artifact identification | Human expert, asked to classify under 4 categories of irregularity (none, minor, substantial irregularity present in one location, and major irregularity), likely had access to the simultaneously recorded CBFV signal  Minor and substantial irregularity categories are referred to as ‘gray zones’ | There were 256 segments of ABP data each with a length of 10 seconds that were classified using binary classification. Including gray zones the results were:  PPV = 0.63  NPV = 0.97  Sens = 0.90  Spec = 0.86  Excluding the gray zones:  PPV = 0.73  NPV = 1  Sens = 1  Spec = 0.91 | Sensitivity analysis indicated that each criterion only flags a portion of the beats. Shows the changes to abnormality criteria changing the cSAI. Sensitivity was defined when the normalized threshold for each was 1.  Algorithm is able to improve cardiac output estimation | No established gold standard against which to compare  Abnormality is application dependant |
|  | **Sex (% Male)** | N/A |  |  |  |  |  |  |  |
|  | **Age** | N/A | **Device type** | N/A |  |  |  |  |  |
|  | **Pathophysiological health** | ICU | **Location** | N/A |  |  |  |  |  |
| Zong et al. (2004) [86] | **Subjects** | 20 train and 26 test from MIMIC-I [113] | **Signal type(s) used** | IBP (125 Hz), multi-lead ECG (500 Hz) | Algorithm was based on a beat-to-beat signal quality analysis coupled with the relationship between ECG and ABP signals. Steps involved:  1. ABP signal assessed on beat-to-beat basis to calculate signal quality index (SQI). This leveraged a pulse detection algorithm, waveform feature extraction, and fuzzy representation and reasoning.  2. SQI value modified based on ECG and its relationship to the ABP signal  3. High SQI valued (above 0.5) signal segments used were used to improve short-term averaged blood pressure values  4. Acceptance and rejection of alarms were based on short term averaged BP values and SQIs of recent pulses  **Validation:** Training using 445 ABP alarms, tested using 604 ABP alarms  **Post-processing:** None, only designed for artifact identification | Alarms events classified by authors as valid or artifactual, some rejected if signal was oversaturated | For the development data, there were 445 ABP alarms, 126 (28.3%) of which were false based on inspection. The algorithm rejected 117 of 126 false alarms (92.9%) and two rejected true alarms. The false alarm rate was reduced from 28.3% to 2.8% while rejecting 0.4% of true alarms.  For test dataset, there were 604 ABP alarms, 164 (26.8%) of which were false alarms. The algorithm rejected 159 (98.2%), reducing the false alarm rate from 28.6% to 0.4%, and only rejected 0.2% of the true alarms. | Performed with a high degree of sensitivity and specificity  Functions well on testing dataset | Requires input of ECG  Future improvements to parameters could lead to no false rejections |
|  | **Sex (% Male)** | 50% (train), 62% (test) |  |  |  |  |  |  |  |
|  | **Age** | 52 to 92 years (train), 21 to 92 years (test) | **Device type** | Catheter |  |  |  |  |  |
|  | **Pathophysiological health** | ICU, various conditions | **Location** | Radial artery |  |  |  |  |  |
| Abdul Sukor et al. (2012) [87] | **Subjects** | 25 (4 recordings each) | **Signal type(s) used** | NIBP (1,000 Hz), Auscultatory BP (AuBP)  ECG | The proposed algorithm was designed to classify noise within the NIBP signal and estimate SBP and DBP. This involves:  1. Pre-processing – this involved cuff-pressure being down sampled to 100 Hz, applying sixth order Butterworth filter between 0.5 and 5 Hz (based on maximum heart rate). Signal was then segmented using a maximum filter and estimating the minimum within the peak-to-peak interval.  2. Noise classification – this was first conducted using morphology. Interruptions in the gradual decrease indicated a disruption in cuff airflow and an acoustic artifact in the NIBP signal using difference between cuff pressure and filtered cuff pressure. Second method conducted based on Korotkoff pulse width  3. Systolic and diastolic estimation feasibility – systolic point is identified as the time of the first two Korotkoff sounds within a time span less than twice the mean beat-to-beat interval. Diastolic point is identified as either (1) the time since the last Korotkoff pulse exceeds twice the mean beat-to-beat interval or (2) the diastolic point is identified as the trough in the oscillometric signal immediately followed by a Korotkoff pulse. These are only considered valid if a detected noise section is further than two mean beat-to-beat intervals from the event.  **Validation:** Model was developed without training and was directly applied to a testing dataset  **Post-processing:** Rejection/removal of artifactual points | Human operator listening to Korotkoff sounds, recorded systolic, diastolic, and noisy events (reference standard (RS)). Two individuals manually annotated the cuff pressure, AuBP, and ECG signal. The AuBP and ECG had been filtered. Scored individually and together (Raters 1 and 2) | Some errors were intentionally introduced into the signal through instructed patient motion. Accuracy, sensitivity, and specificity between Raters and between each Rater and the RS was never lower than 98.49%, 83.44%, and 98.72%, respectively.  For 100 pooled signals, the stepwise accuracy, sensitivity, and specificity were 97.00%, 80.61%, and 98.16%, respectively. Across all signals the averages were 97.06% ± 4.04%, 75.92% ± 28.63% (only able to be calculated for 65 signals where noise occurs), and 98.18% ± 3.83%, respectively.  Algorithm correctly determined whether systolic and diastolic pressure could be calculated in 93% of signals.  Algorithm developed by Park et al. for noise removal had better results in all signals for errors in systolic detection but substantially worse in diastolic detection. | Noisy sections in the Korotkoff sounds were able to be accurately identified; however, the sensitivity was low.  Mean error of identification of temporal locations of systolic and diastolic pressures.  Demonstrated superior performance to denoising algorithm developed by Park et al. [149] in all signals. | Low sensitivity of noise detection.  Study conducted in lab environment  Artifacts were generated by performing specified movements. |
|  | **Sex (% Male)** | 64% |  |  |  |  |  |  |  |
|  | **Age** | 28 ± 5 years | **Device type** | Stethoscope 927–0017, TeleMedCare Pty. Ltd (AuBP), Cuff, MLT1100/DADInstruments, Sydney, Australia (NIBP) |  |  |  |  |  |
|  | **Pathophysiological health** | Healthy | **Location** | Brachial artery (AuBP) and arm (NIBP) on each arm |  |  |  |  |  |
| Zhang et al. [88] | **Subjects** | 75 (78 recordings after 4 omitted) | **Signal type(s) used** | NIBP (100 Hz), CBFV (using TCD) | Artifacts were identified as square wave, signal before and after wave, movement artifacts, and missing signal. Two features were proposed to address the difficulty that the Sun et al. method [25] faced with square wave artifacts with normal signal morphology. These two features were end diastole slope sum (EDSS) and slow ejection slope sum (SESS) which indicate the total change at the end of diastole and the total change during slow ejection, respectively. These features were compared to the maximum positive BP slope (MPPS) and maximum duration up-slope duration (MUSD) metrics proposed by Zong et al. [86]. All of the recordings were split into normal and abnormal segments by human observers, the cumulative probability of each feature across both sets was used to determine how well they were able to differentiate between the two. ROC analysis was used to optimize each of the four features and their corresponding parameters. The proposed algorithm sequentially completed the following steps:  1. Low-pass filtering  2. Identification of pulses using the method developed by Zong et al. [15]  3. Extraction of features from pulses  4. Evaluation of pulses using SAI criteria as well as one of the four aforementioned features  5. Criteria consolidated pulses labeled as either normal or abnormal.  **Validation:** Model was developed without training and was directly applied to a testing dataset  **Post-processing:** None, only designed for artifact identification/classification | Human expert annotations likely with access to the simultaneously recorded CBFV signal, served as reference point, the algorithm was compared to the method developed by Zong et al. using fuzzy logic [86], the SAI-based method [25] | The performance of each of the methods with their respective optimized parameters for 128,342 beats were:  Method developed by Zong et al. [86]: Sensibility = 97.45%  Spec = 82.77%  For SAI:  Sensibility = 97.75%  Spec = 18.38%  For SAI + MUSD:  Sensibility = 79.43% Spec = 65.17%  For SAI + MPPS:  Sensibility = 93.11% Spec = 82.48%  For SAI + SESS:  Sensibility = 94.99% Spec = 60.20%  For SAI + EDSS:  Sensibility = 93.95% Spec = 84.87% | SAI+EDSS had strong performance in both sensibility and specificity.  The Zong et al method outperformed the proposed in sensibility; however, the learning time is more substantial for this model | Features developed were based off of particular dataset |
|  | **Sex (% Male)** | 69.3% |  |  |  |  |  |  |  |
|  | **Age** | 53.28 ± 17.54 years | **Device type** | Colin tonometer |  |  |  |  |  |
|  | **Pathophysiological health** | N/A | **Location** | N/A |  |  |  |  |  |
| Ali et al. [89] | **Subjects** | Annotations from Zong et al. [86] (exact number not specified) | **Signal type(s) used** | IBP (125 Hz), ECG (500 Hz) | ABP and ECG signals were plotted into morphograms. Their relationship during the course of a heartbeat had a particular pattern. Deviations from the pattern (in one signal) and the corresponding perturbation in the morphogram and cross-correlation were used to signify an artifact. Thresholds were established to detect errors in ABP and ECG, as well as signal saturation, and overdamping of ABP.  **Validation:** Model was developed without training and was directly applied to a testing dataset  **Post-processing:** None, only designed for artifact identification | Manual annotation, likely had access to both the ABP and ECG signals | 160 manually identified artifacts, 144 correctly detected by algorithm, 16 missed  523 manually annotated true alarms, 518 of which were correctly detected, 5 mistaken as artifacts.  Total sensitivity of 90% and specificity of 99%. | Simple method to detect artifacts using perturbations in morphograms observed by clinicians and incorporated into an algorithm | Reliant on multiple ECG signals to detect artifacts  Limited information regarding the dataset on which the method was tested  Only developed for alarm errors |
|  | **Sex (% Male)** | N/A |  |  |  |  |  |  |  |
|  | **Age** | N/A | **Device type** | Catheter |  |  |  |  |  |
|  | **Pathophysiological health** | ICU, various conditions | **Location** | Radial artery |  |  |  |  |  |
| Ali and Eshelman [90] | **Subjects** | 681 alarms from MIMIC-I | **Signal type(s) used** | IBP (125 Hz) and ECG (500 Hz) | Morphograms are created by plotting ABP and ECG signals on same plot. Correlated signals in heartbeat are used to either detect clinically significant events (when all signals affected) or artifacts (when one signal is affected). Typical morphograms between ECG signals and ABP-ECG signals have a typical shape on morphograph and are labeled using thresholds.  **Validation:** Model was developed without training and was directly applied to a testing dataset  **Post-processing:** None, only designed for artifact identification | Manual annotation, provided by the public dataset | Tested 681 ABP alarms from the MIMIC-I database that had been manually annotated [150]. The proposed algorithm was able to correctly classify 98% of the true (clinically accurate ABP alarms) and 45% of the artifactual alarms | Lower artifact detection rate than the results proposed by Zong et al. [150].  Very simple method  Low misclassification of valid signals | Low success rate  Only used to detect artifactual ABP alarms |
|  | **Sex (% Male)** | N/A |  |  |  |  |  |  |  |
|  | **Age** | N/A | **Device type** | Catheter |  |  |  |  |  |
|  | **Pathophysiological health** | ICU, various conditions | **Location** | Radial artery |  |  |  |  |  |
| Nagai and Nagata [91] | **Subjects** | 8 rats + 1 rat with noise added | **Signal type(s) used** | IBP (1,000 Hz) | This algorithm is designed for waveform recognition and noise rejection. It initially uses secondary IIR Butterworth filters developed using frequency information from 12 rats to isolate SBP and DBP using cutoff frequencies of 8 Hz and 15 Hz, respectively. SBP is detected as the maximum value in the rising phase of the 8 Hz low-pass filtered BP signal. DBP is detected as the minimum value during the falling phase in the 15 Hz low-pass filtered BP signal. The phases are detected using first and second derivatives of the filtered BP signal. The absolute difference between sequential points in the DBP-DBP area are integrated to detect noise and other artifacts. Calculated waveform values (CWV) extracted from each pulse segmented using the digital filters include SBP, mean BP, DBP, HR, DBP-DBP time, DBP-SBP time, BP displacement, and DBP-DBP gradient values. The Smirnov rejection test is applied to the CWVs for rejection testing where average values for 20-beat segments are used to reject outliers. Waveform indices (WI) such as SBP, DBP, mean BP, and HR in a pulse are rejected if any of the CMV values are erroneous as are those in adjacent pulses are rejected. WI are smoothed using dynamically created secondary IIR Butterworth filters. The cutoff frequencies for these filters are based on intervals between specified points (i.e. if WI values are of interest at the points of drugs being administered). Time-delay compensation for the digital filter and a weighted function are also applied for smoothing.  **Validation:** Model was developed without training and was directly applied to a testing dataset  **Post-processing:** Rejection/removal of artifactual points | Without use of algorithm | There were 305,558 beats analyzed, 391 of 391 body movement artifacts were rejected, and 191 of 215 arrythmias were rejected (due to continuous bradycardia arrythmias) | High accuracy for removal of movement and arrythmias in data  Rejection of noise was successful in added noise in single rat | Data rejection  Small cohort  Bradycardial arrythmias not rejected |
|  | **Sex (% Male)** | N/A |  |  |  |  |  |  |  |
|  | **Age** | N/A | **Device type** | DX-360 Transducer |  |  |  |  |  |
|  | **Pathophysiological health** | Anaesthesia | **Location** | N/A |  |  |  |  |  |
| Charbonnier et al. [92] | **Subjects** | 2 | **Signal type(s) used** | NIBP (100 Hz) | Pulse information stored included maximum value, minimal value, and width (described using a threshold). 192 pulses (89 valid, 103 artifactual) from patient 1 used for training of discriminative function. Pulse information, as well as its pressure applied value and pulse sampled at 17 Hz (from 100 Hz), for 10 different reference pulses described as valid was stored. Various criteria including correlation coefficient, difference between maximum values, difference between minimum values, difference between width, and difference between pressure applied values were used with the discriminative function to determine validity of the pulse.  **Validation:** Training using data from patient 1, testing using data from patient 2  **Post-processing:** None, only designed for artifact identification | Cardiologist examined and assigned correctness  Artifact identification of commercial device | 674 pulses recorded on both patients, 432 artifacts.  In training on part of recording from patient 1, 100% in labeling valid signals, 66% in artifacts, for total of 82% correctly classified.  For validation on patient 1 (same one on which it was trained) (225 pulses, 78 valid and 177 artifactual), correct classification was 76%, sensibility of 86% and specificity was 71%.  For validation on patient 2, (222 pulses, 74 valid and 148 artifactual), correct classification was 74%, sensibility was 85%, and specificity is 68%.  Across three sets of data, specificity is 70%.  Commercial dataset correctly classified 41% of pulses, sensibility is lower than proposed by 13%, specificity is 25% | Vastly outperformed commercial method  Not very computationally intensive | Cardiologist was ‘very strict’  Small cohort |
|  | **Sex (% Male)** | 100% |  |  |  |  |  |  |  |
|  | **Age** | 23 and 34 | **Device type** | Save 33 BP recorder |  |  |  |  |  |
|  | **Pathophysiological health** | Presumed healthy  (1) Still  (2) Moving arm and fingers | **Location** | Upper arm |  |  |  |  |  |
| Lim et al. [93] | **Subjects** | 25 | **Signal type(s) used** | NIBP, ECG (1,000 Hz), Korotkoff sounds | The proposed algorithm involves the following:  1. Pre-processing – trend in the cuff pressure signal is removed using a first-order band-pass Butterworth which allows for the signal to be transformed into a pulsatile oscillometric waveform. A forward-backward filter is used to obtain zero-phase response. ECG signal (unaffected by movement) is then used to obtain cardiac cycle. The reference systolic BP ratio and diastolic BP ratio were determined as the ratio of systolic BP and diastolic BP amplitude to maximum amplitude.  2. Removal of artifacts – cubic spline was fitted to the oscillometric waveform envelope (OWE). Artifacts were identified automatically using several criteria including sudden increases in cuff pressure during deflation, oscillometric pulses relative to neighboring pulses, and different waveform morphological characteristics (ex. distances between peaks, peak to trough distance etc.).  3. Feature extraction – 10 features were extracted from OWE including area under curve, amplitude, and duration, among others.  4. Blood pressure estimation models – three different blood pressure estimation models were evaluated. These included the Maximum Amplitude Algorithm (MAA), multiple linear regression (MLR), and support vector regression (SVR)  The sequential forward floating selection (SFFS) approach was used to identify the combination of features that would result in the best performance for BP estimation.  **Validation:** Four-fold cross validation was applied during the evaluation of the blood pressure estimation methods.  **Post-processing:** Rejection/removal of artifactual points | Reference was analyzed by two clinical experts who estimated the locations of the SBP and DBP based on the Korotkoff sounds (of 100 sounds only 81 SBP and 84 DBP were used due to contamination with noise) | To evaluate the abilities of the proposed algorithm to remove SBP and DBP errors. There was a significant improvement in the difference between the reference standard and MAA identified SBP and DBP values based on the Bland-Altman plot. The differences in mm Hg are:  Mean ± SD SBP (before) = 4.5 ± 28.6  Mean ± SD DBP (before) = 0.0 ± 14.2  Mean ± SD SBP (after) = -1.6 ± 8.6  Mean ± SD DBP (after) = 0.3 ± 6.7  Results regarding blood pressure characteristics estimation are included in the article.  Using the SFFS approach, there were two features selected (1. area under the OWE before the MA divided by the area under the OWE, 2. Area under the OWE after the MA). The result was a significant reduction in SBP error: where using MLR: mean ± SD = −0.3 ± 5.8 mmHg and using SVR: mean ± SD = −0.6 ± 5.4 mmHg). However, there was little improvement shown in DBP error. | The results indicate an improvement in SBP and DBP errors with the addition of artifact removal  Using variable characteristic ratios derived using several features from the OWE resulted in an improvement in the blood pressure estimation accuracy | Data was obtained from healthy subjects in a lab setting performing prescribed motions  Small cohort |
|  | **Sex (% Male)** | 64% |  |  |  |  |  |  |  |
|  | **Age** | 28 ± 5 years | **Device type** | Custom cuff (NIBP and ECG), stethoscope (Korotkoff sounds) |  |  |  |  |  |
|  | **Pathophysiological health** | Healthy | **Location** | Arms |  |  |  |  |  |

*ABP = Arterial Blood Pressure, AuBP = Auscultatory Blood Pressure, BCR = Baseline Correction Ratio, BP = Blood Pressure, CBFV = Cerebral Blood Flow Velocity, cSAI = Cumulative Signal Abnormality Index, CWV = Calculated Waveform Value, DBP = Diastolic Blood Pressure, ECG = Electrocardiogram, EDSS = End Diastole Slope Sum, ER = Energy Ratio, FIR = Finite Impulse Response, FIRLS = Linear-Phase Least-Squares Error Finite Impulse Response, HR = Heart Rate, IBP = Invasive Blood Pressure, ICU = Intensive Care Unit, MA = Maximum Amplitude, MAA = Maximum Amplitude Algorithm, MAP = Mean Arterial Pressure, ME = Mean Error, MLR = Multiple Linear Regression, MPPS = Maximum Positive BP Slope, MUSD = Maximum Upslope Duration, NIBP = Non-Invasive Blood Pressure, NPV = Negative Predictive Value, OWE = Oscillometric Waveform Envelope, PDR = Pulse Distortion Ratio, PP = Pulse Pressure, PPV = Positive Predictive Value, ROC = Receiver Operating Characteristic, RS = Reference Standard, SAI = Signal Abnormality Index, SBP = Systolic Blood Pressure, SD = Standard Deviation, Sens = Sensitivity, SESS = Slow Ejection Slope Sum, SFFS = Sequential Forward Floating Selection, SNR = Signal-to-Noise Ratio, Spec = Specificity, SQI = Signal Quality Index, SVR = Support Vector Regression, TCPD = Traditional Chinese Pulse Diagnosis*

1. **Supplementary File 5**

**Table E.1. Valid pulse identification**

| **Reference** | **Subject information** | | **Data type (sampling rate) – system type** | | **Artifact removal method(s) used** | **Methods compared** | **Effectiveness** | **Study results and conclusions** | **Limitations** |
| --- | --- | --- | --- | --- | --- | --- | --- | --- | --- |
| Aboy et al. [44] | **Subjects** | 210 | **Signal type(s) used** | IBP (125 Hz), ICP, SpO_2_  (IBP presumed based on application) | The proposed algorithm was designed to detect beats in ICP, ABP, and SpO_2_ signals by identifying waveform features. The first stage involves pre-processing using three different bandpass filters. Filter #1 removes both the trend as well as high frequency noise. The output of this filter is used to estimate the heart rate. The pressure signal is partitioned, and the power spectral density (PSD) is computed. The harmonic PSD, which incorporates both fundamental and harmonic components of the PSD, is used to estimate heart rate. The estimated heart rate is then used to determine the cutoff frequency for Filter #2 and Filter #3. Filter #2 also removes high frequency components, it is a bandpass filter that will only allow frequencies to pass that are 2.5 times smaller than the heart rate. At this point, the signal only contains one cycle per heart contraction. Filter #3 removes any trend from the signal by removing any frequencies that are slower than 0.5 times the heart rate frequency and removes any frequency greater than 10 times the heart rate frequency. Peak detection is conducted using two rank filters. Peaks above the 60^th^ percentile are initially selected using a 10 second moving window. The second filter detects maxima in the first derivative that are above the 90^th^ percentile. Decision logic is used to determine the inter-beat intervals (IBI). These IBIs are used to determine the temporal distance between pulses and determines if any pulses have been missed or falsely detected when compared to the estimated heart rate. There are impulses that get created in the inter-beat series when there are false negatives or positives, these are removed using a median filter and thresholding. A combination of slope and beat amplitude information are combined using a simple nearest neighbor algorithm to determine the validity of the pulses. The final stage of the algorithm uses the IBI classification logic to address missed detections and over-detected IBI peaks. Over-detections are removed and missed detections are corrected by adding peaks from the original time series (before pre-processing). This process is repeated until the IBIs fall within an acceptable range. Finally, two rank-order filters were applied to detect any additional missed or over-detections within the heart rate limits.  **Validation:** Model was developed without training and was directly applied to a testing dataset | Manual annotation from two experts, a total of 42,539 beats were annotated by one expert, and 7,128 beats by the other. Annotators likely had access to ICP and SpO_2_ signals. | Compared to the manual annotation of 2,179 beats, the inter-rater agreement was 100% for sensitivity and PPV. Between each of the raters and the algorithm, the performance in both of these metrics was 100% | The algorithm is highly accurate in identifying valid/artifactual pulses in ABP signals.  This methodology can also be applied to ICP and SpO_2_ | IBI element of this algorithm is not efficient as it must make several passes through. |
|  | **Sex (% Male)** | N/A |  |  |  |  |  |  |  |
|  | **Age** | Pediatric | **Device type** | N/A |  |  |  |  |  |
|  | **Pathophysiological health** | PICU, 60 TBI, 60 sepsis, 90 cardiac conditions | **Location** | N/A |  |  |  |  |  |
| Asgari et al. [98] | **Subjects** | 51 from UCLA dataset and 120 from MIMIC-II [99] | **Signal type(s) used** | ABP (240 or 400 Hz), ECG | The proposed algorithm uses singular value decomposition (SVD) to validate ABP pulses. The algorithm involves the following steps:  1. The onset of pulses are detected using the method proposed for ICP beat detection by Hu et al. [151] which leverages the ECG R-wave.  2. Pulses are normalized to equal length, have zero mean, and constant variance  3. The normalized pulses make up a library that can be represented as a matrix. This matrix can then be decomposed using SVD into a signal subspace and a noise subspace  4. An incoming pulse then gets projected into the signal subspace and a noise subspace. The ratio of energy in each of these subspaces can then be compared to a threshold. This allows it to be labeled as either valid or artifactual.  In the construction of the ABP pulse library, segments of 3- to 5-minutes of data were selected from patients. These segments were processed using the morphological clustering and analysis of ICP pulse (MOCAIP) algorithm [152] to extract an ABP pulse.  **Validation:** There were 567 valid pulses that were identified by manual review. These pulses made up the aforementioned ABP library (training set, UCLA). The MIMIC-II dataset was used for validation where 18,472 ABP pulses were identified by manual review and 15,439 corresponded to valid pulses. The 90^th^ percentile of pulse lengths was used as the size for pulse normalization. | Gold-standard was clinician annotations  ABP SAI algorithm developed by Sun et al. [25]  A modified SAI method that only applies the trend-based abnormality conditions to pulses preceded by a valid beat.  A combined method using the SVD-based method to analyze the nine features extracted using the proposed SAI | Using the testing dataset for valid ABP pulse recognition:  SVD:  Acc = 0.9796  PPV = 0.9851  NPV = 0.9500  SAI:  Acc = 0.9059  PPV = 0.9506  NPV = 0.6953  Modified SAI  Acc = 0.9108  PPV = 0.9388  NPV = 0.7486  SVD projection is combined with thresholds from the SAI method:  PPV = 0.9923  NPV = 0.9500  TPR = 0.9905  FPR = 0.0392 | The modified SAI method seems to perform better than the traditional SAI  Combination of SAI and SVD methods was able to achieve a very strong PPV, decreasing the FPR and increasing the TPR. | Threshold used by SVD method is not dynamic, may need to be optimized for different populations  The hybrid SVD and SAI method had no mention of real-time applicability, potentially computationally intensive  Relies on library that may not encapsulate all possible valid pulses |
|  | **Sex (% Male)** | 68.6% |  |  |  |  |  |  |  |
|  | **Age** | 50 (14-81) | **Device type** | N/A |  |  |  |  |  |
|  | **Pathophysiological health** | Various ICP conditions (LISTED) | **Location** | N/A |  |  |  |  |  |
| Hoeksel et al. (1996) [100] | **Subjects** | 30 | **Signal type(s) used** | IBP (low-pass filtered to 12.5 Hz) | The proposed algorithm has three main components:  1. Beat detection and feature extraction – filters the signal using a low-pass third-order Butterworth filter (cutoff at 10 Hz). Detects local minima and maxima using the first order derivative. For each maximum, upstroke pressure is calculated, which is used to detect the systolic BP. Eight features are extracted from the pulmonary waveform. A beat is defined as the distance between two systoles. Mean pulmonary artery pressure (MPAP) is the average value within a beat. Additional features include the up slope, downslope, fall time, period time, and maximum first derivative.  2. Filtering of pressure beats – during ventilation, there are errors in pulse pressure and offset that must be corrected. These features can be modeled using a DC component with higher harmonics fitted using a least square fitting procedure with a weighing factor that depends on a forgetting factor. These estimated first and second harmonics can be used to correct the various features, specifically for artifacts that correspond to ventilator oscillations. The function that is used to remove the artifacts corresponding to ventilation can also be used to describe the end-expiratory value within a beat.  3. Signal validation – corrected features of a beat are used to ‘flag’ the beats. The feature values are modeled using a moving average model considering one lagged term. These values are validated using an acceptable range of their magnitude as well as the features compared to the beat model. These ranges were selected by an experienced clinician  **Validation:** Model was developed without training and was directly applied to a testing dataset | Experienced clinician made note of ‘relevant events’ in real-time | The average improvement of the different features location when comparing true beat to modeled beat included:  MPAP = 68%  SBP = 53%  DBP = 64%  dP/dt (max change in first derivative) = 28%  up slope = 40%  down slope = 38%  There were 77% ± 11% (mean ± SD) that were marked valid using the algorithm, 85% ± 13% were truly valid. | There were no false positives. All instances of coagulation, surgical manipulation, and flushes were correctly identified as artifacts by the algorithm. However, during atrial fibrillation, the algorithm marked invalid a valid segment.  Able to handle ventilation artifacts | Small number of patients included for validation  During stepwise ventilator frequency change, algorithm took approximately 3 minutes to identify the new frequency which introduced delay |
|  | **Sex (% Male)** | N/A |  |  |  |  |  |  |  |
|  | **Age** | N/A | **Device type** | 7F Swan-Ganz pulmonary catheter |  |  |  |  |  |
|  | **Pathophysiological health** | Cardiac surgery | **Location** | Jugular vein |  |  |  |  |  |
| Urteaga et al. [101] | **Subjects** | 81 | **Signal type(s) used** | IBP (250 Hz), ECG | This algorithm was designed to clean and segment the ABP signal, discerning between pulsatile and non-pulsatile segments to eventually be used to extract the heart rate and other physiological variables. It involves the following steps:   1. Preprocessing – involves a stationary wavelet transform-based denoising algorithm 2. Detection of heartbeat and delineation of signals – initially involves taking the first difference, applying a low-pass Butterworth filter, and replacing all negative values with zero. Characteristics of the differenced signals are used to identify points along the waveform, including the heartbeat. Physiological and adaptive thresholds based on past values for both the locations and relative locations of the points are used to validate the signal. 3. Derivation of variables – physiological variables are then calculated based on the validated point locations   **Validation:** Model was developed without training and was directly applied to a testing dataset | Manual annotation, likely with access to ECG signal  Was compared to other delineators developed by Zong et al. [15] and Li et al. [102] | Effectiveness for the detection of validity were calculated on the basis of the algorithm being able to identify whether a signal was pulsatile or non-pulsatile (dictated by presence of heart rate). It outperformed the Zong et al. and Li et al. methods in many metrics, by mean ± SD:  Sens = 98.8% ± 6.9%  Spec = 91.6% ± 20.2%  PPV = 97.4 ± 9.7%  NPV = 98.7 ± 6.1%  Li et al. [102]:  Sens = 99.7% ± 2.6%  Spec = 8.9% ± 23.3%  PPV = 82.4 ± 24.4%  NPV = 88.9 ± 33.3%  Zong et al. [15]:  Sens = 81.2% ± 31.0%  Spec = 75.6% ± 31.5%  PPV = 92.4 ± 20.5%  NPV = 87.7 ± 21.8% | Generally outperformed two well established methods, with the exception of underperforming slightly in sensitivity compared to Li et al. [102] | Dataset with a single recording methodology that was not validated on other patient populations  Unclear if the presence of pulsatile waveforms was sufficient to identify them as ‘valid’ |
|  | **Sex (% Male)** | N/A |  |  |  |  |  |  |  |
|  | **Age** | N/A | **Device type** | N/A |  |  |  |  |  |
|  | **Pathophysiological health** | Out-of-hospital cardiac arrests | **Location** | Radial or femoral cannulation |  |  |  |  |  |

*ABP = Arterial Blood Pressure, DBP = Diastolic Blood Pressure, dP/dt = Maximum rate of pressure change, ECG = Electrocardiogram, FPR = False Positive Rate, HR = Heart Rate, IBI = Inter-Beat Interval, IBP = Invasive Blood Pressure, ICP = Intracranial Pressure, MAP = Mean Arterial Pressure, MPAP = Mean Pulmonary Artery Pressure, NPV = Negative Predictive Value, PPV = Positive Predictive Value, PSD = Power Spectral Density, SAI = Signal Abnormality Index, SBP = Systolic Blood Pressure, SVD = Singular Value Decomposition, TPR = True Positive Rate*

**Table E.2. Dicrotic notch detection**

| **Reference** | **Subject information** | | **Data type (sampling rate) – system type** | | **Artifact removal method(s) used** | **Methods compared** | **Effectiveness** | **Study results and conclusions** | **Limitations** |
| --- | --- | --- | --- | --- | --- | --- | --- | --- | --- |
| Pal et al. [18] | **Subjects** | 4,901 patients with 352,257 4-second windows [153] | **Signal type(s) used** | IBP (256 Hz) | Uses the iterative envelope mean (IEM) method for automatic detection of the dicrotic notch (transition between systolic and diastolic BP) in the BP signal. The algorithm involves the steps:  1. Preprocessing – involves artifact removal via thresholding, employment of a filtering, and amplitude normalization.  2. IEM Method – this involves:  (i) Smoothing signal using SG filter and finding first/second derivatives of signal  (ii) Identify maxima/minima using first/second derivatives  (iii) Locations of extrema in smoothed signal are detected, and cubic spline interpolation is used to obtain upper and lower envelopes, as well as the mean envelope.  (iv) Mean envelope is subtracted from original signal to determine non-stationary part  (v) Process continues iteratively until stopping criteria indicated by the change in energy between iterations is met.  Based on the signal segments being labeled as stationary and non-stationary, the algorithm uses different aspects of their morphologies to detect the notches  **Validation:** Tested on signals with varying amounts of noise | Manual inspection  Existing second derivative method | IEM method was tested across SNRs between -30 dB and -5 dB, with increments of -1 dB, where signal was defined as the root mean squared amplitude of the non-stationary output and noise was defined as the root mean square of the stationary output.  Evaluation was based on the systolic phase’s duration (temporal distance between the onset of the systolic phase to the dicrotic notch)  There were 87,345 notches detected by manual inspection and were 100% identified by the IEM-based algorithm and the 2^nd^ derivative method.  Average detection error for ABP was 4.7 milliseconds, within the permitted range of 30 milliseconds | Low computational cost for IEM-based algorithm  Proposed method can locate DN even when not visibly identifiable  Lower error in locating cardiac cycle in IEM-based compared to 2^nd^ derivative | SG filter parameters are not adaptive, does not work well in the presence of high frequency noise  SG filter parameters defined for sampling rate  False exclusion of windows |
|  | **Sex (% Male)** | N/A |  |  |  |  |  |  |  |
|  | **Age** | N/A | **Device type** | N/A |  |  |  |  |  |
|  | **Pathophysiological health** | N/A | **Location** | N/A |  |  |  |  |  |
| Oppenheim and Sittig [103] | **Subjects** | 8 | **Signal type(s) used** | ABP (100 Hz) | The proposed method incorporates methodologies from multiple algorithms to develop an optimal dicrotic notch detection algorithm. The proposed methodology is based on the bendpoint algorithm proposed by Kinias et al. [104] and second derivative-based methods proposed by Starmer et al. [105] and Nygards et al. [106]. The flowchart of the proposed methodology includes the following steps:  Stringent prefiltering was initially conducted using Savitzky-Golay filtering.  1. A minima of the signal is determined using the methodology described in the bendpoint algorithm  2. Using the location of the identified minima, the previous point in the signal where the derivative changes from negative to positive is identified  3. This point of inflection is validated using the bendpoint algorithm considering the 5 data points before and after the identified change in direction.  The algorithm then splits off based on whether the inflection can be validated. If it can be validated, it is labeled as the dicrotic notch. If not, the following is executed:  1. The location of the systolic peak, identified using the bendpoint algorithm, is used as a starting point for all the subsequent inflections to be identified using the bendpoint algorithm.  2. Within 3 data points before and after the inflections, the first derivative is calculated to determine whether there is a direction change. If there is, this is the dicrotic notch. If not, the algorithm concludes that there is no dicrotic notch, but it is an ‘incisura’. The last inflection point before the originally identified minima is used as the first point in the segment that is searched for the location where the second derivative becomes zero. This is the identified location of the incisura.  **Validation:** Model was developed without training and was directly applied to a testing dataset | Human observers | There were 373 beats analyzed which were selected from a larger pool. There were no missed incisura, there were 2 missed dicrotic notches and 12 instances of the notches being incorrectly identified (3.75% missed or misidentification rate).  The mean error in identification for the true notches were 5.2 ± 7.5 ms (SE = 0.05) and for incisura was 8.7 ± 10.0 ms.  For all notches/incisurae, the algorithm attained a 96% accuracy with a mean error of 6.5 ± 8.8 ms. | Algorithm can identify both dicrotic notches and incisura in ABP signals with accuracy  This analysis was run retrospectively but it was indicated that it could be implemented prospectively.  It was difficult to compare this method to the Kinias, Starmer, and Nygards methods due to the differences in sampling rates impacting the temporal error in identification | Small and narrow sample size and small number of beats considered |
|  | **Sex (% Male)** | N/A |  |  |  |  |  |  |  |
|  | **Age** | N/A | **Device type** | N/A |  |  |  |  |  |
|  | **Pathophysiological health** | N/A | **Location** | N/A |  |  |  |  |  |
| Hoeksel et al. (1997) [107] | **Subjects** | 7 dogs + 50 humans | **Signal type(s) used** | **For humans:** IBP (100 Hz)  **For dogs:** IBP (200 Hz for dogs), flow probe data | The proposed algorithm was designed to detect the dicrotic notch present in the arterial blood pressure signal using arterial blood flow. Aortic blood flow is able to be estimated using blood pressure and an approximation of arterial impedance. Aortic flow is determined using aortic pressure and the Windkessel model with three parameters (characteristic input impedance, arterial compliance, and peripheral resistance). Two Windkessel models were used to model the aortic flow, one derived from arterial pressure and parameters based on literature (linear model), the other is derived from aortic pressure as well as age and gender are considered (non-linear model). The functionality of these models was validated using data from the dog dataset  The algorithm identifies the local minima and local maxima in the BP signal. It measures the distance between consecutive minima and maxima, which is referred to as the ‘upstroke’. When the upstroke exceeds 15 mmHg, it is a systolic upstroke. The minima and maxima that correspond to the upstroke are the diastole and systole, respectively. The linear and non-linear Windkessel model parameters are then used to convert the blood pressure measurement to blood flow. The dicrotic notch is determined as the first local minimum in the flow that appears after the flow turns negative. The model parameters update based on the identified location of end-diastole. The peripheral resistance parameter of a 5 second data segment was iterated 10 times to converge to a single resistance value.  The constructed models were tested using human data based off of 50 patients.  **Validation:** Model was constructed using canine data, tested using human data | For the flow estimation based on pressure, the algorithm was compared to the manually measured aortic pressure.  For dicrotic notch detection, it was compared to visual inspection by a human observer | There were two stages of performance evaluation. The first was analyzing how much flow measured from the dogs differed from that estimated based on the aortic pressure. Each 5 second interval of data was iterated over 10 times such that the peripheral resistance parameter could be optimized. There was an average of 12 beats analyzed per dog. The start and end of the ejection phases were identified using the flow probe and the pressure derived flow. The differences were 6.4 ± 4.0 ms and 5.8 ± 4.6 ms for the identified start and end of the ejection phase, respectively. The total ejection time was -0.6 ± 5.4 ms.  The linear Windkessel algorithm had a 98% agreeance rate with the visual inspection by the trained individual as the same location for the dicrotic notch was present in 49 of the 50 human patients. The non-linear Windkessel method correctly identified the notch in 96% of patients. | Dogs were used to demonstrate the feasibility of estimating aortic flow based on arterial blood pressure.  The algorithm proposed had high success in detecting the dicrotic notch; this being possibly extendable to artifact management | Accuracy of the Windkessel model, validated on small non-human dataset  Small cohort  No margin of error specified for agreement in dicrotic notch location |
|  | **Sex (% Male)** | 58% (human) |  |  |  |  |  |  |  |
|  | **Age** | 64 (40 to 80) (for humans) | **Device type** | **For dogs:** Electrogmagnetic flow probe (Skalar, Delft, the Netherlands) and micro-tip catheter (Sentron, Rhoden, the Netherlands)  **For humans:** saline-filled radial artery cannula (20-gauge) and a pres- sure transducer (Uniflow, Baxter) |  |  |  |  |  |
| Balmer et al. [108] | **Subjects** | 4 pigs (between 20 and 29 kg) | **Signal type(s) used** | IBP (1,000 Hz) | Methodologies were followed to change the stroke and waveform by applying multiple stepwise positive end-expiratory pressure (PEEP) recruitment maneuvers (RM) which is designed to minimize venous return. The dicrotic notch was attempted to be detected in both baseline and dobutamine infusion with high PEEP. The dobutamine infusion with high PEEP was expected to decrease the systolic time, widening the dicrotic notches.  The onset of the beats were detected using existing algorithm developed by Balmer et al. (2017)[154]. The shear transform algorithm is then applied. The shear line start point (SP) and shear line end points (EP) are then identified. The SP is identified using the following steps:  1. Maximum pressure is identified within a beat  2. A point on waveform is identified with the most negative gradient in a specific region of the waveform  3. The halfway point between the aforementioned point and the maximum pressure is used as the start point  For EP:  1. An initial estimate for EP is set as a point satisfying several pressure-based thresholds.  2. A line is connected between the SP and EP estimate, the shear transform is then calculated, and it is iteratively updated until it is satisfactory based on defined conditions  3. The orthogonal distance from points on the shear line to those on the pressure waveform is used to properly select the EP.  To detect the dicrotic notch:  1. The shear transform is determined between the SP and EP  2. The point of the most negative shear serves as the initial estimate for the dicrotic notch  3. Through additional tuning the final estimate of the location of the dicrotic notch is found  4. Final examination is conducted to ensure that the location of the identified points is physiologically possible.  **Validation:** Model was developed without training and was directly applied to a testing dataset | The algorithm was compared to that proposed by Kamoi et al. (2017) [109] which was used to estimate systolic time  Both algorithms were compared to the manually annotated pulses | The coefficient of determination (r^2^) between the systolic time estimated using algorithms and that which was measured. Bland-Altman analysis was also used to determine the agreement between the two. It was indicated that agreement in systolic time implied the agreement between measured and estimated dicrotic notches.  Based on the comparison of the r^2^ values for individual pigs and individua stages (PEEP or baseline), the proposed method seemed to perform better than the Kamoi et al. method. Both performed well; however, the r^2^ values was 0.99 or higher for the determination of systolic time.  The systemic error was shown by the mean differences for each method, that of the Kamoi et al. method was 11.6 ms and that of the proposed method was 0.5 ms | The proposed algorithm performed very well in identifying dicrotic notches.  Outperformed the method proposed by Kamoi et al. (2017) which was initially designed to estimate pulse wave velocity [109]. A component of this method was the identification of the end systolic point. This method also uses the maximum negative gradient of the signal to indicate end systolic pressure. The falsely detected points are reduced by applying a weight function to accentuate the end systolic pressures. | The proposed algorithm wad still somewhat error prone due to characteristics of the shear transformation and the shape of the waveform, specifically when the notch shape is flat.  There are additional potential issues with the algorithm that affect the accuracy; however, it is indicated that they are fewer than the Kamoi et al. (2017) method.  Validation only involved 80 pulses from non-human subject in only 2 hemodynamic states. |
|  | **Sex (% Male)** | N/A |  |  |  |  |  |  |  |
|  | **Age** | N/A | **Device type** | 7F pressure catheters (Transonic Sciesense Inc., Ontario, Canada) |  |  |  |  |  |
|  | **Pathophysiological health** | Administered with ketamine and diazepam, continuous infusion of sufentanil and sodium pentobarbital | **Location** | Aortic arch |  |  |  |  |  |
| Donelli et al. [110] | **Subjects** | 12 | **Signal type(s) used** | IBP (250 Hz) | The previously developed real-time dicrotic notch detection (RTDND) algorithm proposed by Hoeksel et al. [107] used a Windkessel model of blood flow to estimate aortic blood flow using aortic blood pressure. It was able to use this methodology to detect the dicrotic notch. This work discusses the extension of this algorithm to accurately predict the location of the dicrotic notch at different levels of flow, where the peak of flow (SBP) is 100%. The dicrotic notch was estimated at points of 40%, 20%, and 0% flow. The prediction of the location of the dicrotic notch is outside of the scope of this particular review; however, this work does provide further quantification of the accuracy of the RTDND algorithm to detect the dicrotic notch.  **Validation:** Model was developed without training and was directly applied to a testing dataset | Presumed to be human observers | The RTDND algorithm was able to correctly identify 98.1% or 453 of 463 dicrotic notches without any false detections | Strong performance in dicrotic notch detection, demonstrates widespread applicability of this method as it was extended to patients where HR was very high and pressure was very low | Notches were missed when the pulse-pressure ratio was very low  Small sample size |
|  | **Sex (% Male)** | 83.3% |  |  |  |  |  |  |  |
|  | **Age** | 65.8 (60.5-71.5) | **Device type** | Micromanometer transducer (F5 Sentron, CD Leycom, Zoetermeer, The Netherlands) |  |  |  |  |  |
|  | **Pathophysiological health** | Cardiac surgery with moderate to low ejection fraction | **Location** | Aortic arch |  |  |  |  |  |
| Saffarpour et al. [111] | **Subjects** | 14 pigs | **Signal type(s) used** | IBP (100 Hz) | This algorithm was designed to detect dicrotic notches in ABP signals. It involves the following steps:   1. Pre-processing – a Savitzky-Golat filter is used to initially smooth the ABP signal. Additionally, a Mayer wave is fit to the diastolic minimum points, and this portion of the signal is subtracted to remove the Mayer wave effect. 2. Cleaning using a model – a simplified cardiovascular model is constructed using three compartments (left pulsating heart, arterial systemic compartment, post-arterial systemic compartment) which are described by various parameters including blood flow and resistance. An additional term for blood backflow that causes the dicrotic notch was also included. This model is described using ordinary differential equations. 3. Optimization of model – this involves tuning the parameters of the ordinary differential equations to best model the ABP signal using a two-stage optimization that leveraged a Bayesian cost function and gradient-based optimizer. The output of this step is an accurately modeled ABP signal with reduced artifacts. 4. Rules-based logic for dicrotic notch detection – this model is inspired by the rule-based algorithm proposed by Li et al. [102] to the described cardiovascular model to determine the location of the dicrotic notch. 5. Optional use of another rule-based model to provide another estimated location of the dicrotic notch previously identified, such as that proposed by Li et al. [102]. The two estimates can be combined   There is some additional hyper tuning done in practice  **Validation:** Model was developed without training and was directly applied to a testing dataset. It was indicated that there was tuning involved but there was no specific dataset used. | Gold-standard was human identification of the dicrotic notch  Compared to pre-existing rule-based methods proposed by Li et al. [102] and Pan-Thompkins method [155] | The proposed method outperformed the Li et al. and Pan-Thompkins methods achieving an error in the detection of the dicrotic notch as 20 ± 28 milliseconds (mean ± standard deviation)  82% of the detections were within 30 milliseconds of the gold-standard dicrotic notch | The proposed method functions well at detecting the dicrotic notch, outperforming previously developed methods  Proposed method was able to function in the presence of noise | Computationally intense, requiring extensive optimization  Study conducted on pig model, under only certain pathological conditions |
|  | **Sex (% Male)** | N/A |  |  |  |  |  |  |  |
|  | **Age** | N/A | **Device type** | 5-French intra-arterial sheath and 7-French catheter |  |  |  |  |  |
|  | **Pathophysiological health** | 4 in sepsis study  10 in hemorrhage study | **Location** | Carotid artery, some threaded into proximal aorta |  |  |  |  |  |
| Singh and Sunkaria (2017) [19] | **Subjects** | 25 subjects, 5 each from 5 separate open-source datasets accessible through Physionet | **Signal type(s) used** | NIBP (250 Hz), some with ECG | The proposed algorithm attempts to locate the ‘fiducial’ points in the BP wave. The three points of interest are the pulse onset, systolic peak, and dicrotic notch. The algorithm involves the following steps:  1. Pre-processing: this is used to remove any slow wave artifacts through the application of a sixth-order Butterworth filter with the cutoff frequency of 0.4 Hz  2. Empirical wavelet transform (EWT) decomposition: initially the Fourier transformation is used to determine the frequency spectrum of the signal, boundaries are set based on the location major maxima in the spectrum, three specified modes are then extracted using an adaptive wavelet, adapted based on the boundaries.  3. Pulse onset and systolic point identification: the first maxima and zero crossing point are located in the second mode. The zero crossing points are used to define a window in which the minima represent the onset of the pulse. The maxima are used to define a window in which the maxima contained in the window are used to detect the systolic point. Thresholds in the distance between beats detected are used to detect any erroneous or missing points.  4. Dicrotic notch detection: the first order difference of the filtered signal is taken. After this, a Savitzky-Golay filter is applied to smooth the differenced signal. The locations of the onset and systolic peak are used to define a window. The maxima identified within this window of the first order differenced smoothed signal is used as a reference point to identify a minima which is the dicrotic notch. If none are detected, it is labeled as the centre between the maxima and the minima that precedes that maxima.  **Validation:** Model was developed without training and was directly applied to a testing dataset | The pulses of the outlined databases were manually annotated by a group of cardiologists  The performance of the Li et al. method was used as a comparison [102] | For the detection of systolic peak, the proposed method:  Sens = 99.95%  PPV = 99.97%  For Li et al.:  Sens = 99.92%  PPV = 99.90%  For detection of pulse onset, the proposed method:  Sens = 99.88%  PPV = 99.92%  For Li et al.:  Sens = 99.53%  PPV = 99.82%  For detection of dicrotic notch the proposed method:  Sens = 98.98%  PPV = 98.81%  For Li et al.:  Sens = 97.91%  PPV = 97.94% | The proposed algorithm has strong performance across a diverse database, outperforming an existing method | Small cohort  Potential problem posed by methodology followed to identify dicrotic notch where a midpoint is chosen between two values. |
|  | **Sex (% Male)** | N/A |  |  |  |  |  |  |  |
|  | **Age** | N/A | **Device type** | N/A |  |  |  |  |  |
|  | **Pathophysiological health** | N/A, patients sleeping | **Location** | N/A |  |  |  |  |  |
| Kinias et al. [104] | **Subjects** | 3 of 309 | **Signal type(s) used** | IBP (250 Hz) | The proposed algorithm was designed to identify critical points in the ABP signal. It uses the following methodology:  1. Pre-processing: the sampled data that was digitized was used to identify ‘critical points’, which were described as those which are the location of a change in direction of the signal as well as concavity. The locations of the ‘critical points’ are determined by first calculating the difference between every *k* and *k+n* points referred to as ΔP. Successive values of ΔP are compared to identify when the difference between values exceeds a threshold which indicates a potential change in direction of the signal. Within those identified intervals of interest, referred to as chords, a point of inflection is located through iteration. These are referred to as “bent” points.  2. Filtering: high frequency noise was able to be removed using the above method as those changes in signal direction that did not exceed a particular threshold were ignored. Additionally, the inverse of the chord length is used to remove high-frequency noise.  3. Bent points: points of relevant changes in direction are described based on their concavity and direction change, this is critical as some combinations of pairs of “bent” points are not possible based on their morphologies and can be removed. The remaining pairs of “bent” points can be labeled as upstroke points, inflection points, and downstroke points.  4. Point labelling: based on the location of the upstroke, inflection, and downstroke points, thresholds in amplitude and time are able to be used to identify the critical points including ejection onset, systolic blood pressure, dicrotic notch, and end of diastole. These thresholds ensure that the number of identified critical points are physiologically possible. There were two dicrotic notch algorithms used, one that detected the dicrotic notch as the final minimum in an outlined search zone, the other is more complex and detects the area of maximum curvature.  **Validation:** Model was developed without training and was directly applied to a testing dataset | Measured paper recording by humans | The distances between points (ejection onset to end of diastole, systolic peak to systolic peak, ejection onset to dicrotic notch, and dicrotic notch to end of diastole) were measured by both the algorithm and the human observers:  For the three patients of data observed, the mean % relative error of the cycle distances was examined (calculated using the simple dicrotic notch detection algorithm). For patient 108, there were 528 beats annotated, patient 181 there was 300, and patient 264 there was 177. The average errors (standard deviation) for each of the patients were:  Ejection to diastole = 1.74% (2.90%), 1.98% (1.96%), 0.87% (2.48%)  Systolic peak to peak = 1.53% (3.34%), 1.80% (6.65%), 0.83% (3.72%)  Ejection to dicrotic notch = 5.41% (14.10%), -6.66% (11.08%), -8.54% (16.05%)  Notch to diastole = -0.71% (8.84%), 7.71% (8.57%), 6.53% (9.61%)  The average result for the ejection to dicrotic notch was improved by a factor of 2 (for the worst segments of data) using the more complex dicrotic notch; however, it did not improve the standard deviation | Strong performance in labeling points for some patient datasets but not others  Overall, decent performance but still substantial errors prevalent.  Indicated that 1004 of 1005 beats were properly identified with only 2 false positives  Strong computational efficiency | Small sample size  Potential for errors in measurement and human errors in identifying locations of the pulses  Difficulty identifying the dicrotic notch |
|  | **Sex (% Male)** | N/A |  |  |  |  |  |  |  |
|  | **Age** | N/A | **Device type** | Saline catheter with pressure transducer |  |  |  |  |  |
|  | **Pathophysiological health** | Coronary care unit | **Location** | Axillary artery |  |  |  |  |  |
| Pachauri and Bhuyan (2012) [112] | **Subjects** | 22 signals from MGH/MF [113], [114],  14 signals from Fantasia [113], [115],  15 signals from MIT-BIH polysmographic [113], [116]  602 pulses from, CSL [44] | **Signal type(s) used** | ABP and ECG from MGH/MF (360 Hz)  ABP and ECG from MIT-BIH and Fantasia (250 Hz)  IBP from CSL (125 Hz) | This algorithm was designed to identify peaks, detect the pulse onsets, as well as detect dicrotic notches. It follows the methodology outlined below:  1. Wavelet decomposition: outlined as the ideal wavelets for this application were the sym4 (Symlets family) and db4 (Daubechies family). The wavelets were used to decompose the ABP signal.  2. Detail coefficient selection: coefficients of interest were selected for feature extraction from the signal based on energy analysis, frequency analysis, and cross-correlation analysis. Detailed coefficients corresponded to particular levels of wavelet decomposition of the ABP signal  3. Window-based thresholding: the detail coefficients were used to identify peaks in the ABP signal, important peaks were isolated from irrelevant peaks using window-based thresholding.  4. Detection of maxima: valid maxima are identified using four conditions that correspond to the relative amplitude and temporal location of the peaks, redundancies are removed  5. Peak removal: thresholds for the temporal distance between peaks as well as the amplitude of the signal are used to remove errors. The result is a series of identified systolic peaks.  6. Detection of pulse onset and dicrotic notch: the average detail coefficient at the first level of decomposition is used in conjunction with the previously identified peaks. Window-based thresholding is used to segment the ABP signals and identify minima; redundant minima are removed which were determined based on the location of the identified systolic peaks. The result of this was the onset. The onset and the systolic points were used to determine the locations of the dicrotic notches.  7. Dicrotic peak detection: this involved finding a weak peak between the dicrotic notch and the onset of the next pulse.  **Validation:** Model was developed without training and was directly applied to a testing dataset | Compared to manual annotations of the various public datasets. | The locations of the peaks, the pulse onsets, dichroitic notches, and dichroitic peaks were determined for each database, data for each of their efficacies were included in the paper. That of the MGH/MF database was used as there were 22 signals included (the most of any of the databases) where the method used the sym4 wavelet:  For 1,873 manual annotations of peak locations: Acc = 97.27%  Sens = 99.52%  PPV = 97.79%  Error = 2.67%  For 1,868 onsets manually detected: Acc = 97.96%  Sens = 99.62%  PPV = 98.36%  Error = 2.007%  For 1,860 manually identified dicrotic notches:  Acc = 98.54%  Sens = 99.35%  PPV = 99.19%  Error = 1.449% | The sym4 wavelet performed better than the db4 wavelet using the MGH/MF database; however, the db4 performed better using the MIT-BIH dataset  Strong validation of this method using data from several databases. | Only evaluated on one-minute data segments from patients due to the amount of time required to manually annotate it |
|  | **Sex (% Male)** | N/A |  |  |  |  |  |  |  |
|  | **Age** | N/A | **Device type** | N/A |  |  |  |  |  |
|  | **Pathophysiological health** | Signal with constant heart rate and arrythmias | **Location** | Aorta |  |  |  |  |  |
| Li et al. [102] | **Subjects** | 2 from CSL [44]  20 from Fantasia, accessible through Physionet [113]  16 from SLP [116] | **Signal type(s) used** | ABP (CSL) (100 Hz)  NIBP and ECG (Fantasia) (250 Hz)  IBP and ECG (SLP) (250 Hz) | The proposed algorithm was designed to detect the pulse onset, systolic blood pressure, and dicrotic notch of the arterial blood pressure signals. The raw signals are fed into the following pipeline:  1. Low-pass filtering: the raw ABP data is fed into a third order low-pass Bessel filter.  2. Derivative estimation: the first order amplitude difference is taken and is used as an estimation of the derivative of the ABP wave.  3. Estimation of thresholds: the ABP waveform is initially divided equally, a selective window is applied to the first two seconds of the ABP waveform, this window is used to adaptively update the amplitude and interval thresholds.  4. Zero-crossing points: these are located using the estimated derivative. The points of interest are the zero crosses that occur immediately before or after a maximum. These denote potential onsets and systolic points.  5. Beat evaluation: this is based on the comparison of the potential onsets and systolic points to the estimated thresholds. If they are deemed acceptable by the adaptive thresholds, the algorithm moves on. If not, the thresholds are adjusted, and this process is repeated.  6. Detecting inflection points: a searching window is used to find paired of inflection points and zero-crossing points  7. Evaluation of dicrotic notch selection: this is based off the relative location of the notch to the second inflection in the ABP pulse, as well as satisfying additional criteria.  **Validation:** Model was developed without training and was directly applied to a testing dataset | The Fantasia and SLP databases had not been annotated for their dicrotic notches, as such, these were annotated by a group of trained engineers.  Performance of previously developed methods including that of Aboy et al. [44] and Zong et al. [15] | The performance for beat detection using the CSL database (13,079 annotations) for the proposed methods:  Error = 0.34%  Sens = 99.82%  PPV = 99.84%  For Aboy et al.:  Error = 0.57%  Sens = 99.80%  PPV = 99.63%  The performance for beat detection using the Fantasia database (137,830 annotations) for the proposed methods:  Error = 1.56%  Sens = 98.29 %  PPV = 99.98%  For Zong et al.:  Error = 2.78%  Sens = 98.95%  PPV = 98.26%  The performance for beat detection using the SLP database (318,412 annotations) for the proposed methods:  Error = 0.83%  Sens = 99.19 %  PPV = 99.99%  For Zong et al.:  Error = 0.66%  Sens = 99.84%  PPV = 99.50%  A subset of the Fantasia and SLP databases were used to develop a subset database with annotation for onsets, systolic points, and dicrotic notches (2,564 annotations):  The performance for onsets, systolic points, and dicrotic notches, respectively were:  Error = 1.43%, 1.54%, 6.83%  Sens = 99.96%, 99.88%, 96.53%  PPV = 98.73%, 98.69%, 96.64%  Error was described as (FP+FN)/(TP+FP) | Strong performance in detection of beats, less strong in dicrotic notch; but overall good performance. | Relatively small cohorts |
|  | **Sex (% Male)** | N/A (all) |  |  |  |  |  |  |  |
|  | **Age** | N/A (CSL)  Youth and elders (Fantasia)  32 to 56 (SLP) | **Device type** | N/A |  |  |  |  |  |
|  | **Pathophysiological health** | N/A | **Location** | N/A |  |  |  |  |  |
| Aguirre et al.  [117] | **Subjects** | MIMIC-III (number of subjects not mentioned) [119], 2 (CSL database) [44], 20 recordings (Fantasia database (FTS)) [113], [115], 16 (Polysom-nographic database (SLP)) [113], [116] | **Signal type(s) used** | ABP (125 Hz) some with annotation using ECG | Attempts to detect different aspects of the ABP morphological signal including the SBP, DBP, and dichroitic notch referred to in this work as the fiducial points (FiP). Initially, preprocessing and data augmentation were used to prepare the data such that the proposed method could be tested on a variety of artifact types. This involved:  1. Processing of data quality by discarding obvious artifacts and examining the validity of the heartbeats to detect other possible artifacts. Potential artifacts are flagged.  2. Data augmentation by applying random replacement of beats by artifacts and adding additional baseline functions like sigmoid or sine functions. This essentially mimicked artifacts.  Two models were analyzed for their utility in this application, a U-Net model initially proposed by Ronneberger et al. [156] and an extension of the U-Net model which incorporated an 8-layer temporal convolutional network (U-TCN) initially proposed by Bai et al. [157]. The U-Net model has a ‘contracting’ path and an ‘expansive’ path [156]. The contracting path is a convolutional neural network which uses repeated unpadded convolutions, a rectified linear unit operation, and a max pooling operation to extract important features from input signal images while compressing its size [117]. The expansive path up-samples the feature map and reduces the number of feature channels using a convolution. Skip connections help facilitate a more accurate reconstruction using saved information from the contracting path in the expansive portion of the U-Net architecture. The end-result of the U-Net is the classification of a point using the final feature map. [117]  The U-TCN method was an extension of this that retained sequence information. These models classify signal segments (such as the ‘dicrotic notch to onset interval’). The changes between classes of segments mark the FiP.  **Validation:** The MIMIC-III was preprocessed, and the dataset was divided into 72.5% for training, 12.5% for validation, 15% for testing. | Annotations were provided by public datasets  Pulse delineator (PUD) method proposed by Li et al. [102] | Sensitivity, PPV, and error rate were calculated for three data sets. The CSL and FTS (annotated heartbeats using QRS wave), as well as the SLP (annotations in ECG signal simultaneously recorded). The effectiveness of the PUD, U-Net, and U-TCN methods were examined.  Across the three datasets, the best by specificity and positive predictive value was U-TCN (average of 99.83 and 99.80, respectively). This method also had the lowest error rate (average of 0.833). However, all methods performed relatively well.  However, when applied to the set of preprocessed MIMIC-III data for evaluation, the PUD method vastly underperformed the other two methods in Se, PPV, and error rate. The U-Net method achieved the best error rate value in detecting the onset, peak, and dichroitic notch with results of 0.69, 0.49, and 1.98, respectively. | The FiPs were able to be differentiated from artifacts  Competitive in the success of the proposed method using established databases | Proposed methodology requires labeled data |
|  | **Sex (% Male)** | N/A |  |  |  |  |  |  |  |
|  | **Age** | N/A | **Device type** | N/A |  |  |  |  |  |
|  | **Pathophysiological health** | Admitted to ICU | **Location** | N/A |  |  |  |  |  |
| Stevenson et al. (2012) [118] | **Subjects** | 10 pigs | **Signal type(s) used** | ABP (200 Hz) | This algorithm was proposed to extract features from aortic and pulmonary pressure waveforms such that cardiac elastance could be estimated continuously. The shear transform was used for feature identification from aortic and pulmonary pressure waveforms. These features include the pulse maximum, pulse minimum, left shoulder, right shoulder, maximum positive gradient, maximum negative gradient as well as two more difficult features including the driver maximum positive gradient (DMPG) and dicrotic notch. This method uses signal morphology to detect the aforementioned features of interest. All but the pulse minimum and right shoulder were extracted.  **Validation:** Training set composed of 51 waveforms from 5 pigs, validation set composed of 37 waveforms from 5 pigs | Manual identification of features | There were 616 features to be identified from the recorded signals. This algorithm was able to correctly identify 605 points within 1% accuracy, 5 points within 5% accuracy, 4 within 10% accuracy, and 2 within 20% accuracy.  DN was able to be identified within 1% error in 87 of 88 waveforms between development and validation sets | Proposed methodology is indicated as being robust to noise  Able to identify features with good accuracy | Study was conducted on a small cohort of non-human subjects |
|  | **Sex (% Male)** | N/A |  |  |  |  |  |  |  |
|  | **Age** | N/A | **Device type** | Catheter |  |  |  |  |  |
|  | **Pathophysiological health** | 5 with pulmonary embolism, 5 with septic shock | **Location** | Aorta, pulmonary artery |  |  |  |  |  |

*ABP = Arterial Blood Pressure, DBP = Diastolic Blood Pressure, ECG = Electrocardiogram, EDSS = End Diastole Slope Sum, EWT = Empirical Wavelet Transform, FiP = Fiducial Points, HR = Heart Rate, IBP = Invasive Blood Pressure, IEM = Iterative Envelope Mean, MAA = Maximum Amplitude Algorithm, MAP = Mean Arterial Pressure, MPPS = Maximum Positive Pressure Slope, MUSD = Maximum Up-Slope Duration, OWE = Oscillometric Waveform Envelope, PEEP = Positive End-Expiratory Pressure, PP = Pulse Pressure, PPV = Positive Predictive Value, PUD = Pulse Delineator, RM = Recruitment Maneuver, RTDND = Real-Time Dicrotic Notch Detection, SAI = Signal Abnormality Index, SBP = Systolic Blood Pressure, SE = Standard Error, SESS = Slow Ejection Slope Sum, SG = Savitzky-Golay, SNR = Signal-to-Noise Ratio, SVR = Support Vector Regression, U-TCN = U-Net with Temporal Convolutional Network*

**Table E.3. Pulse onset detection**

| **Reference** | **Subject information** | | **Data type (sampling rate) – system type** | | **Artifact removal method(s) used** | **Methods compared** | **Effectiveness** | **Study results and conclusions** | **Limitations** |
| --- | --- | --- | --- | --- | --- | --- | --- | --- | --- |
| Zong et al. (2003) [15] | **Subjects** | 18 from MIT-BIH Polyso-mnographic Database [113], [116], additional 10 from Heldt et al. study [158] | **Signal type(s) used** | IBP, ECG [113], [116]  NIBP, ECG [158]  Sampling frequency is not explicitly stated but 250 Hz is discussed | The proposed algorithm was designed to identify pulses in ABP signals and identify the location of the pulse onset specifically. The algorithm has three sequentially applied steps:  1. Apply a second order recursive filter with an approximate cutoff at 16 Hz.  2. Implement a slope sum function (SSF) which essentially magnifies instances of upward slope in an ABP pulse and minimizes the rest of the pulse. The window sizes used was 128 ms.  3. Adaptive thresholding is used on the SSF to detect pulses in the signal. When a pulse is detected, the 150 ms of data before and after the pulse is examined to determine whether the difference between the minimum and maximum in the SSF is sufficient to be able to detect the pulse. The threshold crossing point is then used to determine the onset of the pulse, it is listed as when the signal reaches 1% of the maximum pulse value. The signal is then adjusted for the delay induced by the low-pass filter and a refractory period is also introduced to avoid duplicate detection.  **Validation:** Model was developed without training and was directly applied to a testing dataset | Human annotations, likely with access to ECG signals.    For the pulse detection, ECG-based annotations were used [113], [116]  For pulse onset detection, human annotations on ECG and NIBP were used [158] | For pulse detection using ECG-based annotations:  Sens = 99.31% (gross), 99.26% (avg)  Positive predictive accuracy (PPA) = 99.74% (gross), 99.77% (average)  For pulse detection compared to human annotated NIBP dataset:  Sens = 99.71% (gross), 99.71% (avg)  PPA = 99.69% (gross), 99.72% (average).  For pulse onset detection, histograms were plotted to depict the error as a function of time compared to reference annotations, for 96.41% of the 39,848 beats that were analyzed, there error was less than or equal to 20 ms. | Accurate detection of pulses and pulse onsets using the developed method | Small sample size  Little information regarding the pathophysio-logy of the patients included |
|  | **Sex (% Male)** | N/A [113], [116]  50% [97] |  |  |  |  |  |  |  |
|  | **Age** | N/A [113], [116]  28.7 ± 1.2 years [158] | **Device type** | N/A [113], [116]  Finapres [158] |  |  |  |  |  |
| Singh and Sunkaria (2017) [19] | **Subjects** | 25 subjects, 5 each from 5 separate open-source datasets accessible through Physionet | **Signal type(s) used** | NIBP (250 Hz) | The proposed algorithm attempts to locate the ‘fiducial’ points in the BP wave. The three points of interest are the pulse onset, systolic peak, and dicrotic notch. The algorithm involves the following steps:  1. Pre-processing: this is used to remove any slow wave artifacts through the application of a sixth-order Butterworth filter with the cutoff frequency of 0.4 Hz  2. Empirical wavelet transform (EWT) decomposition: initially the Fourier transformation is used to determine the frequency spectrum of the signal, boundaries are set based on the location major maxima in the spectrum, three specified modes are then extracted using an adaptive wavelet, adapted based on the boundaries.  3. Pulse onset and systolic point identification: the first maxima and zero crossing point are located in the second mode. The zero crossing points are used to define a window in which the minima represents the onset of the pulse. The maxima are used to define a window in which the maxima contained in the window are used to detect the systolic point. Thresholds in the distance between beats detected are used to detect any erroneous or missing points.  4. Dicrotic notch detection: the first order difference of the filtered signal is taken. After this, a Savitzky-Golay filter is applied to smooth the differenced signal. The locations of the onset and systolic peak are used to define a window. The maxima identified within this window of the first order differenced smoothed signal is used as a reference point to identify a minima which is the dicrotic notch. If none are detected, it is labeled as the centre between the maxima and the minima that precedes that maxima.  **Validation:** Model was developed without training and was directly applied to a testing dataset | The pulses of the outlined databases were manually annotated by a group of cardiologists  The performance of the Li et al. method was used as a comparison [102] | For the detection of systolic peak the proposed method:  Sens = 99.95%  PPV = 99.97%  For Li et al.:  Sens = 99.92%  PPV = 99.90%  For detection of pulse onset the proposed method:  Sens = 99.88%  PPV = 99.92%  For Li et al.:  Sens = 99.53%  PPV = 99.82%  For detection of dicrotic notch the proposed method:  Sens = 98.98%  PPV = 98.81%  For Li et al.:  Sens = 97.91%  PPV = 97.94% | The proposed algorithm has strong performance across a diverse database, outperforming an existing method | Small cohort  Potential problem posed by methodology followed to identify dicrotic notch where a midpoint is chosen between two values. |
|  | **Sex (% Male)** | N/A |  |  |  |  |  |  |  |
|  | **Age** | N/A | **Device type** | N/A |  |  |  |  |  |
|  | **Pathophysiological health** | N/A, patients sleeping | **Location** | N/A |  |  |  |  |  |
| Pachauri and Bhuyan (2012) [112] | **Subjects** | 22 signals from MGH/MF [113], [114],  14 signals from Fantasia [113], [115],  15 signals from MIT-BIH polysmographic [113], [116]  602 pulses from, CSL [44] | **Signal type(s) used** | ABP and ECG from MGH/MF (360 Hz)  ABP and ECG from MIT-BIH and Fantasia (250 Hz)  IBP from CSL (125 Hz) | This algorithm was designed to identify peaks, detect the pulse onsets, as well as detect dicrotic notches. It follows the methodology outlined below:  1. Wavelet decomposition: outlined as the ideal wavelets for this application were the sym4 (Symlets family) and db4 (Daubechies family). The wavelets were used to decompose the ABP signal.  2. Detail coefficient selection: coefficients of interest were selected for feature extraction from the signal based on energy analysis, frequency analysis, and cross-correlation analysis. Detailed coefficients corresponded to particular levels of wavelet decomposition of the ABP signal  3. Window-based thresholding: the detail coefficients were used to identify peaks in the ABP signal, important peaks were isolated from irrelevant peaks using window-based thresholding.  4. Detection of maxima: valid maxima are identified using four conditions that correspond to the relative amplitude and temporal location of the peaks, redundancies are removed  5. Peak removal: thresholds for the temporal distance between peaks as well as the amplitude of the signal are used to remove errors. The result is a series of identified systolic peaks.  6. Detection of pulse onset and dicrotic notch: the average detail coefficient at the first level of decomposition is used in conjunction with the previously identified peaks. Window-based thresholding is used to segment the ABP signals and identify minima; redundant minima are removed which were determined based on the location of the identified systolic peaks. The result of this was the onset. The onset and the systolic points were used to determine the locations of the dicrotic notches.  7. Dicrotic peak detection: this involved finding a weak peak between the dicrotic notch and the onset of the next pulse.  **Validation:** Model was developed without training and was directly applied to a testing dataset | Compared to manual annotations of the various datasets, some of which had access to ECG. | The locations of the peaks, the pulse onsets, dichroitic notches, and dichroitic peaks were determined for each database, data for each of their efficacies were included in the paper. That of the MGH/MF database was used as there were 22 signals included (the most of any of the databases) where the method used the sym4 wavelet:  For 1,873 manual annotations of peak locations: Acc = 97.27%  Sens = 99.52%  PPV = 97.79%  Error = 2.67%  For 1,868 onsets manually detected: Acc = 97.96%  Sens = 99.62%  PPV = 98.36%  Error = 2.007%  For 1,860 manually identified dicrotic notches:  Acc = 98.54%  Sens = 99.35%  PPV = 99.19%  Error = 1.449%  For 1,860 manually identified dicrotic peaks:  Acc = 97.58%  Sens = 98.05%  PPV = 99.50%  Error = 2.46%  Acc = (1-(errors/annotation) x 100 | The sym4 wavelet performed better than the db4 wavelet using the MGH/MF database; however, the db4 performed better using the MIT-BIH dataset  Strong validation of this method using data from several databases. | Only evaluated on one-minute data segments from patients due to the amount of time required to manually annotate it |
|  | **Sex (% Male)** | N/A |  |  |  |  |  |  |  |
|  | **Age** | N/A | **Device type** | N/A |  |  |  |  |  |
|  | **Pathophysiological health** | Signal with constant heart rate and arrythmias | **Location** | Aorta |  |  |  |  |  |
| Li et al. [102] | **Subjects** | 2 from CSL [44]  20 from Fantasia, accessible through Physionet [113]  16 from SLP [116] | **Signal type(s) used** | ABP (CSL) (100 Hz)  NIBP and ECG (Fantasia) (250 Hz)  IBP and ECG (SLP) (250 Hz) | The proposed algorithm was designed to detect the pulse onset, systolic blood pressure, and dicrotic notch of the arterial blood pressure signals. The raw signals are fed into the following pipeline:  1. Low-pass filtering: the raw ABP data is fed into a third order low-pass Bessel filter.  2. Derivative estimation: the first order amplitude difference is taken and is used as an estimation of the derivative of the ABP wave.  3. Estimation of thresholds: the ABP waveform is initially divided equally, a selective window is applied to the first two seconds of the ABP waveform, this window is used to adaptively update the amplitude and interval thresholds.  4. Zero-crossing points: these are located using the estimated derivative. The points of interest are the zero crosses that occur immediately before or after a maximum. These denote potential onsets and systolic points.  5. Beat evaluation: this is based on the comparison of the potential onsets and systolic points to the estimated thresholds. If they are deemed acceptable by the adaptive thresholds, the algorithm moves on. If not, the thresholds are adjusted, and this process is repeated.  6. Detecting inflection points: a searching window is used to find paired of inflection points and zero-crossing points  7. Evaluation of dicrotic notch selection: this is based off the relative location of the notch to the second inflection in the ABP pulse, as well as satisfying additional criteria.  **Validation:** Model was developed without training and was directly applied to a testing dataset | The Fantasia and SLP databases had not been annotated for their dicrotic notches, as such, these were annotated by a group of trained engineers, some of the datasets had simultaneously recorded ECG signals.  Performance of previously developed methods including that of Aboy et al. [44] and Zong et al. [15] | The performance for beat detection using the CSL database (13,079 annotations) for the proposed methods:  Error = 0.34%  Sens = 99.82%  PPV = 99.84%  For Aboy et al.:  Error = 0.57%  Sens = 99.80%  PPV = 99.63%  The performance for beat detection using the Fantasia database (137,830 annotations) for the proposed methods:  Error = 1.56%  Sens = 98.29 %  PPV = 99.98%  For Zong et al.:  Error = 2.78%  Sens = 98.95%  PPV = 98.26%  The performance for beat detection using the SLP database (318,412 annotations) for the proposed methods:  Error = 0.83%  Sens = 99.19 %  PPV = 99.99%  For Zong et al.:  Error = 0.66%  Sens = 99.84%  PPV = 99.50%  A subset of the Fantasia and SLP databases were used to develop a subset with annotation for onsets, systolic points, and dicrotic notches (2,564 annotations):  The performance for onsets, systolic points, and dicrotic notches, respectively were:  Error = 1.43%, 1.54%, 6.83%  Sens = 99.96%, 99.88%, 96.53%  PPV = 98.73%, 98.69%, 96.64% | Strong performance in detection of beats, less strong in dicrotic notch; but overall good performance. | Relatively small cohorts |
|  | **Sex (% Male)** | N/A (all) |  |  |  |  |  |  |  |
|  | **Age** | N/A (CSL)  Youth and elders (Fantasia)  32 to 56 (SLP) | **Device type** | N/A |  |  |  |  |  |
|  | **Pathophysiological health** | N/A | **Location** | N/A |  |  |  |  |  |
| Aguirre et al.  [117] | **Subjects** | MIMIC-III (number of subjects not mentioned) [119], 2 (CSL database) [44], 20 recordings (Fantasia database (FTS)) [113], [115], 16 (Polysom-nographic database (SLP)) [113], [116] | **Signal type(s) used** | ABP (125 Hz) some with annotation using ECG | Attempts to detect different aspects of the ABP morphological signal including the SBP, DBP, and dichroitic notch referred to in this work as the fiducial points (FiP). Initially, preprocessing and data augmentation were used to prepare the data such that the proposed method could be tested on a variety of artifact types. This involved:  1. Processing of data quality by discarding obvious artifacts and examining the validity of the heartbeats to detect other possible artifacts. Potential artifacts are flagged.  2. Data augmentation by applying random replacement of beats by artifacts and adding additional baseline functions like sigmoid or sine functions. This essentially mimicked artifacts.  Two models were analyzed for their utility in this application, a U-Net model initially proposed by Ronneberger et al. [156] and an extension of the U-Net model which incorporated an 8-layer temporal convolutional network (U-TCN) initially proposed by Bai et al. [157]. The U-Net model has a ‘contracting’ path and an ‘expansive’ path [156]. The contracting path is a convolutional neural network which uses repeated unpadded convolutions, a rectified linear unit operation, and a max pooling operation to extract important features from input signal images while compressing its size [117]. The expansive path up-samples the feature map and reduces the number of feature channels using a convolution. Skip connections help facilitate a more accurate reconstruction using saved information from the contracting path in the expansive portion of the U-Net architecture. The end-result of the U-Net is the classification of a point using the final feature map. [117]  The U-TCN method was an extension of this that retained sequence information. These models classify signal segments (such as the ‘dicrotic notch to onset interval’). The changes between classes of segments mark the FiP.  **Validation:** The MIMIC-III was preprocessed, and the dataset was divided into 72.5% for training, 12.5% for validation, 15% for testing. | Annotations were provided by public datasets  Pulse delineator (PUD) method proposed by Li et al. [102] | Sensitivity, PPV, and error rate were calculated for three data sets. The CSL and FTS (annotated heartbeats using QRS wave), as well as the SLP (annotations in ECG signal simultaneously recorded). The effectiveness of the PUD, U-Net, and U-TCN methods were examined.  Across the three datasets, the best by specificity and positive predictive value was U-TCN (average of 99.83 and 99.80, respectively). This method also had the lowest error rate (average of 0.833). However, all methods performed relatively well.  However, when applied to the set of preprocessed MIMIC-III data for evaluation, the PUD method vastly underperformed the other two methods in Se, PPV, and error rate. The U-Net method achieved the best error rate value in detecting the onset, peak, and dichroitic notch with results of 0.69, 0.49, and 1.98, respectively. | The FiPs were able to be differentiated from artifacts  Competitive in the success of the proposed method using established databases | Proposed methodology requires labeled data |
|  | **Sex (% Male)** | N/A |  |  |  |  |  |  |  |
|  | **Age** | N/A | **Device type** | N/A |  |  |  |  |  |
|  | **Pathophysiological health** | Admitted to ICU | **Location** | N/A |  |  |  |  |  |
| Xu et al. (2009) [120] | **Subjects** | 16 | **Signal type(s) used** | For SAH: IBP, ECG, ICP, CBFV  For NPH: IBP, ICP | This algorithm aims to enhance signal quality and detect pulse onset detection in the presence of noise or artifacts.  1. Signal enhancement – a signal matrix (S) is constructed using the two adjacent pulses (neighboring) for each k-th pulse in length L which is the minimum interval length from the QRS peak to the peaks of the three pulses, which can be found using ECG when applicable. PCA is then applied using robust SVD where the first component embodies the fundamental pulsatile information.  2. Pulse onset detection – three definitions of the pulse onsets involved the diastolic point, maximal second derivative, and tangent intersection. The diastolic point is detected by segmenting the interval between the QRS peak and the pulse upstroke and fitting a cubic spline curve. The corresponding minimum is the diastolic point. The maximum second derivative approach. This approach involves using the same interval as the previous method. A 5-point central difference formula is used to calculate the second order derivative, where the maximum second order derivative indicates the location of the pulse onset. The tangent intersection point method was based off a work from Chiu et al. [159] which involves iteratively determining the maximum first derivative within the interval and having a horizontal line drawn from the diastolic point and having the intersection of the lines indicating the pulse onset.  **Validation:** Model was developed without training and was directly applied to a testing dataset | With and without the signal enhancement was applied | Effectiveness was calculated using the mean and standard deviation variance of the difference between the pulse onset detected from the original signal to that of the filtered signal.  For ICP (ms):  Diastolic point: 2.89 ± 6.80  Max. second derivative: 2.28 ± 9.43  Tangent intersection point: 1.14 ± 3.11  For ABP (ms):  Diastolic point: 1.15 ± 6.02  Max. second derivative: 2.23 ± 12.43  Tangent intersection point: 2.85 ± 5.59  For CBFV (ms):  Diastolic point: 4.40 ± 8.63  Max. second derivative: 7.79 ± 22.7  Tangent intersection point: 2.05 ± 5.59 | Across all three methods of pulse onset detection, the use of the signal enhancement method improved the detection accuracy in the presence of noise.  In the presence of more noise, the accuracy of the pulse onset detection decreased.  There were differences in the successfulness of the methods regardless of the application of the signal enhancement | Derivative-based methods were more sensitive to the presence of noise  Relies on ECG, unclear as to how this was tested on patients without ECG present  Not clear distinction between which patients were used and if there were any differences noted between the groups/signal types  No ’gold-standard’ for identification based on human |
|  | **Sex (% Male)** | N/A |  |  |  |  |  |  |  |
|  | **Age** | N/A | **Device type** | CBFV measured using a transcranial Doppler ultrasound device (Vuasys, Madision))  IBP for SAH, and NIBP (Collin Biomedical, Japan) for NPH  ICP measured using (intraparenchymal microsensor (Codman and Schurtleff)  All digitized at 400 Hz. |  |  |  |  |  |
|  | **Pathophysiological health** | SAH and NPH |  |  |  |  |  |  |  |
| Kyle et al. [121] | **Subjects** | 350 | **Signal type(s) used** | ABP (Brachial pulse) (250 Hz) | This algorithm was designed to detect particular aspects of the brachial pulse signal including the beginning of the pulse (F), systolic peak (P), “incisura” (inflection at the end of the systolic phase) (I), dicrotic peak (local maximum after incisura)(D). Each pulse wave is processed and was only considered successfully processed if it had no artifacts and the algorithm was able to detect the P, I, and D points. The algorithm had the following steps:  1. Find the upslope that corresponds to systole for each of the pulse cycles using a threshold for the first difference. The threshold is adjusted if there are too few upslopes identified  2. Determine the location of the minimum for the slope and find the F point  3. The maximum point of the wave is set as point P  4. Find the downslope corresponding to the end of systole based on a threshold for the first difference.  5. Find onset of dicrotic wave using the next upslope, the local minimum is point I and the local maximum is point D. Variations in the pulse wave are handled using particular thresholds based on its time relative location to the F point. If it is not able to be found, the location is estimated using the change of slope method.  There were minimum and maximum thresholds for the distance between the F and P, F and I, and I and D points. If they were not within an acceptable range, they were discarded.  Any baseline drift is removed through detection using a line drawn through the minima of the slopes. Amplitudes of the P point, I point, and D point are calculated as well as the ratios between the amplitudes of the I and P points as well as the I and D points.  **Validation:** Model was developed without training and was directly applied to a testing dataset | Human observers | For each cycle there were several metrics calculated, and their results were compared to those generated by human observers. These included distance between F-F points, F-P points as a percentage of the total pulse, F-I points as a percentage of the total pulse, and I-D points as well as the previously mentioned amplitude ratios. Correlation (r) was calculated for the scatter plots of the results for the computer against the human observer. The results were as follow:  F-F: r=0.99  F-P (%): r=0.97  I/P: r=0.97  F-I (%): r=0.94  I/D: r=0.94  Algorithm was able to process 332 of 350 records | Good performance compared to human observers, discarding artifacts improved accuracy  Can be extended to intra-arterial signals | Sources of error associated with poor recording quality leading to an inability for the algorithm to properly detect all points  Population used was quite specific  Point D was estimated when it could not be directly identified |
|  | **Sex (% Male)** | 100% |  |  |  |  |  |  |  |
|  | **Age** | 15 to 88 | **Device type** | Pressure transducer |  |  |  |  |  |
|  | **Pathophysiological health** | Normal as well as with disease including hypertension, atherosclerosis, and diabetes | **Location** | Brachial artery |  |  |  |  |  |
| Singh and Sunkaria (2016) [122] | **Subjects** | 5 to 10 clean recordings selected (MIT-BIH Polysomno-graphic [113], [116])  40 (Fantasia [113], [115])  90 (MIMIC [113], [123]) | **Signal type(s) used** | ABP;  Invasiveness not indicated for MIT-BIH  Non-invasive for Fantasia (250 Hz)  Assumed to be invasive MIMIC | This algorithm uses the empirical wavelet transformation (EWT) to detect the onsets and systolic peaks of ABP data. The EWT is used to decompose the ABP signal into 5 different ‘modes’ (frequency components). The mode that aligns closest with the oscillations of the blood pressure is used for delineation of the waveforms. This is determined by comparing the central frequencies. The zero crossings of the identified mode are expected to align with the onsets of the blood pressure waveform. The maxima of the identified mode are expected to align with the systolic peaks. To precisely determine the location of either point of interest, a local search was conducted in the vicinity of the identified zero crossing and maxima in the identified mode. The minima of the ABP signal in the region of the zero crossing and the maxima in the region of the mode maxima were recorded as the final onset and systolic peak, respectively.  **Validation:** Model was developed without training and was directly applied to a testing dataset | Expert annotations provided by public datasets | For MIT-BIH, Fantasia, MIMIC, respectively:  Sens = 99.92%, 100%, 99.93% (onsets)  Sens = 99.96%, 99.97%, 99.95% (systolic peaks)  Error = 0.15%, 0.02%, 0.18% (onsets)  Error = 0.15%, 0.05%, 0.12% (systolic peaks)  PPV = 99.92%, 99.97%, 99.87% (onsets)  PPV = 99.88%, 99.97%, 99.91% (systolic peaks) | Algorithm achieved strong performance in identification of morphological features of ABP pulses  Used diverse dataset  Could be used for further waveform delineation for identification of more features | All data used was identified as being clean data  Only identifies two features of waveform |
|  | **Sex (% Male)** | N/A (MIT-BIH)  50% (Fantasia)  N/A (MIMIC) |  |  |  |  |  |  |  |
|  | **Age** | N/A (MIT-BIH)  20 between 21 and 34 years, 20 between 68 to 85 years (Fantasia)  N/A (MIMIC) | **Device type** | N/A |  |  |  |  |  |
|  | **Pathophysiological health** | Chronic obstructive sleep apnea (MIT-BIH), healthy (Fantasia), ICU (MIMIC) | **Location** | N/A |  |  |  |  |  |
| Lee et al. (2018) [124] | **Subjects** | 10 from MIMIC [113] | **Signal type(s) used** | IBP (125 Hz) | The method outline has the following steps:  1. Calculation of sampling frequency such that it is constant using the Monte Carlo method which involves randomly selecting points along the signal and iteratively calculating the number of data points in a second. Outliers are removed using percentile-based thresholding,  2. Systolic peak detection is performed by locating a local maximum first using a 1.5 second window and then a 0.1 second window, removing any redundant detections.  3. Pulse onset detection involves using the gradient to determine at what point between two systolic peaks that the slope becomes zero.  **Validation:** Model was developed without training and was directly applied to a testing dataset | Manually annotated data provided by public databases | Tested using the MIMIC database [113] with 22,544 pulses (approximately 10% artifacts) initially filtered using a low-pass filter with a cut-off at 5 Hz.  Average mean error of pulse onset detection was approximately 2.4μs and the standard deviation was approximately 12.5μs. The difference between real onsets and detected onsets is within 3% for each patient. The average percentage difference between the real and detected onsets was approximately 0.95%. | This method functions well even in the presence of motion or noise  Performed superior to two different other methodologies; however, these two studies did not discuss ABP detection. | Small cohort  Unsure as to the functionality under different ABP morphologies than it was tested on. |
|  | **Sex (% Male)** | N/A |  |  |  |  |  |  |  |
|  | **Age** | N/A | **Device type** | N/A |  |  |  |  |  |
|  | **Pathophysiological health** | TBI | **Location** | N/A |  |  |  |  |  |

*ABP = Arterial Blood Pressure, CBFV = Cerebral Blood Flow Velocity, DBP = Diastolic Blood Pressure, ECG = Electrocardiogram, EWT = Empirical Wavelet Transform, FiP = Fiducial Points, FTS = Fantasia Database, HR = Heart Rate, IBP = Invasive Blood Pressure, ICP = Intracranial Pressure, LVET = Left Ventricular Ejection Time, MAP = Mean Arterial Pressure, MGH/MF = Massachusetts General Hospital/Marquette Foundation Database, MIT-BIH = Massachusetts Institute of Technology - Beth Israel Hospital Database, NIBP = Non-Invasive Blood Pressure, NPH = Normal Pressure Hydrocephalus, PEP = Pre-Ejection Period, PPV = Positive Predictive Value, PPA = Positive Predictive Accuracy, PUD = Pulse Delineator, QS = Electromechanical Systole, SAH = Subarachnoid Hemorrhage, SG = Savitzky-Golay, SSF = Slope Sum Function, U-Net = Convolutional Neural Network with Encoder-Decoder and Skip Connections, U-TCN = U-Net with Temporal Convolutional Network*

**Table E.4. Systolic peak detection**

| **Reference** | **Subject information** | | **Data type (sampling rate) – system type** | | **Artifact removal method(s) used** | **Methods compared** | **Effectiveness** | **Study results and conclusions** | **Limitations** |
| --- | --- | --- | --- | --- | --- | --- | --- | --- | --- |
| Singh and Sunkaria (2017) [19] | **Subjects** | 25 subjects, 5 each from 5 separate open-source datasets accessible through Physionet | **Signal type(s) used** | NIBP (250 Hz) | The proposed algorithm attempts to locate the ‘fiducial’ points in the BP wave. The three points of interest are the pulse onset, systolic peak, and dicrotic notch. The algorithm involves the following steps:  1. Pre-processing: this is used to remove any slow wave artifacts through the application of a sixth-order Butterworth filter with the cutoff frequency of 0.4 Hz  2. Empirical wavelet transform (EWT) decomposition: initially the Fourier transformation is used to determine the frequency spectrum of the signal, boundaries are set based on the location major maxima in the spectrum, three specified modes are then extracted using an adaptive wavelet, adapted based on the boundaries.  3. Pulse onset and systolic point identification: the first maxima and zero crossing point are located in the second mode. The zero crossing points are used to define a window in which the minima represents the onset of the pulse. The maxima are used to define a window in which the maxima contained in the window are used to detect the systolic point. Thresholds in the distance between beats detected are used to detect any erroneous or missing points.  4. Dicrotic notch detection: the first order difference of the filtered signal is taken. After this, a Savitzky-Golay filter is applied to smooth the differenced signal. The locations of the onset and systolic peak are used to define a window. The maxima identified within this window of the first order differenced smoothed signal is used as a reference point to identify a minima which is the dicrotic notch. If none are detected, it is labeled as the centre between the maxima and the minima that precedes that maxima.  **Validation:** Model was developed without training and was directly applied to a testing dataset | The pulses of the outlined databases were manually annotated by a group of cardiologists  The performance of the Li et al. method was used as a comparison [102] | For the detection of systolic peak, the proposed method:  Sens = 99.95%  PPV = 99.97%  For Li et al.:  Sens = 99.92%  PPV = 99.90%  For detection of pulse onset, the proposed method:  Sens = 99.88%  PPV = 99.92%  For Li et al.:  Sens = 99.53%  PPV = 99.82%  For detection of dicrotic notch the proposed method:  Sens = 98.98%  PPV = 98.81%  For Li et al.:  Sens = 97.91%  PPV = 97.94% | The proposed algorithm has strong performance across a diverse database, outperforming an existing method | Small cohort  Potential problem posed by methodology followed to identify dicrotic notch where a midpoint is chosen between two values. |
|  | **Sex (% Male)** | N/A |  |  |  |  |  |  |  |
|  | **Age** | N/A | **Device type** | N/A |  |  |  |  |  |
|  | **Pathophysiological health** | N/A, patients sleeping | **Location** | N/A |  |  |  |  |  |
| Kinias et al. [104] | **Subjects** | 3 of 309 | **Signal type(s) used** | IBP (250 Hz) | The proposed algorithm was designed to identify critical points in the ABP signal. It uses the following methodology:  1. Pre-processing: the sampled data that was digitized was used to identify ‘critical points’, which were described as those which are the location of a change in direction of the signal as well as concavity. The locations of the ‘critical points’ are determined by first calculating the difference between every *k* and *k+n* points referred to as ΔP. Successive values of ΔP are compared to identify when the difference between values exceeds a threshold which indicates a potential change in direction of the signal. Within those identified intervals of interest, referred to as chords, a point of inflection is located through iteration. These are referred to as “bent” points.  2. Filtering: high frequency noise was able to be removed using the above method as those changes in signal direction that did not exceed a particular threshold were ignored. Additionally, the inverse of the chord length is used to remove high-frequency noise.  3. Bent points: points of relevant changes in direction are described based on their concavity and direction change, this is critical as some combinations of pairs of “bent” points are not possible based on their morphologies and can be removed. The remaining pairs of “bent” points can be labeled as upstroke points, inflection points, and downstroke points.  4. Point labelling: based on the location of the upstroke, inflection, and downstroke points, thresholds in amplitude and time are able to be used to identify the critical points including ejection onset, systolic blood pressure, dicrotic notch, and end of diastole. These thresholds ensure that the number of identified critical points are physiologically possible. There were two dicrotic notch algorithms used, one that detected the dicrotic notch as the final minimum in an outlined search zone, the other is more complex and detects the area of maximum curvature.  **Validation:** Model was developed without training and was directly applied to a testing dataset | Measured paper recording by humans | The distances between points (ejection onset to end of diastole, systolic peak to systolic peak, ejection onset to dicrotic notch, and dicrotic notch to end of diastole) were measured by both the algorithm and the human observers:  For the three patients of data observed, the mean % relative error of the cycle distances was examined (calculated using the simple dicrotic notch detection algorithm). For patient 108, there were 528 beats annotated, patient 181 there was 300, and patient 264 there was 177. The average errors (standard deviation) for each of the patients were:  Ejection to diastole = 1.74% (2.90%), 1.98% (1.96%), 0.87% (2.48%)  Systolic peak to peak = 1.53% (3.34%), 1.80% (6.65%), 0.83% (3.72%)  Ejection to dicrotic notch = 5.41% (14.10%), -6.66% (11.08%), -8.54% (16.05%)  Notch to diastole = -0.71% (8.84%), 7.71% (8.57%), 6.53% (9.61%)  The average result for the ejection to dicrotic notch was improved by a factor of 2 (for the worst segments of data) using the more complex dicrotic notch; however, it did not improve the standard deviation | Strong performance in labeling points for some patient datasets but not others  Overall, decent performance but still substantial errors prevalent.  Indicated that 1004 of 1005 beats were properly identified with only 2 false positives  Strong computational efficiency | Small sample size  Potential for errors in measurement and human errors in identifying locations of the pulses  Difficulty identifying the dicrotic notch |
|  | **Sex (% Male)** | N/A |  |  |  |  |  |  |  |
|  | **Age** | N/A | **Device type** | Saline catheter with pressure transducer |  |  |  |  |  |
|  | **Pathophysiological health** | Coronary care unit | **Location** | Axillary artery |  |  |  |  |  |
| Pachauri and Bhuyan (2012) [112] | **Subjects** | 22 signals from MGH/MF [113], [114],  14 signals from Fantasia [113], [115],  15 signals from MIT-BIH polysmographic [113], [116]  602 pulses from, CSL [44] | **Signal type(s) used** | ABP and ECG from MGH/MF (360 Hz)  ABP and ECG from MIT-BIH and Fantasia (250 Hz)  IBP from CSL (125 Hz) | This algorithm was designed to identify peaks, detect the pulse onsets, as well as detect dicrotic notches. It follows the methodology outlined below:  1. Wavelet decomposition: outlined as the ideal wavelets for this application were the sym4 (Symlets family) and db4 (Daubechies family). The wavelets were used to decompose the ABP signal.  2. Detail coefficient selection: coefficients of interest were selected for feature extraction from the signal based on energy analysis, frequency analysis, and cross-correlation analysis. Detailed coefficients corresponded to particular levels of wavelet decomposition of the ABP signal  3. Window-based thresholding: the detail coefficients were used to identify peaks in the ABP signal, important peaks were isolated from irrelevant peaks using window-based thresholding.  4. Detection of maxima: valid maxima are identified using four conditions that correspond to the relative amplitude and temporal location of the peaks, redundancies are removed  5. Peak removal: thresholds for the temporal distance between peaks as well as the amplitude of the signal are used to remove errors. The result is a series of identified systolic peaks.  6. Detection of pulse onset and dicrotic notch: the average detail coefficient at the first level of decomposition is used in conjunction with the previously identified peaks. Window-based thresholding is used to segment the ABP signals and identify minima; redundant minima are removed which were determined based on the location of the identified systolic peaks. The result of this was the onset. The onset and the systolic points were used to determine the locations of the dicrotic notches.  7. Dicrotic peak detection: this involved finding a weak peak between the dicrotic notch and the onset of the next pulse.  **Validation:** Model was developed without training and was directly applied to a testing dataset | Compared to manual annotations of the various datasets, some of which had access to ECG signals. | The locations of the peaks, the pulse onsets, dichroitic notches, and dichroitic peaks were determined for each database, data for each of their efficacies were included in the paper. That of the MGH/MF database was used as there were 22 signals included (the most of any of the databases) where the method used the sym4 wavelet:  For 1,873 manual annotations of peak locations: Acc = 97.27%  Sens = 99.52%  PPV = 97.79%  Error = 2.67%  For 1,868 onsets manually detected: Acc = 97.96%  Sens = 99.62%  PPV = 98.36%  Error = 2.007%  For 1,860 manually identified dicrotic notches:  Acc = 98.54%  Sens = 99.35%  PPV = 99.19%  Error = 1.449% | The sym4 wavelet performed better than the db4 wavelet using the MGH/MF database; however, the db4 performed better using the MIT-BIH dataset  Strong validation of this method using data from several databases. | Only evaluated on one-minute data segments from patients due to the amount of time required to manually annotate it |
|  | **Sex (% Male)** | N/A |  |  |  |  |  |  |  |
|  | **Age** | N/A | **Device type** | N/A |  |  |  |  |  |
|  | **Pathophysiological health** | Signal with constant heart rate and arrythmias | **Location** | Aorta |  |  |  |  |  |
| Li et al. [102] | **Subjects** | 2 from CSL [44]  20 from Fantasia, accessible through Physionet [113]  16 from SLP [116] | **Signal type(s) used** | ABP (CSL) (100 Hz)  NIBP and ECG (Fantasia) (250 Hz)  IBP and ECG (SLP) (250 Hz) | The proposed algorithm was designed to detect the pulse onset, systolic blood pressure, and dicrotic notch of the arterial blood pressure signals. The raw signals are fed into the following pipeline:  1. Low-pass filtering: the raw ABP data is fed into a third order low-pass Bessel filter.  2. Derivative estimation: the first order amplitude difference is taken and is used as an estimation of the derivative of the ABP wave.  3. Estimation of thresholds: the ABP waveform is initially divided equally, a selective window is applied to the first two seconds of the ABP waveform, this window is used to adaptively update the amplitude and interval thresholds.  4. Zero-crossing points: these are located using the estimated derivative. The points of interest are the zero crosses that occur immediately before or after a maximum. These denote potential onsets and systolic points.  5. Beat evaluation: this is based on the comparison of the potential onsets and systolic points to the estimated thresholds. If they are deemed acceptable by the adaptive thresholds, the algorithm moves on. If not, the thresholds are adjusted, and this process is repeated.  6. Detecting inflection points: a searching window is used to find paired of inflection points and zero-crossing points  7. Evaluation of dicrotic notch selection: this is based off the relative location of the notch to the second inflection in the ABP pulse, as well as satisfying additional criteria.  **Validation:** Model was developed without training and was directly applied to a testing dataset | The Fantasia and SLP databases had not been annotated for their dicrotic notches, as such, these were annotated by a group of trained engineers, some of the datasets had simultaneously recorded ECG signals  Performance of previously developed methods including that of Aboy et al. [44] and Zong et al. [15] | The performance for beat detection using the CSL database (13,079 annotations) for the proposed methods:  Error = 0.34%  Sens = 99.82%  PPV = 99.84%  For Aboy et al.:  Error = 0.57%  Sens = 99.80%  PPV = 99.63%  The performance for beat detection using the Fantasia database (137,830 annotations) for the proposed methods:  Error = 1.56%  Sens =98.29 %  PPV = 99.98%  For Zong et al.:  Error = 2.78%  Sens = 98.95%  PPV = 98.26%  The performance for beat detection using the SLP database (318,412 annotations) for the proposed methods:  Error = 0.83%  Sens = 99.19 %  PPV = 99.99%  For Zong et al.:  Error = 0.66%  Sens = 99.84%  PPV = 99.50%  A subset of the Fantasia and SLP databases were used to develop the SLM database with annotation for onsets, systolic points, and dicrotic notches (2,564 annotations):  The performance for onsets, systolic points, and dicrotic notches, respectively were:  Error = 1.43%, 1.54%, 6.83%  Sens = 99.96%, 99.88%, 96.53%  PPV = 98.73%, 98.69%, 96.64% | Strong performance in detection of beats, less strong in dicrotic notch; but overall good performance. | Relatively small cohorts |
|  | **Sex (% Male)** | N/A (all) |  |  |  |  |  |  |  |
|  | **Age** | N/A (CSL)  Youth and elders (Fantasia)  32 to 56 (SLP) | **Device type** | N/A |  |  |  |  |  |
|  | **Pathophysiological health** | N/A | **Location** | N/A |  |  |  |  |  |
| Aguirre et al.  [117] | **Subjects** | MIMIC-III (number of subjects not mentioned) [119], 2 (CSL database) [44], 20 recordings (Fantasia database (FTS)) [113], [115], 16 (Polysom-nographic database (SLP)) [113], [116] | **Signal type(s) used** | ABP (125 Hz) some with annotation using ECG | Attempts to detect different aspects of the ABP morphological signal including the SBP, DBP, and dichroitic notch referred to in this work as the fiducial points (FiP). Initially, preprocessing and data augmentation were used to prepare the data such that the proposed method could be tested on a variety of artifact types. This involved:  1. Processing of data quality by discarding obvious artifacts and examining the validity of the heartbeats to detect other possible artifacts. Potential artifacts are flagged.  2. Data augmentation by applying random replacement of beats by artifacts and adding additional baseline functions like sigmoid or sine functions. This essentially mimicked artifacts.  Two models were analyzed for their utility in this application, a U-Net model initially proposed by Ronneberger et al. [156] and an extension of the U-Net model which incorporated an 8-layer temporal convolutional network (U-TCN) initially proposed by Bai et al. [157]. The U-Net model has a ‘contracting’ path and an ‘expansive’ path [156]. The contracting path is a convolutional neural network which uses repeated unpadded convolutions, a rectified linear unit operation, and a max pooling operation to extract important features from input signal images while compressing its size [117]. The expansive path up-samples the feature map and reduces the number of feature channels using a convolution. Skip connections help facilitate a more accurate reconstruction using saved information from the contracting path in the expansive portion of the U-Net architecture. The end-result of the U-Net is the classification of a point using the final feature map. [117]  The U-TCN method was an extension of this that retained sequence information. These models classify signal segments (such as the ‘dicrotic notch to onset interval’). The changes between classes of segments mark the FiP.  **Validation:** The MIMIC-III was preprocessed, and the dataset was divided into 72.5% for training, 12.5% for validation, 15% for testing. | Annotations were provided by public datasets  Pulse delineator (PUD) method proposed by Li et al. [102] | Sensitivity, PPV, and error rate were calculated for three data sets. The CSL and FTS (annotated heartbeats using QRS wave), as well as the SLP (annotations in ECG signal simultaneously recorded). The effectiveness of the PUD, U-Net, and U-TCN methods were examined.  Across the three datasets, the best by specificity and positive predictive value was U-TCN (average of 99.83 and 99.80, respectively). This method also had the lowest error rate (average of 0.833). However, all methods performed relatively well.  However, when applied to the set of preprocessed MIMIC-III data for evaluation, the PUD method vastly underperformed the other two methods in Se, PPV, and error rate. The U-Net method achieved the best error rate value in detecting the onset, peak, and dichroitic notch with results of 0.69, 0.49, and 1.98, respectively. | The FiPs were able to be differentiated from artifacts  Competitive in the success of the proposed method using established databases | Proposed methodology requires labeled data |
|  | **Sex (% Male)** | N/A |  |  |  |  |  |  |  |
|  | **Age** | N/A | **Device type** | N/A |  |  |  |  |  |
|  | **Pathophysiological health** | Admitted to ICU | **Location** | N/A |  |  |  |  |  |
| Kyle et al. [121] | **Subjects** | 350 | **Signal type(s) used** | ABP (Brachial pulse) (250 Hz) | This algorithm was designed to detect particular aspects of the brachial pulse signal including the beginning of the pulse (F), systolic peak (P), “incisura” (inflection at the end of the systolic phase) (I), dicrotic peak (local maximum after incisura)(D). Each pulse wave is processed and was only considered successfully processed if it had no artifacts and the algorithm was able to detect the P, I, and D points. The algorithm had the following steps:  1. Find the upslope that corresponds to systole for each of the pulse cycles using a threshold for the first difference. The threshold is adjusted if there are too few upslopes identified  2. Determine the location of the minimum for the slope and find the F point  3. The maximum point of the wave is set as point P  4. Find the downslope corresponding to the end of systole based on a threshold for the first difference.  5. Find onset of dicrotic wave using the next upslope, the local minimum is point I and the local maximum is point D. Variations in the pulse wave are handled using particular thresholds based on its time relative location to the F point. If it is not able to be found, the location is estimated using the change of slope method.  There were minimum and maximum thresholds for the distance between the F and P, F and I, and I and D points. If they were not within an acceptable range, they were discarded.  Any baseline drift is removed through detection using a line drawn through the minima of the slopes. Amplitudes of the P point, I point, and D point are calculated as well as the ratios between the amplitudes of the I and P points as well as the I and D points.  **Validation:** Model was developed without training and was directly applied to a testing dataset | Human observers | For each cycle there were several metrics calculated, and their results were compared to those generated by human observers. These included distance between F-F points, F-P points as a percentage of the total pulse, F-I points as a percentage of the total pulse, and I-D points as well as the previously mentioned amplitude ratios. Correlation (r) was calculated for the scatter plots of the results for the computer against the human observer. The results were as follow:  F-F: r=0.99  F-P (%): r=0.97  I/P: r=0.97  F-I (%): r=0.94  I/D: r=0.94  Algorithm was able to process 332 of 350 records | Good performance compared to human observers, discarding artifacts improved accuracy  Can be extended to intra-arterial signals | Sources of error associated with poor recording quality leading to an inability for the algorithm to properly detect all points  Population used was quite specific  Point D was estimated when it could not be directly identified |
|  | **Sex (% Male)** | 100% |  |  |  |  |  |  |  |
|  | **Age** | 15 to 88 | **Device type** | Pressure transducer |  |  |  |  |  |
|  | **Pathophysiological health** | Normal as well as with disease including hypertension, atherosclerosis, and diabetes | **Location** | Brachial artery |  |  |  |  |  |
| Singh and Sunkaria (2016) [122] | **Subjects** | 5 to 10 clean recordings selected (MIT-BIH Polysomno-graphic [113], [116])  40 (Fantasia [113], [115])  90 (MIMIC [113], [123]) | **Signal type(s) used** | ABP;  Invasiveness not indicated for MIT-BIH  Non-invasive for Fantasia (250 Hz)  Assumed to be invasive MIMIC | This algorithm uses the empirical wavelet transformation (EWT) to detect the onsets and systolic peaks of ABP data. The EWT is used to decompose the ABP signal into 5 different ‘modes’ (frequency components). The mode that aligns closest with the oscillations of the blood pressure is used for delineation of the waveforms. This is determined by comparing the central frequencies. The zero crossings of the identified mode are expected to align with the onsets of the blood pressure waveform. The maxima of the identified mode are expected to align with the systolic peaks. To precisely determine the location of either point of interest, a local search was conducted in the vicinity of the identified zero crossing and maxima in the identified mode. The minima of the ABP signal in the region of the zero crossing and the maxima in the region of the mode maxima were recorded as the final onset and systolic peak, respectively.  **Validation:** Model was developed without training and was directly applied to a testing dataset | Expert annotations provided by public datasets | For MIT-BIH, Fantasia, MIMIC, respectively:  Sens = 99.92%, 100%, 99.93% (onsets)  Sens = 99.96%, 99.97%, 99.95% (systolic peaks)  Error = 0.15%, 0.02%, 0.18% (onsets)  Error = 0.15%, 0.05%, 0.12% (systolic peaks)  PPV = 99.92%, 99.97%, 99.87% (onsets)  PPV = 99.88%, 99.97%, 99.91% (systolic peaks) | Algorithm achieved strong performance in identification of morphological features of ABP pulses  Used diverse dataset  Could be used for further waveform delineation for identification of more features | All data used was identified as being clean data  Only identifies two features of waveform |
|  | **Sex (% Male)** | N/A (MIT-BIH)  50% (Fantasia)  N/A (MIMIC) |  |  |  |  |  |  |  |
|  | **Age** | N/A (MIT-BIH)  20 between 21 and 34 years, 20 between 68 to 85 years (Fantasia)  N/A (MIMIC) | **Device type** | N/A |  |  |  |  |  |
|  | **Pathophysiological health** | Chronic obstructive sleep apnea (MIT-BIH), healthy (Fantasia), ICU (MIMIC) | **Location** | N/A |  |  |  |  |  |
| Pachauri and Bhuyan (2011) [125] | **Subjects** | Not specified, implied that 4,121 beats were annotated from the PhysioNet MGH/MF mgh001 dataset and others within this dataset | **Signal type(s) used** | ABP (360 Hz), ECG | This algorithm aims to detect systolic blood pressure peaks in the ABP signal and denoises. It accomplishes this using the following methodology:  1. Pre-processing: low-frequency artifacts are removed using a high-pass fourth-order Butterworth filter that has a cutoff frequency of 0.5 Hz.  2. Generation of energy signal for ABP: an energy signal is generated based on the ABP signal using a window of 100 ms with zero padding conducted on the edges of the signal. This gives the outputted energy signal the same length as the initial windowed signal. Scaling is then done to the ABP signal to accentuate the systolic peaks.  3. Thresholding to detect peaks: the windowed energy signal is then used to remove any extraneous peaks that are not that of systolic blood pressure. Values below a certain percentage of the maximum energy within a window are removed. An additional temporal threshold removes any peaks that are closer than 200 ms apart.  **Validation:** Model was developed without training and was directly applied to a testing dataset | Annotated pulses from database, it was indicated that it was required to have a simultaneously recorded ECG signal for validation | The proposed algorithm was able to obtain an accuracy of 99.53% from the mgh001 dataset. For all the pulses analyzed, there were 4,043 of 4,121 beats properly labeled. There were 78 peaks missing and 1 false beat.  Sens = 99.98%  PPV = 98.14% | Accurate performance in identifying systolic blood pressures in ABP signal | Small dataset that was not well detailed, difficult to generalize the results |
|  | **Sex (% Male)** | N/A |  |  |  |  |  |  |  |
|  | **Age** | N/A | **Device type** | N/A |  |  |  |  |  |
|  | **Pathophysiological health** | N/A | **Location** | N/A |  |  |  |  |  |
| Nygards et al. [106] | **Subjects** | 17 | **Signal type(s) used** | IBP (125 Hz), ECG, left ventricular pressure, PAP, right atrial pressure (250 Hz) | There were separate processes for the ECG, venous pressure, atrial pressure, and pulmonary pressure as well as their derived indices. This review will focus on the arterial pressure. The algorithm aimed to detect pre-ejection and left ventricular ejection times, SBP, DBP, MAP, and maximum pressure gradient as well the dicrotic notch  1. Low-pass digital filter  2. Onset of the R-wave from the ECG signal which is detected using the first derivative (first difference of ECG signal) and a defined threshold which is initially defied as 2/3 the of the maximum value of the signal in the first 5 seconds of recording, QRS complex located as 40 ms plateau before R-wave where the absolute values between each data point is below a particular threshold. This process is repeated to determine all QRS complexes  3. Threshold established for ABP signal following a similar methodology as for R-wave preceding step where the threshold is set as half of the maximum first derivative in the first 5 seconds.  4. Based on location of reference point in QRS wave, systolic upslope is located when a derivative greater than the defined threshold is found.  5. Estimation of second derivative computed based on a second difference equation, the time of ejection is determined as the nearest point which has a second derivative maximum within 250 ms. Its absence results in a new search beginning from the next QRS point.  6. A 90 ms search interval for the dicrotic notch is identified using an approximation based on the heart rate beginning 30 ms prior to the approximated location of the notch. A maximum in the second derivative of the signal exists before and after the notch is used to define an initial estimation for the notch. This is then refined using another second difference equation where a peak indicates the dicrotic notch which indicates the end of systolic ejection.  **Validation:** Model was developed without training and was directly applied to a testing dataset | Compared to results of trained physician using ECG, PAP, right atrial pressure, and left ventricular pressure signals | The results of the aortic pressure measurements were compared using the difference between the physician and computer as their mean difference ± SD  SBP: 1.3 ± 3.8 mmHg  MAP: -0.9 ± 2.5 mmHg  DBP: -0.4 ± 2.0 mmHg  Pre-ejection time: 20 ± 8 ms  Left ventricular ejection time: -15 ± 8 ms | Decent performance by the computer in identifying characteristic points in an aortic blood pressure recording | Algorithm struggled with the temporal locations of the pre-ejection and ejection times. Additionally, there were issues with locating the systolic onset  Insufficient to function in the presence of artifacts or arrythmias  Only used 20 second recordings |
|  | **Sex (% Male)** | N/A |  |  |  |  |  |  |  |
|  | **Age** | N/A | **Device type** | Catheter |  |  |  |  |  |
|  | **Pathophysiological health** | N/A, some exercising | **Location** | Aorta |  |  |  |  |  |

*ABP = Arterial Blood Pressure, Acc = Accuracy, APW = Arterial Pressure Waveform, CBFV = Cerebral Blood Flow Velocity, , D = Dicrotic Peak, DN = Dicrotic Notch, DP = Dicrotic Peak, ECG = Electrocardiogram, EWT = Empirical Wavelet Transform, FiP = Fiducial Point, FFT = Fast Fourier Transform, FTS = Fantasia Test Set, IBP = Invasive Blood Pressure, ICP = Intracranial Pressure, LVET = Left Ventricular Ejection Time, NIBP = Non-Invasive Blood Pressure, NPH = Normal Pressure Hydrocephalus, PAP = Pulmonary Arterial Pressure, PPA = Positive Predictive Accuracy, PEP = Pre-Ejection Period, PPV = Positive Predictive Value, PUD = Pulse Delineator, QS = Electromechanical Systole, r = Correlation Coefficient, r² = Coefficient of Determination, RP = Reflection Point, SAH = Subarachnoid Hemorrhage, Sens = Sensitivity, SG filter = Savitzky-Golay Filter, SP = Systolic Peak, SSF = Slope Sum Function, U-TCN = U-Net with Temporal Convolutional Network*

**Table E.5 Other point identification**

| **Reference** | **Subject information** | | **Data type (sampling rate) – system type** | | **Artifact removal method(s) used** | **Methods compared** | **Effectiveness** | **Study results and conclusions** | **Limitations** |
| --- | --- | --- | --- | --- | --- | --- | --- | --- | --- |
| Swatzell at al. [126] | **Subjects** | 2,000 | **Signal type(s) used** | ABP (carotid pulse)(250 Hz), vectorcardiogram (Frank orthogonal X,Y,Z leads) and heart sounds (500 Hz) | This algorithm was developed to identify the systolic time in carotid pulses. This involves the identification the upstroke in the carotid pulse as well as the incisura notch; however, it was also designed to identify the onset of the QRS complex and the second Korotkoff sound.  1. QRS onset detection: the onset of the QRS is located using the spatial magnitude curve generated using the X, Y, and Z leads in the corrected Frank orthogonal system.  2. Carotid upstroke detection: the location of the Q in the QRS complex is the start point for the search for the carotid peak. The pulse signal is ‘desloped’ between $Q_{i}$ and $Q_{i+1}-1$, the maximum value within this range is the carotid peak. A line is drawn between $Q_{i}$ and the carotid peak. The greatest difference between the signal and the aforementioned line is the carotid upslope.  3. Carotid incisura notch: similarly, a line is drawn between the identified peak and the point ${(Q}_{i+1}-1-peak)/2$ and again the difference between the signal and this line is taken. The point with the greatest difference is the identified carotid incisura notch. Errors that occur due to the presence of multiple notches can be adjusted and corrected by observers to have the algorithm ‘learn’. The algorithm attempts to learn from these changes.  4. Second heart sound: a search is conducted using the carotid incisura notch as a reference point, considering 0.08 seconds before the notch to the location of the notch. The first 5 points in the area are used to establish a baseline. The location of the greatest deflection of the second heart sound is determined. The second heart sound is normalized, and derivative analysis is used to identify the exact location of the second heart sound.  **Validation:** Model was developed without training and was directly applied to a testing dataset | Human identification of points | The waveform identification method proposed was able to achieve an identification accuracy of 95% | Able to identify waveforms with good accuracy  Allows for human input for optimization and limiting of errors | Requires human input for optimization  Not much information regarding the specific identification of carotid waveforms |
|  | **Sex (% Male)** | N/A |  |  |  |  |  |  |  |
|  | **Age** | N/A | **Device type** | Glycerin pellet connected to Statham PM5-0.2-350 transducer |  |  |  |  |  |
|  | **Pathophysiological health** | N/A | **Location** | Carotid |  |  |  |  |  |
| Almeida et al. [127] | **Subjects** | 8 | **Signal type(s) used** | ABP (referred to as arterial pressure waveform (APW) (1,000 Hz) | The proposed Prominent Points Identifier Algorithm (PPIA) is designed to identify specific points along the APW including the systolic peak (SP), reflection point (RP), dicrotic peak (DP), and dicrotic notch (DN). This algorithm involves the following steps:  1. Pulses are segmented (methodology followed is not listed)  2. Low-pass (50 Hz) filter is applied to make points more identifiable  3. Local extrema are identified, which are the SP  4. First derivative of each pulse is calculated and the locations of zero-crossing of this first derivative are determined. These zero-crossings and the amplitude values of the original signal are used to determine the location of the dicrotic notch and the dicrotic peak.  5. The “type” of APW are separated into four groups based on criteria is defined using the morphology and number of peaks observed in the pulse.  6. This information is then used to determine the location of the RP. The RP is the process of blood backflow.  This information regarding the locations of the identified prominent points was then used to determine augmentation index (AI), a measure of arterial stiffness.  **Validation:** Model was developed without training and was directly applied to a testing dataset | A human expert engineer identified the points, the TP, FN, and FP were taken in consideration with an 8 ms threshold used in previous literature. | The temporal locations of the four points of interest had average values for error, sensitivity, and positive predictive value of 4.20%, 99.09%, and 96.77%, respectively.  The amplitude locations of the four points of interest had average values for error, sensitivity, and positive predictive value of 2.68%, 99.08%, and 98.22%, respectively.  Correlation between human and algorithm for SP and RP were r^2^=0.996 and 0.907, respectively. | Algorithm was able to identify points of interest in APW with good accuracy compared to human | Small dataset, limits generalizability  Struggled with the identification of the RP point  Not much information regarding the methodology of most of the steps of the algorithm  Baseline wander is removed by device itself |
|  | **Sex (% Male)** | N/A |  |  |  |  |  |  |  |
|  | **Age** | N/A | **Device type** | Custom non-invasive piezometric probe with built in baseline wander removal circuit |  |  |  |  |  |
|  | **Pathophysiological health** | N/A | **Location** | Neck (carotid artery) |  |  |  |  |  |
| Paradkar and Chowdhury [128] | **Subjects** | 100 from MIMIC-II [129] + 13,079 pulses from CSL Benchmark [44] | **Signal type(s) used** | ABP (125 Hz) | Detection of components within the ABP waveform including the systolic peak, percussion peak, tidal peak, and diastolic peak. The steps involve:  1. Pre-processing to remove noise and pulse segmentation through local peak estimation  2. Extraction of periodic components using singular value decomposition (SVD) to estimate the most dominant periodic component using a method initially proposed by Kanjilal et al. [160] by observing the dominant pulse from 10 pulses  3. Continuous wavelet transformation using the Mexican hat wavelet using four scales, the coarsest of which are used to detect systolic and diastolic phases.  4. Wavelet coefficient peaks are used to identify peaks  **Validation:** The MIMIC-II served as the training set, the CSL served a testing set | Compared to CSL Benchmark database annotations [44] | Evaluated using the training dataset developed using the MIMIC-II database [129].  There were 13,079 annotated pulses tested, 12,979 correctly annotated, 110 incorrectly annotated, 100 undetected.  Algorithm had a sensitivity of 0.9924 and a PPV of 0.9848 for the CSL Benchmark. | Generally, strong performing in identification of features  Reduces the effect of noise.  Could be applied to ICP or PPG signals but has not been validated. | The algorithm struggles when the P, T, or D peaks are absent or not clear  Not tested across clearly described injury patterns or demographics  No evaluation of other wavelet types |
|  | **Sex (% Male)** | N/A |  |  |  |  |  |  |  |
|  | **Age** | N/A | **Device type** | N/A |  |  |  |  |  |
|  | **Pathophysiological health** | Admitted to ICU | **Location** | N/A |  |  |  |  |  |
| Prakash et al. [130] | **Subjects** | 120 | **Signal type(s) used** | NIBP, PPG | This algorithm was designed for feature extraction and peak detection for PPG and radial pulses. For the purposes of this work, the radial pulse information will be described in detail. A multi-resolution decomposition was conducted using the Daubechies 9 (D9) wavelet. The levels of decomposition if the signal were isolated. Levels D1-D3 were removed as they represented noise. Levels D3-D5 were indicated to be representative of the true radial pulse. These elements were amplified using squaring such that peaks could be easily detected.  **Validation:** Model was developed without training and was directly applied to a testing dataset | Manual counting of pulses, likely with access to PPG signal | This method had an accuracy of 97.89% based on what was presumed to be the same 120 subjects used for the PPG; however, it is not directly stated | It is indicated that this method was able top detect the percussion wave, tidal wave, and dicrotic notch; however, it was not clear with what success rate | Developed for a non-standard recording method  Only developed for short recording periods under controlled conditions with healthy subjects |
|  | **Sex (% Male)** | N/A |  |  |  |  |  |  |  |
|  | **Age** | N/A | **Device type** | Nadi Yantra device for radial pulse [161] |  |  |  |  |  |
|  | **Pathophysiological health** | Healthy | **Location** | Radial artery |  |  |  |  |  |
| Starmer et al. [105] | **Subjects** | 4 mongrel dogs | **Signal type(s) used** | IBP (200 Hz), aortic blood flow, ECG | The developed algorithm was intended to extract the onset and the end of ventricular ejection. It was intended to identify the upstroke and the dicrotic notch of a given blood pressure pulse. It first required that the data be divided into segments each containing a single pulse. This was accomplished using the location of the R-wave in the ECG data, as the ventricle is expected to contract within 50 to 200 ms after this. The R-wave was detected using the absolute value of the first derivative of the ECG signal. Exceeding a particular threshold, which was based off of the maximum least-squares estimate of the first derivative of the first 2 seconds of recording, indicated the R-wave. Proceeding this, the ejection onset location was estimated when the BP signal satisfied the following criteria: (1) four consecutive first derivatives being greater than 10 mmHg/sec (2) having those first derivatives occurring between 30 and 200 ms after the R-wave. The absence of an ejection onset either indicates the lack of a mechanical process occurring as a result of the R-wave or that it indicated an artifact in the ECG signal. This process is then repeated 80 ms after the identified R-wave if no onset is found. Systolic pressure is then identified as the maximum average of 5 sequential points between the identified onset of ejection and 200 ms after. To determine the location of the dicrotic notch, the least-squares estimate of the second derivative is calculated between 30 ms and 300 ms after the point of the systolic blood pressure, the dicrotic notch is estimated as the minimum within the interval of the maximum second derivative  **Validation:** Model was developed without training and was directly applied to a testing dataset | Compared to ejection interval index points obtained from aortic blood flow data using a previously described algorithm by Benson et al. [162] | The most substantial differences in the ejection onset was found to be 9 ± 1 ms across the 4 dogs examined with an average of 166 beats examined per dog.  The most substantial difference in the ejection end was -7 ± 1 ms | The proposed algorithm was able to accurately identify ejection indices and was able to do so faster than the Benson et al. algorithm which uses aortic blood flow | Non-human subjects  Provided no analysis of the accuracy of the systolic blood pressure or dicrotic notch locations |
|  | **Sex (% Male)** | N/A |  |  |  |  |  |  |  |
|  | **Age** | N/A | **Device type** | High fidelity pressure catheter, electromagnetic flow meter probe |  |  |  |  |  |
|  | **Pathophysiological health** | N/A | **Location** | Aorta |  |  |  |  |  |

*ABP = Arterial Blood Pressure, AI = Augmentation Index, APW = Arterial Pressure Waveform, BP = Blood Pressure, DBP = Diastolic Blood Pressure, DN = Dicrotic Notch, DP = Dicrotic Peak, ECG = Electrocardiogram, FN = False Negative, FP = False Positive, Hz = Hertz, ICP = Intracranial Pressure, ICU = Intensive Care Unit, PPG = Photoplethysmogram, PPV = Positive Predictive Value, PPIA = Prominent Points Identifier Algorithm, RP = Reflection Point, SP = Systolic Peak, SVD = Singular Value Decomposition, TP = True Positive*

References

[134] D. A. Winter, *Biomechanics and Motor Control of Human Movement*, 1st ed. Wiley, 2009. doi: 10.1002/9780470549148.

[135] G. E. Box, G. M. Jenkins, and G. C. Reinsel, *Time series analysis: forecasting and control*, vol. 734. John Wiley & Sons, 2011.

[136] B. Schölkopf, J. C. Platt, J. Shawe-Taylor, A. J. Smola, and R. C. Williamson, “Estimating the Support of a High-Dimensional Distribution,” *Neural Computation*, vol. 13, no. 7, pp. 1443–1471, Jul. 2001, doi: 10.1162/089976601750264965.

[137] F. T. Liu, K. M. Ting, and Z.-H. Zhou, “Isolation Forest,” in *2008 Eighth IEEE International Conference on Data Mining*, Pisa, Italy: IEEE, Dec. 2008, pp. 413–422. doi: 10.1109/ICDM.2008.17.

[138] A. E. W. Johnson *et al.*, “MIMIC-IV, a freely accessible electronic health record dataset,” *Sci Data*, vol. 10, no. 1, p. 1, Jan. 2023, doi: 10.1038/s41597-022-01899-x.

[139] I. Higgins *et al.*, “β-VAE: Learning basic visual concepts with a constrained variational framework,” presented at the ICLR, 2017.

[140] S. Hochreiter and J. Schmidhuber, “Long Short-Term Memory,” *Neural Computation*, vol. 9, no. 8, pp. 1735–1780, Nov. 1997, doi: 10.1162/neco.1997.9.8.1735.

[141] M. Geurts, G. E. P. Box, and G. M. Jenkins, “Time Series Analysis: Forecasting and Control,” *Journal of Marketing Research*, vol. 14, no. 2, p. 269, May 1977, doi: 10.2307/3150485.

[142] H.-J. Lee, E.-J. Jeong, H. Kim, M. Czosnyka, and D.-J. Kim, “Morphological Feature Extraction From a Continuous Intracranial Pressure Pulse via a Peak Clustering Algorithm,” *IEEE Trans. Biomed. Eng.*, vol. 63, no. 10, pp. 2169–2176, Oct. 2016, doi: 10.1109/TBME.2015.2512278.

[143] M. A. Oskoei and Huosheng Hu, “Support Vector Machine-Based Classification Scheme for Myoelectric Control Applied to Upper Limb,” *IEEE Trans. Biomed. Eng.*, vol. 55, no. 8, pp. 1956–1965, Aug. 2008, doi: 10.1109/TBME.2008.919734.

[144] A. Widodo and B.-S. Yang, “Support vector machine in machine condition monitoring and fault diagnosis,” *Mechanical Systems and Signal Processing*, vol. 21, no. 6, pp. 2560–2574, Aug. 2007, doi: 10.1016/j.ymssp.2006.12.007.

[145] A. I. R. Maas *et al.*, “Collaborative European NeuroTrauma Effectiveness Research in Traumatic Brain Injury (CENTER-TBI): A Prospective Longitudinal Observational Study,” *Neurosurgery*, vol. 76, no. 1, pp. 67–80, Jan. 2015, doi: 10.1227/NEU.0000000000000575.

[146] J. Lin, E. Keogh, S. Lonardi, and B. Chiu, “A symbolic representation of time series, with implications for streaming algorithms,” in *Proceedings of the 8th ACM SIGMOD workshop on Research issues in data mining and knowledge discovery*, San Diego California: ACM, Jun. 2003, pp. 2–11. doi: 10.1145/882082.882086.

[147] F. Scholkmann, J. Boss, and M. Wolf, “An Efficient Algorithm for Automatic Peak Detection in Noisy Periodic and Quasi-Periodic Signals,” *Algorithms*, vol. 5, no. 4, pp. 588–603, Nov. 2012, doi: 10.3390/a5040588.

[148] H. Sorvoja, R. Myllya, P. Karja-Koskenkari, J. Koskenkari, M. Lilja, and A. Kesaniemi, “Accuracy comparison of oscillometric and electronic palpation blood pressure measuring methods using intra-arterial method as a reference,” *Molecular Quantum Acoustics*, vol. 26, pp. 235–260, 2005.

[149] D. K. Park, H. S. Oh, J. H. Kang, I. Y. Kim, Y. J. Chee, and J. S. Lee, “Novel method of automatic auscultation for blood pressure measurement using pulses in cuff pressure and korotkoff sound,” in *2008 Computers in Cardiology*, Bologna, Italy: IEEE, Sep. 2008, pp. 181–184. doi: 10.1109/CIC.2008.4749007.

[150] W. Zong, G. B. Moody, and R. G. Mark, “Reduction of false arterial blood pressure alarms using signal quality assessement and relationships between the electrocardiogram and arterial blood pressure,” *Med. Biol. Eng. Comput.*, vol. 42, no. 5, pp. 698–706, Sep. 2004, doi: 10.1007/BF02347553.

[151] X. Hu, P. Xu, D. J. Lee, P. Vespa, K. Baldwin, and M. Bergsneider, “An algorithm for extracting intracranial pressure latency relative to electrocardiogram R wave,” *Physiol. Meas.*, vol. 29, no. 4, pp. 459–471, Apr. 2008, doi: 10.1088/0967-3334/29/4/004.

[152] X. Hu, P. Xu, F. Scalzo, P. Vespa, and M. Bergsneider, “Morphological Clustering and Analysis of Continuous Intracranial Pressure,” *IEEE Trans. Biomed. Eng.*, vol. 56, no. 3, pp. 696–705, Mar. 2009, doi: 10.1109/TBME.2008.2008636.

[153] M. Cannesson *et al.*, “Machine learning of physiological waveforms and electronic health record data to predict, diagnose and treat haemodynamic instability in surgical patients: protocol for a retrospective study,” *BMJ Open*, vol. 9, no. 12, p. e031988, Dec. 2019, doi: 10.1136/bmjopen-2019-031988.

[154] J. Balmer *et al.*, “Electrocardiogram R-wave is an Unreliable Indicator of Pulse Wave Initialization,” *IFAC-PapersOnLine*, vol. 50, no. 1, pp. 856–861, Jul. 2017, doi: 10.1016/j.ifacol.2017.08.254.

[155] J. Pan and W. J. Tompkins, “A Real-Time QRS Detection Algorithm,” *IEEE Trans. Biomed. Eng.*, vol. BME-32, no. 3, pp. 230–236, Mar. 1985, doi: 10.1109/TBME.1985.325532.

[156] O. Ronneberger, P. Fischer, and T. Brox, “U-Net: Convolutional Networks for Biomedical Image Segmentation,” 2015, *arXiv*. doi: 10.48550/ARXIV.1505.04597.

[157] S. Bai, J. Z. Kolter, and V. Koltun, “An Empirical Evaluation of Generic Convolutional and Recurrent Networks for Sequence Modeling,” 2018, *arXiv*. doi: 10.48550/ARXIV.1803.01271.

[158] T. Heldt, M. B. Oefinger, M. Hoshiyama, and R. G. Mark, “Circulatory response to passive and active changes in posture,” in *Computers in Cardiology, 2003*, Thessaloniki Chalkidiki, Greece: IEEE, 2003, pp. 263–266. doi: 10.1109/CIC.2003.1291141.

[159] Y. C. Chiu, P. W. Arand, S. G. Shroff, T. Feldman, and J. D. Carroll, “Determination of pulse wave velocities with computerized algorithms,” *American Heart Journal*, vol. 121, no. 5, pp. 1460–1470, May 1991, doi: 10.1016/0002-8703(91)90153-9.

[160] P. P. Kanjilal, S. Palit, and G. Saha, “Fetal ECG extraction from single-channel maternal ECG using singular value decomposition,” *IEEE Trans. Biomed. Eng.*, vol. 44, no. 1, pp. 51–59, Jan. 1997, doi: 10.1109/10.553712.

[161] Abhinav, M. Sareen, M. Kumar, S. Anand, A. Salhan, and J. Santhosh, “Nadi Yantra: A Robust System Design to Capture the Signals from the Radial Artery for Non-Invasive Diagnosis,” in *2008 2nd International Conference on Bioinformatics and Biomedical Engineering*, Shanghai, China: IEEE, May 2008, pp. 1387–1390. doi: 10.1109/ICBBE.2008.676.

[162] D. W. Benson, “An algorithm for defining the cardiac cycle using ascending aortic blood flow,” *Computers and Biomedical Research*, vol. 4, no. 1–2, pp. 216–223, Apr. 1971, doi: 10.1016/0010-4809(71)90056-5.
